# Supplementary material for: Proteome-scale recombinant standards and a robust high-speed search engine to advance cross-linking MS-based interactomics
Source: Nat Methods. 2024 Oct 31;21(12):2327–35. doi: 10.1038/s41592-024-02478-1 (PMC11621016; doi:10.1038/s41592-024-02478-1)
Supplement: Supplementary file 1 — Supplementary Notes 1–6, Tables 1–3 and refs. [file 41592_2024_2478_MOESM1_ESM.pdf]

nature methods

Supplementary information https://doi.org/10.1038/s41592-024-02478-1

Proteome-scale recombinant standards and a robust high-speed search engine to advance cross-linking MS-based interactomics

In the format provided by the authors and unedited

Table of Contents

Supplementary Note 1. Overview of the SCOUT workflow 2

Supplementary Note 2. Scout's Scoring System 4

Supplementary Note 3. Multi-tier FDR filtering. 7

Supplementary Note 4. Enhancing data analysis through multilayer perceptron artificial neural networks 8

Supplementary Note 5. Extended description of the biological XL-MS results 11

Supplementary Note 6. Additional considerations in the design of the analytical standard 13

Supplementary Tables 14

Supplementary References 17

SUPPLEMENTARY NOTE 1. OVERVIEW OF THE SCOUT WORKFLOW

We present Scout, a cross-link search engine tailored toward identifying peptides cross-linked with cleavable reagents and analyzed by tandem mass spectrometry (MS2). Scout uses artificial neural networks (ANN) to rank identifications at the cross-linked spectrum match (CSM), ResPair, and protein-protein interaction (PPI) levels based on quality metrics optimized for each tier.

Scout requires a sequence database provided in the FASTA format; sequences will be processed in batches to optimize computer memory usage. Subsequently, all MS2 spectra will be automatically deconvoluted using the embedded Y.A.D.A 3.0[1]. Scout's core workflow consists of four stages: 1) ion pair doublet searching, 2) fast CSM searching, 3) refined spectrum scoring, and 4) multi-tier FDR filtering. This process is summarized in Figure 1 in the manuscript.

ION PAIR DOUBLET SEARCHING

This step aims to generate a list of candidate masses for the two linked peptides (α and β peptides) using information provided in the MS2 spectra. We define an ion pair as two spectral peaks whose mass difference equals to the asymmetric fragmentation at the cross-linker's cleavable moiety[2]. The two ion pairs, which we refer to as ion doublets, indicate the presence of a cross-linked α and β peptide pair in the spectrum. To optimize the search for ion pair doublets computationally, we linearly iterate over the ions in the first half of the spectrum to search for ion pairs and perform binary searches over the second half to find the complementary pairs. Scout can search for ion pairs in multiple charge/isotope states, but by default the algorithm applies spectra deconvolution with Y.A.D.A 3.0 prior to searching for ion pairs.

FAST CSM SEARCHING

The aim of this step is to perform a preliminary evaluation of the peptide candidates for each ion pair. The Fast CSM searching functions as a modified version of traditional shotgun database searching, but instead of using MS1 precursors as in conventional methods, the ion pair masses are used to calculate the precursor mass of each linked peptide. For fragment matching, Scout employs a spectral angle to score the match between the experimental spectrum and the theoretical one obtained from in silico fragmentation. This approach allows for a preliminary evaluation of peptide candidates for each ion pair.

Subsequently, Scout performs a second pass scoring at the CSM level by considering the top-n peptides for each of the α and β peptides in a combinatorial manner. For example, if three top peptide candidates are considered for α and β, a total of nine peptide combinations will be tested. The highest-scoring combination is selected as the CSM for that spectrum. Peptide combinations are evaluated according to the Decoupled Dot Product score (see Supplementary Note 2).

REFINED SPECTRUM SCORING

Once the high-scoring peptides for each ion pair are shortlisted, Scout proceeds to more sensitive and computationally intense scoring metrics. These methods compute ion pairs and chimeric CSM-level scores that consider both peptides concurrently. These combined evaluations offer a comprehensive scoring system that ensures the most accurate peptide selection. Further details about these scoring metrics can be found in Supplementary Note 2.

MULTI-TIER FDR FILTERING

Scout employs a multi-tiered approach in data filtering, using artificial neural networks (ANNs) to generate discriminant functions at three levels: cross-linked spectrum match (CSM), ResPair, and protein-protein interaction (PPI). These functions provide scores that are used to rank the identifications, with the ranking scores propagating from one level to the next. ANNs are trained using a binary classification system, where identifications are considered correct only when both peptides are targets, with all other cases labeled as incorrect. Each level of identification is then filtered according to a user-defined false discovery rate (FDR). This step is further described in Supplementary Note 4.

SUPPLEMENTARY NOTE 2. SCOUT'S SCORING SYSTEM

FAST CSM SEARCH METRICS

Spectral Angle

The Spectral Angle is a measure that compares the similarity between the theoretical and experimental spectral data. This comparison is achieved by first mapping these spectra onto vectors of equal dimensions, a process known as binning. In binning, a mass spectrum is divided into multiple *m/z* windows, and the intensities of all ions within each window are summed up to generate a vector. The Spectral Angle is then calculated by taking the dot product of these normalized vectors, which involves multiplying corresponding entries in the two vectors and summing the results.

Mathematically, if 'n' defines the vector length, the Spectral Angle is the sum of the dot products of the corresponding entries of the binned theoretical spectrum 'T' and the experimental spectrum 'E', as follows:

$$1)\ Spectral\ Angle = \sum_{i=0}^{n} (T_i * E_i)$$

The candidate peptides for each ion pair are then ranked according to their Spectral Angle scores. The top scoring candidates are selected as the ion pair matches.

Decoupled Dot Product

This score represents an enhanced version of the Spectral Angle, maintaining a similar calculation methodology. Similar to Spectral Angle, Decoupled Dot Product relies on a binning operation applied to both experimental and theoretical spectra, followed by the computation of the dot product of the normalized vectors. The key distinction lies in the treatment of shared fragments between α and β peptides. In Decoupled Dot Product, shared fragments are excluded during the binning process to ensure that the selection of high-scoring peptides is not unduly influenced by these fragments.

REFINED SPECTRUM SCORING METRICS

MinGNB Score

In the Fast CSM Searching step, we initially pinpoint the top peptide candidates for each ion pair using a swift Spectral Angle calculation (see above). To enhance this evaluation, we introduce a

more sophisticated but computationally demanding score, MinGNB. This process involves the addition of two more metrics at the peptide level: ΔCN and TagScore, both described below, alongside the previously computed Spectral Angle. These three features are input into a Gaussian Naïve Bayes (GNB) model, which generates a combined score. The GNB model is a probabilistic model that gauges the ability of these three scores to jointly differentiate correct matches from incorrect ones, assuming that each score independently contributes to the probability of a correct spectrum pair. match. The MinGNB is equal to the minimum score when considering an α and β peptide This approach empowers Scout to accurately and efficiently identify the most likely ion pair and its corresponding cross-linked peptides.

ΔCN: This represents the normalized score difference between the two top-scoring candidate peptides, calculated separately for each of the α and β peptides within a given ion pair. The calculation is as follows:

$$2)\ \Delta CN = \frac{\text{Best scoring peptide} - Second\ \text{Best scoring peptide}}{\text{Best scoring peptide}}$$

Tag Score: This score presents the sum of the length of unique tags that cover more than three (consecutive) amino acids. This score relies on the assumption that correct identifications will have greater coverage of consecutive fragment ions.

Given a peptide sequence P = ANEALKELVK with N = 10 amino acids, and a set of matched fragment ions F = {AN, ANE, ANEA, ANEAL, KELV} from the experimental spectrum. The tag score for P is calculated as follows:

Determine all possible consecutive fragment ion tags with a minimum length of 3:

T = {ANEAL, KELV}

Calculate the length of each tag in T:

|ANEAL| = 5

|KELV| = 4

Compute the tag score, S, as the sum of the lengths of all tags in T:

S = |ANEAL| + |KELV| = 5 + 4 = 9

Poisson Score

The Poisson score leverages the Poisson distribution to model the probability of a random match for a given number of matched fragments (in our case, non-shared fragments between the two linked peptides). It incorporates various factors such as the number of ions present in both experimental and theoretical spectra, the length of the peptide, and the mass tolerance of the

mass spectrometer. The final CSM-level Poisson Score for the cross-link is determined by selecting the better score between the α and β peptides.

Let:

- *p(n):* complement probability mass function for a Poisson distribution
- *n:* number of matching fragments
- *x:* probability of a random match between theoretical and experimental spectra
- *f:* total number of fragments for each linked peptide
- *frags:* number of fragments from each peptide bond
- m_t: user defined mass tolerance
- $l_{\alpha/\beta}$: length of α and β peptides, respectively.
- f_{total}: total number of MS2 fragment ions in the experimental spectrum.

Then:

$$3)\ p(n) = 1 - \sum_{i=0}^{n-1} \frac{e^{-xf}(xf^{i})}{i!},$$

where

$$4)\ x = \frac{1}{111.1} * frags\ * (m_t * 2)$$

and

$$5)\ f = \frac{l_\beta}{l_\alpha + l_\beta} *\ f_{total}$$

The score for each α and β peptide is given as follow:

$$6)\ Peptide\ Poisson\ Score\ \alpha|\beta = \log(p(n_{\alpha|\beta})))$$

And finally, the Poisson score for a CSM is computed as:

$$7)\ CSM\ Poisson\ Score = \min\ (Peptide\ Poisson\ Score_\alpha, Peptide\ Poisson\ Score_\beta).$$

Differential ppm error

The absolute difference between the mass errors of α and β peptides, measured in ppm.

$$8)\ Differential\ ppm\ error = |AlphaPPM\ Error - BetaPPM\ Error|$$

SUPPLEMENTARY NOTE 3. MULTI-TIER FDR FILTERING.

Scout encodes each spectrum as a vector, with elements corresponding to scores computed at the CSM, ResPair, or PPI levels. These vectors are labeled as either target or decoy, reflecting the identification of the corresponding spectrum. This setup enables the creation of a discriminant model, which uses these scores to rank identifications based on their likelihood of correctness. Scout employs three independent feedforward neural networks to model the data, as detailed in Supplementary Note 4. These networks, implemented via the Scikit-learn machine learning library, generate discriminant functions at the CSM, ResPair, and PPI levels. The ranking scores of the vectors for each tier are incorporated into the corresponding vectors and propagated across these tiers. Scout's multi-tier FDR is fine-tuned using a subset of the XL-MS standard dataset, which includes cross-linking data from 64 proteins across 8 interaction groups. During neural network training, identifications are considered correct (i.e., target) only when both peptides are targets. Each identification tier is filtered according to a user-defined FDR, with only identifications surpassing this threshold used in subsequent tiers.

CSMs that meet the classification score threshold set by the FDR are grouped into ResPairs, defined as unique residue-to-residue connections. Most features at the ResPair level are derived directly from the CSM level scores, primarily consisting of the best value of a given score among the grouped CSMs. The residue-pair features include:

- **Top Classification Score** – Highest classification score provided by the CSM level ANN amongst the grouped CSMs.
- **Top Poisson Score** – Highest Poisson score amongst the grouped CSMs.
- **Top Decoupled Dot Product** – Highest decoupled dot product amongst the grouped CSMs.
- **CSM Count** – Total number of CSMs that map to the residue-pair.

ResPairs passing the set FDR cutoff are grouped into PPIs, which are also scored with the aid of an ANN and are subjected to PPI FDR filtering. The PPI features include:

- **Top Classification Score** – Highest classification score provided by the ResPair level ANN amongst the grouped ResPairs.
- **Link Sites from Protein A to Protein B** – The total number of the unique linked sites in Protein A that link to Protein B.
- **Link Sites from Protein B to Protein A** – The total number of the unique linked sites in Protein B that link to Protein A.

Supplementary Note 4. Enhancing data analysis through multilayer perceptron artificial neural networks

Multilayer Perceptron (MLP) classifiers, a type of Artificial Neural Network (ANN), are employed for supervised learning tasks such as classification and regression. These networks consist of an input layer, one or more hidden layers, and an output layer, each containing interconnected neurons or nodes with associated weights. MLPs function defines a feedforward mechanism, where data flows unidirectionally from the input layer, through the hidden layers, to the output layer, without forming loops. During the training process, a backpropagation algorithm adjusts the connection weights to minimize the error between predicted and actual outputs.

In this study, we applied an MLP-based ANN to rank input vectors from a mass spectrometry dataset. To achieve this, we developed the MLP-ANN to generate a score for each input vector, *i.e.*, a vector with scores obtained from a given mass spectrum, considering input features for specific levels (*i.e.*, CSM, ResPair, or PPI). Once trained, the ANN assigned scores to input vectors, which were then used for ranking mass spectra according to confidence. Leveraging an MLP for ranking allows us to take advantage of its ability to capture intricate, nonlinear relationships between input features and output categories, often leading to improved ranking performance compared to traditional linear methods.

Scout relies on ANNs to rank identifications at the CSM, ResPair, and PPI levels utilizing quality metrics optimized for each respective tier. We used data from 64 proteins of our XL-MS standard to fine-tune our ANN setup and its hyperparameters, to reduce overfitting. Overfitting occurs when an ANN becomes too specialized, providing results that seem exceptional but do not generalize well; in this context, this could lead to reporting many identifications that do not align with our ground of truth dataset.

To further prevent overfitting, the MLP-ANN employs L2 regularization, which introduces a penalty to the model's loss function based on the sum of the squares of connection weights between neurons. This penalty discourages the model from assigning overly large weights to individual connections, promoting a balanced and simplified model that focuses on the most important patterns within the data, rather than on minor, noisy details. Scout uses the α parameter to represent the strength of L2 regularization; by defaulting it to 0.01, we introduce a mild regularization effect, encouraging the model to maintain a balance between accurately fitting the training data and still complying with ground of truth databases.

In addition, the MLP-ANN incorporates an adaptive learning rate. Adaptive learning is crucial for optimizing the training process because it adjusts the rate at which the model learns from the data. This dynamic adjustment helps the model converge more efficiently and avoid getting stuck in local minima, ultimately resulting in a better trained model. Employing an adaptive learning rate can also help prevent overfitting. By adjusting the learning rate during training, the model is less likely to over-specialize on the training data. Instead, it will focus on capturing the general patterns, leading to improved generalization capabilities when encountering unseen data.

Moreover, in order to obtain optimized weights in each layer of our MLP-ANN, the model uses the log-loss function $f(x)$ in each neuron, optimized by the Limited-memory Broyden–Fletcher–Goldfarb–Shanno (LBFGS), which is an algorithm in the family of quasi-Newton methods for using a limited amount of computer memory to minimize $f(x)$ over unconstrained values of the real-vector *x* where *f* is a differentiable scalar function[3].

In conclusion, by utilizing an MLP-ANN for ranking mass spectrometry data, we can uncover and analyze complex patterns in the data, leading to more accurate and efficient results. This approach offers significant advantages over traditional linear methods, making it a valuable tool in the analysis of mass spectrometry datasets.

The hyperparameters of the MLP-ANN at all levels are:

CSM:

α = 0.01,

hidden_layer_sizes = 10,

solver = 'lbfgs',

max_iter = 1000,

learning_rate = 'adaptive',

learning_rate_init = 0.01,

n_iter_no_change = 50,

validation_fraction = 0.1

ResPair:

α = 0.01,

hidden_layer_sizes = 5,

solver = 'lbfgs',

max_iter = 1000,

learning_rate = 'adaptive',

learning_rate_init = 0.01,

n_iter_no_change = 50,

validation_fraction = 0.1

PPI:

α = 0.01,

hidden_layer_sizes = 5,

solver = 'lbfgs',

max_iter = 1000,

learning_rate = 'adaptive',

learning_rate_init = 0.01,

n_iter_no_change = 50,

validation_fraction = 0.1

SUPPLEMENTARY NOTE 5. EXTENDED DESCRIPTION OF THE BIOLOGICAL XL-MS RESULTS

We assessed Scout's suitability for analyzing biological XL-MS data based on two proteome-wide XL-MS datasets, both obtained with the MS-cleavable and enrichable cross-linker Azide-A-DSBSO. The first dataset was published by Zhu et al.[4], who cross-linked intact human mitochondria. The second dataset is a newly acquired deep XL-MS-based interactome of intact HEK293T cells. We compared Scout's performance on these datasets to results generated with MSAnnika. We chose MSAnnika because this software (1) was among the best performing in our benchmarking analysis, (2) can complete complex searches against large databases on standard desktop PCs, (3) shows one of the most robust performances when increasing the search space to larger databases, and (4) does not depend on manual post-processing with heuristic score cut-offs. The other well-performing search engines in our benchmarking analysis did not meet all of these criteria; specifically, xi did not comply with point 2 and XlinkX did not comply with points 3+4.

We used the mitochondria dataset for an entrapment experiment. To this end, we searched the data against a database comprising proteomes of human mitochondria and E.coli. In this setup, all cross-links between human proteins are considered true and cross-links involving E.coli proteins are considered false. Entrapment does not account for incorrect human-human cross-links, meaning that it is less sensitive than the FDR estimation based on our standard datasets and will likely underestimate the actual FDR. Nonetheless, it gives an indication how Scout's sensitivity and specificity compare to other search engines for real-world data. As discussed in the main text (Results sub-section "Testing Scout on biological XL-MS data"), the results of this experiment are by and large in agreement with the trends seen with our standard datasets (Figure 6A). This supports the notion that Scout is suitable for analyzing biological data.

The HEK dataset was used to put the PPI and ResPair level results of MSAnnika and Scout into an interactomic and structural context. The PPI results of both search engines were compared to the STRING interactome database as a positive control and the Negatome database of non-interacting proteins as a negative control. Compared to MSAnnika, Scout's PPI-FDR filter reduces PPI identification numbers (Figure 6B) – a trend that was also observed with our benchmarking data (Figure 3) and mitochondria data (Figure 6A). While Scout's PPI-FDR filter slightly increases the fraction of medium-to-high-confidence STRING PPIs (79% for PPI-FDR controlled Scout vs 75% for MSAnnika and 76% for Scout with PPIs aggregated from ResPair identifications), these differences are more subtle than our benchmarking results on large sequence databases would suggest (see bottom-right panel in Figure 3: 15% empirical FDR for MSAnnika vs. 2% empirical FDR for Scout on PPI level). In the absence of a ground-truth for the HEK dataset, we cannot ultimately judge to which extent these PPI identifications are inflated by false-positives. However, PPI-FDR controlled Scout also identifies the fewest PPIs from the Negatome database, supporting the view that it can reduce spurious PPI hits.

Since we repeatedly observed that Scout shows a steeper numerical drop-off from ResPair to PPI level than the search engines without dedicated PPI-FDR filter, we analyzed the relationship between ResPair and PPI level. For the HEK dataset, we find that the surplus of MSAnnika PPI identifications mainly stems from PPIs supported by a single ResPair, whereas Scout has an edge over MSAnnika in identifying PPIs supported by >6 ResPairs (Figure 6C). This indicates that Scout's PPI-FDR filter mainly removes weakly supported PPIs that are more likely to represent irreproducible "one hit wonders".

Our ResPair-level assessment involved mapping the Scout and MSAnnika identified inter-links onto AlphaFoldAF-Multimer models of the PPIs identified by these search engines. We focused on cross-links between residues with a pLDDT score >50 in order to exclude disordered protein regions. Measuring the $C\alpha$–$C\alpha$ distances of models across all confidence scores shows that Scout and MSAnnika produce data with essentially identical structural accuracy (Figure 6D); however, focusing only on models with a confidence of at least 0.5, we find that the proportion of within-distance cross-links is about 2.4% higher for MSAnnika. At the same time, Scout provides substantially more mappable cross-links (1900 vs 1481 for model confidence scores <0.5, 1824 vs 1196 for model confidence scores >0.5).

Overall, Scout provides a deeper inter-link coverage at ResPair level, which does not compromise structural accuracy in our AF2-Multimer analysis of the HEK dataset. At the same time, Scout's stringent PPI-FDR filter reduces identification sensitivity but helps remove PPIs that are more weakly supported (often only by a single ResPair inter-link), which may increase the chance that the remaining PPI hits are readily actionable for functional follow-up studies.

Supplementary Note 6. Additional considerations in the design of the analytical standard

The scale of our standard required us to design our experiments in way that would make the standard broadly usable without exceeding the available time and financial resources. To this end, we made several choices:

- All experiments were done with Orbitrap mass spectrometers because (1) they were available in our lab, (2) Orbitrap instruments were also used for acquiring the published peptide-based and quasi-ground-truth datasets[5, 6], and (3) the utility of other instruments (e.g. timsTOF platforms) for proteome-wide XL-MS is only starting to be explored[7, 8].
- All cross-linking reactions were performed with DSSO as this is one of the most widely used cross-linkers for proteome-wide XL-MS experiments. In addition, DSSO has also been used for the published peptide-based and quasi-ground-truth datasets[5, 6], which allowed us to directly compare the search engine performances on these datasets and on our benchmarking datasets.
- We used one LC-MS data acquisition strategy. We have optimized our LC-MS set-up using 8 interaction groups from our analytical standard (batch 2) that were used neither during Scout development nor for software benchmarking. We also used batch 2 to compare acquiring only MS2 scans after stepped high-energy collision-induced dissociation (HCD) of the cross-linked peptide ions (stepped-HCD-MS2) against acquiring MS2 scans followed by gas-phase fragmentation and MS3 of the product ions. When using XlinkX PD 2.5 with dynamic score filtering (since this software is compatible with both acquisition strategies), stepped-HCD-MS2 always yielded more cross-links in our hands, confirming previous findings that stepped-HCD-MS2 is the best solution for XL-MS with cleavable cross-linkers[6].

SUPPLEMENTARY TABLES

Supplementary Table 1. Protein sequence databases used for the software benchmarking with standard datasets. All .fasta files are available on PRIDE (https://www.ebi.ac.uk/pride/) under the accession code PXD052022.

Database filename	Display item	# Sequences	Origin
Scout_540.fasta	Figures 3-5<br>Supplementary Figures 1-2	255 (in standard sample)+285	HUMAN
Scout_2000.fasta	Figure 5	255+1745	HUMAN
Scout_4000.fasta	Figure 3<br>Figure 4A-C<br>Figure 5<br>Supplementary Figure 1	255+3745	HUMAN
Scout_uniprot_human_genes.fasta	Figure 5	20622	HUMAN
Matzinger_171.fasta[6]	Figure 6 A, B<br>Supplementary Table 3	171 (In standard sample)	ECOLI, HUMAN
Matzinger_671.fasta[6]	Figure 6 A, B<br>Supplementary Table 3	171+500	ECOLI, HUMAN
Matzinger_5171.fasta[6]	Figure 6 A, B<br>Supplementary Table 3	171+5000	ECOLI, HUMAN
Matzinger_20334.fasta[6]	Figure 6 A, B<br>Supplementary Table 3	171+20165	ECOLI, HUMAN
Lenz_1929.fasta[5]	Figure 6 C, D	1929	ECOLI

Supplementary Table 2. Search engine parameters used for benchmarking.

parameter	MeroX	MaxLynx	MS Annika	XlinkX PD*	xiSEARCH/xiFDR*	Scout
version	2.0.1.4	2.2.0.0	PD 2.5	PD 2.5	1.7.6.3 / 2.1.5.2	1.4.14
enzyme	Trypsin	Trypsin/P	Trypsin	Trypsin	Trypsin	Trypsin
MS1 tolerance	10 ppm	10 ppm	10 ppm	10 ppm	3 ppm	10 ppm
MS2 tolerance	20 ppm	20 ppm	20 ppm	20 ppm	6 ppm	20 ppm
minimum peptide length	6	6	6	6	6	6
peptide mass range	500 – 6,000 Da					
# missed cleavages	3	3	3	3	2	3
modifications	Oxidation of Methionine (variable)<br>Carbamidomethylation of Cysteine (static)					
FDR cutoff	1%	1%	1%	1%	1%	1%
Reaction sites	K	K	K	K	KSTY	K / KSTY#
FDR level	CSM	CSM	CSM, XL	CSM, XL	PPI (with boost)	CSM, XL, PPI
separate inter- /intralinks	on	on	on	on	on	on

* additional parameters described in the main Methods section

KSTY specificity in Scout was only used for the comparison to xi. For all other comparisons, Scout was run with K-only specificity

Supplementary Table 3. ResPair-level cross-link identifications of various search engines in two datasets derived from the synthetic peptide library by Matzinger et al.[6]. Non-cross-linked tryptic peptides from a HEK cell digest were spiked into the DSSO-cross-linked peptide main library. Samples were analyzed directly or after SEC fractionation (see original publication for details). The table is colored with a red (worst performance)-to-green (best performance) gradient by row for each database (DB) size. Data taken from Matzinger et al.[6] and Scout results added. Scout was run with identical search parameters and filtered at 1% naïve ResPair FDR.

DSSO, mainlibrary : HEK = 1 : 5

DB size	MeroX	MSAnnika	XlinkX	pLink	MaxLynx	Scout	
171	514	409	336	281	442	479	# true cross-link IDs
671	479	385	316	203	420	482	
5171	435	255	292	101	216	475	
20334	390	247	278	96	227	471	
DB size	MeroX	MSAnnika	XlinkX	pLink	MaxLynx	Scout	
171	52	7	24	12	2	6	# false cross-link IDs
671	66	16	30	11	17	4	
5171	153	12	42	4	18	14	
20334	120	18	57	10	18	13	
DB size	MeroX	MSAnnika	XlinkX	pLink	MaxLynx	Scout	
171	9.2%	1.7%	6.7%	4.1%	0.5%	1.2%	empirical FDR
671	12.1%	4.0%	8.7%	5.1%	3.9%	0.8%	
5171	26.0%	4.5%	12.6%	3.8%	7.7%	2.9%	
20334	23.5%	6.8%	17.0%	9.4%	7.3%	2.7%	

DSSO, mainlibrary : HEK = 1 : 5, SEC enriched

DB size	MeroX	MSAnnika	XlinkX	pLink	MaxLynx	Scout	
171	713	635	590	601	767	714	# true cross-link IDs
671	645	595	582	489	752	714	
5171	650	482	564	356	493	675	
20334	641	446	549	337	530	691	
DB size	MeroX	MSAnnika	XlinkX	pLink	MaxLynx	Scout	
171	47	16	81	30	26	11	# false cross-link IDs
671	216	15	117	33	50	16	
5171	118	21	104	28	26	28	
20334	230	24	134	35	30	38	
DB size	MeroX	MSAnnika	XlinkX	pLink	MaxLynx	Scout	
171	6.2%	2.5%	12.1%	4.8%	3.3%	1.5%	empirical FDR
671	25.1%	2.5%	16.7%	6.3%	6.2%	2.2%	
5171	15.4%	4.2%	15.6%	7.3%	5.0%	4.0%	
20334	26.4%	5.1%	19.6%	9.4%	5.4%	5.2%	

SUPPLEMENTARY REFERENCES

1. Clasen, M.A. et al. Increasing confidence in proteomic spectral deconvolution through mass defect. *Bioinformatics* **38**, 5119-5120 (2022).

2. Liu, F., Rijkers, D.T., Post, H. & Heck, A.J. Proteome-wide profiling of protein assemblies by cross-linking mass spectrometry. *Nat Methods* **12**, 1179-1184 (2015).

3. Dennis, J.E. & Schnabel, R.B. Numerical methods for unconstrained optimization and nonlinear equations. (Prentice-Hall, Englewood Cliffs, N.J.; 1983).

4. Zhu, Y. et al. Cross-link assisted spatial proteomics to map sub-organelle proteomes and membrane protein topologies. *Nat Commun* **15**, 3290 (2024).

5. Lenz, S. et al. Reliable identification of protein-protein interactions by crosslinking mass spectrometry. *Nat Commun* **12**, 3564 (2021).

6. Matzinger, M. et al. Mimicked synthetic ribosomal protein complex for benchmarking crosslinking mass spectrometry workflows. *Nat Commun* **13**, 3975 (2022).

7. Ihling, C.H., Piersimoni, L., Kipping, M. & Sinz, A. Cross-Linking/Mass Spectrometry Combined with Ion Mobility on a timsTOF Pro Instrument for Structural Proteomics. *Anal Chem* **93**, 11442-11450 (2021).

8. Steigenberger, B. et al. Benefits of Collisional Cross Section Assisted Precursor Selection (caps-PASEF) for Cross-linking Mass Spectrometry. *Mol Cell Proteomics* **19**, 1677-1687 (2020).
