# Supplementary material for: Proteome-scale recombinant standards and a robust high-speed search engine to advance cross-linking MS-based interactomics
Source: Nat Methods. 2024 Oct 31;21(12):2327–35. doi: 10.1038/s41592-024-02478-1 (PMC11621016; doi:10.1038/s41592-024-02478-1)
Supplement: Supplementary file 2 — Reporting Summary [file 41592_2024_2478_MOESM2_ESM.pdf]

nature portfolio

Corresponding author(s): Fan Liu, Diogo Borges Lima, Paulo C Carvalho

Last updated by author(s): Aug 1, 2024

Reporting Summary

Nature Portfolio wishes to improve the reproducibility of the work that we publish. This form provides structure for consistency and transparency in reporting. For further information on Nature Portfolio policies, see our Editorial Policies and the Editorial Policy Checklist.

Statistics

For all statistical analyses, confirm that the following items are present in the figure legend, table legend, main text, or Methods section.

n/a	Confirmed	
☐	☒	The exact sample size (*n*) for each experimental group/condition, given as a discrete number and unit of measurement
☐	☒	A statement on whether measurements were taken from distinct samples or whether the same sample was measured repeatedly
☒	☐	The statistical test(s) used AND whether they are one- or two-sided<br>*Only common tests should be described solely by name; describe more complex techniques in the Methods section.*
☒	☐	A description of all covariates tested
☐	☒	A description of any assumptions or corrections, such as tests of normality and adjustment for multiple comparisons
☐	☒	A full description of the statistical parameters including central tendency (e.g. means) or other basic estimates (e.g. regression coefficient) AND variation (e.g. standard deviation) or associated estimates of uncertainty (e.g. confidence intervals)
☒	☐	For null hypothesis testing, the test statistic (e.g. *F*, *t*, *r*) with confidence intervals, effect sizes, degrees of freedom and *P* value noted<br>*Give P values as exact values whenever suitable.*
☒	☐	For Bayesian analysis, information on the choice of priors and Markov chain Monte Carlo settings
☒	☐	For hierarchical and complex designs, identification of the appropriate level for tests and full reporting of outcomes
☒	☐	Estimates of effect sizes (e.g. Cohen's *d*, Pearson's *r*), indicating how they were calculated

Our web collection on statistics for biologists contains articles on many of the points above.

Software and code

Policy information about availability of computer code

Data collection	Orbitrap Fusion Lumos Instrument Control Software version 3.4 (Thermo Scientific)
Data analysis	Scout v1.4.14 (source code, software and user documentation available at https://github.com/theliulab/Scout). Data were also searched with xiSEARCH/xiFDR v2.1.5.2, MaxLynx, MSAnnika, XlinkX in Proteome Discoverer v2.5 (Thermo Scientific), and MeroX - references to the original publications are provided in the main manuscript.<br><br>R v.4.4, AlphaFold-Multimer 2.3, ColabFold (Mirdita, M. et al. ColabFold: making protein folding accessible to all. Nat Methods 19, 679-682, 2022)

For manuscripts utilizing custom algorithms or software that are central to the research but not yet described in published literature, software must be made available to editors and reviewers. We strongly encourage code deposition in a community repository (e.g. GitHub). See the Nature Portfolio guidelines for submitting code & software for further information.

Data

Policy information about availability of data

All manuscripts must include a data availability statement. This statement should provide the following information, where applicable:

- Accession codes, unique identifiers, or web links for publicly available datasets
- A description of any restrictions on data availability
- For clinical datasets or third party data, please ensure that the statement adheres to our policy

TThe mass spectrometry proteomics data have been deposited to the ProteomeXchange Consortium via the PRIDE partner repository:
- The HEK cell XL-MS raw data are available with the dataset identifier PXD043531
- The ground-truth raw data used in this manuscript and the corresponding result files underlying the software comparison are available under the dataset identifier PXD052022
- The full collection of raw datasets obtained from the recombinant XL-MS standard, the concept of which is reported here, are available as a full submission with the dataset identifier PXD042173

The published datasets re-analyzed in this study are available through ProteomeXchange with dataset identifiers PXD019120 (fractionated E.coli lysate standard dataset from Lenz et al.12), PXD029252 (synthetic peptide standard dataset from Matzinger et al.10), and PXD046382 (mitochondria XL-MS dataset from Zhu et al.25).

Research involving human participants, their data, or biological material

Policy information about studies with human participants or human data. See also policy information about sex, gender (identity/presentation), and sexual orientation and race, ethnicity and racism.

Reporting on sex and gender	n/a
Reporting on race, ethnicity, or other socially relevant groupings	n/a
Population characteristics	n/a
Recruitment	n/a
Ethics oversight	n/a

Note that full information on the approval of the study protocol must also be provided in the manuscript.

Field-specific reporting

Please select the one below that is the best fit for your research. If you are not sure, read the appropriate sections before making your selection.

☒ Life sciences ☐ Behavioural & social sciences ☐ Ecological, evolutionary & environmental sciences

For a reference copy of the document with all sections, see nature.com/documents/nr-reporting-summary-flat.pdf

Life sciences study design

All studies must disclose on these points even when the disclosure is negative.

Sample size	The size of the recombinant protein standard was chosen such that a MS2-level complexity comparable to published proteome-wide XL-MS studies could be achieved. Samples size for the HEK cell XL-MS experiment was chosen based on preliminary experiments and common practice in the field; without any statistical sample size calculation.
Data exclusions	No data were excluded from our analyses.
Replication	Replication is not directly relevant because the focus of this work was to obtain a large-scale ground truth dataset based on a newly developed recombinant XL-MS standard. We used this ground-truth data to assess the reproducibility and robustness of existing XL-MS search engines and develop the Scout search engine. The reliability of Scout was independently validated using a previously published small-scale ground-truth dataset, showing that the vast majority of Scout identifications agree with the identifications of the best-performing software in the previous publication (Matzinger et al., Nat Commun, 2022). Replication is also not directly relevant to the biological XL-MS data (re-)analyzed in this study because they were only used to confirm the functionality and performance of published XL-S search engines and Scout.
Randomization	The proteins in our recombinant XL-MS standard were randomly allocated to interaction groups, each containing 8 proteins. The interaction groups were randomly split into batches, each consisting of 8 interaction groups. Randomization was not relevant to the other experiments because they did not involve any group allocation.

Blinding	Not relevant to this study. In order to generate a ground-truth dataset, investigators needed to know which proteins are in which interaction group.

Reporting for specific materials, systems and methods

We require information from authors about some types of materials, experimental systems and methods used in many studies. Here, indicate whether each material, system or method listed is relevant to your study. If you are not sure if a list item applies to your research, read the appropriate section before selecting a response.

Materials & experimental systems

n/a	Involved in the study	
☒	☐	Antibodies
☐	☒	Eukaryotic cell lines
☒	☐	Palaeontology and archaeology
☒	☐	Animals and other organisms
☒	☐	Clinical data
☒	☐	Dual use research of concern
☒	☐	Plants

Methods

n/a	Involved in the study	
☒	☐	ChIP-seq
☒	☐	Flow cytometry
☒	☐	MRI-based neuroimaging

Eukaryotic cell lines

Policy information about cell lines and Sex and Gender in Research

Cell line source(s)	HEK293T (CRL-3216, ATCC)
Authentication	Cell line was not further authenticated.
Mycoplasma contamination	Cell lines was tested negative for mycoplasma contamination.
Commonly misidentified lines (See ICLAC register)	No commonly misidentified lines were used in this study.
