# Supplementary material for: Proteome-scale recombinant standards and a robust high-speed search engine to advance cross-linking MS-based interactomics
Source: Nat Methods. 2024 Oct 31;21(12):2327–35. doi: 10.1038/s41592-024-02478-1 (PMC11621016; doi:10.1038/s41592-024-02478-1)
Supplement: Supplementary file 4 — Amino acid sequence and SDS–PAGE of all proteins included in the recombinant XL-MS standard. [file 41592_2024_2478_MOESM4_ESM.pdf]

Uniprot ID	Gene name	start	stop	Sequence	SDS-PAGE	group
P49591	SARS1	1	514	MVLDLDLFRVDKGGDPALIRETQEKRFKDPGLVDQLVKA<br>DSEWRRCRFRADNLNKLKNLCSKTIGEKMKKKEPVGDDE<br>SVPENVLSFDDLTADALANLKVSQIKKVRLLIDEAILKCDA<br>ERIKLEAERFENLREIGNLLHPSVPISNDEDVDNKVERIWG<br>DCTVRKKYSHVDLVVMVDGFEGEKGAVVAGSRGYFLKG<br>VLVFLEQALIQYALRTLGSRGYIPIYTPFFMRKEVMQEVAQ<br>LSQFDEELYKVIGKGSEKSDDNSYDEKYLIATSEQPIAALH<br>RDEWLRPEDLPIKYAGLSTCFRQEVGSHGRDTRGIFRVH<br>QFEKIEQFVYSSPHDNKSWEMFEEMITTAEEFYQSLGIPY<br>HIVNIVSGSLNHAASKKLDLEAWFPGSGAFRELVSCSNCT<br>DYQARRLRIRYGQTKKMMDKVEFVHMLNATMCATTRTI<br>CAILENYQTEKGITVPEKLKEFMPPGLQELIPFVKPAPIEQE<br>PSKKQKKQHEGSKKKAAARDVTLENRLQNMEVTDALEH<br>HHHHH	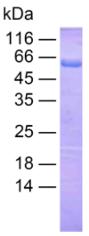	Group1<br>_batch1
P08912	CHRM5	215	443	MRIYRETEKRTKDLADLQGSDSVTKAEKRKPAHRALFRSC<br>LRCPRPTLAQRERNQASWSSSRRSTSTTGKPSQATGPSA<br>NWAKAEQLTTCSSYPSSEDEDKPATDPVLQVVYKSQGKE<br>SPGEEFSAEETEETFVKAETEKSDYDTPNYLLSPAAAHRPK<br>SQKCVAYKFRLVVKADGNQETNNGCHKVKIMPCPFPVA<br>KEPSTKGLNPNPSHQMTKRKRVVLVKERKAAQTLEHHH<br>HHH	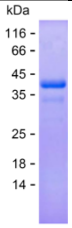	Group1<br>_batch1
Q96NL8	CFAP418	1	207	MAEDLDELLDEVESKFCTPDLLRRGMVEQPKGCGGGTH<br>SSDRNQAKAKETLRSTETFKKEDDLDSLINEILEEPNLDKK<br>PSKLKSKSSGNTSVRASIEGLGKSCSPVYLGGSSIPCGIGT<br>NISWRACDHLRCIACDFLVVSYDDYMWDKSCDYLFFRN<br>NMPEFHKLKAKLIKKKGTRAYACQCSWRTIEEVTDLQTD<br>HQLRWVCGKHLEHHHHHH	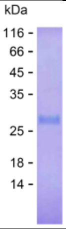	Group1<br>_batch1
P08172	CHRM2	210	387	MSRASKSRIKKDKKEPVANQDPVSPSLVQGRIVKPNNNN<br>MPSSDDGLEHNKIQNGKAPRDPVTENCVQGEEKESSND<br>STSVSAVASNMRDDEITQDENTVSTSLGHSKDENSKQTC<br>IRIGTKTPKSDSCTPTNTTVEVVGSSGQNGDEKQNIVARK<br>IVKMTKQPAKKKPPPSREKKVTRLEHHHHHH	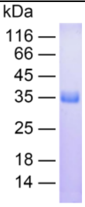	Group1<br>_batch1
P49903	SEPHS1	1	392	MSTRESFNPESYELDKSFRLTRFTELKGTGCKVPQDVLQK<br>LLESLQENHFQEDEQFLGAVMPRLGIGMDTCVIPLRHGG<br>LSLVQTTDYIYPIVDDPYMMGRIACANVLSDLYAMGVTE<br>CDNMLMLLGVSNKMTDRERDKVMPLIIQGFKDAAEEAG<br>TSVTGGQTVLNPWIVLGGVATTVCQPNEFIMPDNAVPG<br>DVLVLTKPLGTQVAVAVHQWLDIPEKWNKIKLVVTQED<br>VELAYQEAMMNMARLNRTAAGLMHTFNAHAATDITGF<br>GILGHAQNLAKQQRNEVSFVIHNLPVLAKMAAVSKACG<br>NMFGLMHGTCPETSGGLLICLPREQAARFCAEIKSPKYGE<br>GHQAWIIGIVEKGNRTARIIDKPRIIEVAPQVATQNVNPT<br>PGATSLEHHHHHH	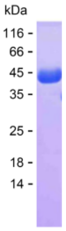	Group1<br>_batch1
P11309	PIM1	1	313	MLLSKINSLAHLRAAPCNDLHATKLAPGKEKEPLESQYQV<br>GPLLGSGGFGSVYSGIRVSDNLPVAIKHVEKDRISDWGEL<br>PNGTRVPMEVVLLKKVSSGFSGVIRLLDWFERPDSFVLILE<br>RPEPVQDLFDFITERGALQEELARSFFWQVLEAVRHCHN<br>CGVLHRDIKDENILIDLNRGELKLIDFGSGALLKDTVYTDF<br>DGTRVYSPPEWIRYHRYHGRSAAVWSLGILLYDMVCGDI<br>PFEHDEEIIRGQVFFRQRVSSECQHLIRWCLALRPSDRPTF<br>EEIQNHPWMQDVLLPQETAEIHLHSLSPGPSKLEHHHH<br>HH	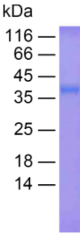	Group1<br>_batch1

P0C842	LINC00614	1	121	MEGRNCTVEILPERLNIEGWYDADDTKPGKSWAGRAASIERLNEFDNNLFGISDLEAECLDPQQKLLLECTYGALESAGVPAKEVAGSRTGVFIGIMNQDYEFMSRRTPRMQTTVMPLDLQLEHHHHHH	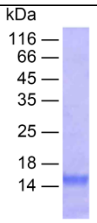	Group1 _batch4
O14950	MYL12B	1	172	MSSKKAKTKTTKKRPQRATSNVFAMFDQSQIQEFKEAFNMIDQNRDGFIDKEDLHDMLASLGKNPTDAYLDAMMNEAPGPINFTMFLTMFGEKLNGTDPEDVIRNAFACFDEEATGTIQEDYLRELLTTMGDRFTDEEVDELYREAPIDKKGNFNYIEFTRILKHGAKDKDDLEHHHHHH	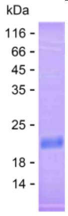	Group1 _batch1
P49069	CAMLG	1	189	MESMAVATDGGERPGVPAGSGLSASQRRAELRRRKLLMNSEQRINRIMGFHRPGSGAEEESQTKSKQQDSDKLNSLSVPSVSKRVVLGDSVSTGTTDQQGGVAEVKGTQLGDKLDSFIKPPECSSDVNLELRQRNRGDLTADSVQRGSRHGLEQYLSRFEEAMKLRKQLISEKPSQEDGNTTEEFDSFRLEHHHHHH	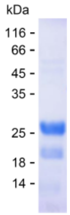	Group2 _batch1
P06746	POLB	1	335	MSKRKAPQETLNGGITDMLTELANFEKNVSQAIHKYNAYRKAASVIAKYPHKIKSGAEAKKLPGVGTKIAEKIDEFLATGKLRKLEKIRQDDTSSSINFLTRVSGIGPSAARKFVDEGIKTLEDLRKNEDKLNHHQRIGLKYFGDFEKRIPREEMLQMQDIVLNEVKKVDSEYIATVCGSFRRGAESSGDMDVLLTHPSFTSESTKQPKLLHQVVEQLQKVHFITDTLSKGETKFMGVCQLPSKNDEKEYPHRRIDIRLIPKDQYYCGVLYFTGSDIFNKNMRAHALEKGFTINEYTIRPLGVTGVAGEPLPVDSEKDIFDYIQWKYREPKDRSELEHHHHHH	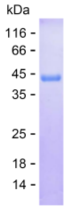	Group2 _batch1
O75575	CRCP	1	148	MEVKDANSALLSNYEVFQLLTDLKEQRKESGKNKHSSGQQNLNTITYETLKYISKTPCRHQSPEIVREFLTALKSHKLTKAEKLQLLNHRPVTAVEIQLMVEESEERLTEEQIEALLHTVTSILPAEPEAEQKKNTNSNVAMDEEDPALEHHHHHH	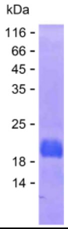	Group2 _batch1
Q8TEA8	DTD1	1	209	MKAVVQRVTRASVTVGGEQISAIGRGICVLLGISLEDTQKELEHMVRKILNLRVFEDESGKHWSKSVMDKQYEILCVSQFTLQCVLKGNKPDFHLAMPTEQAEGFYNSFLEQLRKTYRPELIKDGKFGAYMQVHIQNDGPVTIELESPAPGTATSDPKQLSKLEKQQQRKEKTRAKGPSESSKERNTPRKEDRSASSGAEGDVSSEREPLEHHHHHH	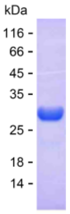	Group2 _batch1
Q8N9N5	BANP	1	519	MMSEHDLADVVQIAVEDLSPDHPVVLENHVVTDEDEPALKRQRLEINCQDPSIKTICLRLDSIEAKLQALEATCKSLEEKLDLVTNKQHSPIQVPMVAGSPLGATQTCNKVRCVVPQTTVILNNDRQNAIVAKMEDPLSNRAPDSLENVISNAVPGRRQNTIVVKVPGQEDSHHEDGESGSEASDSVSSCGQAGSQSIGSNVTLITLNSEEDYPNGTWLGDENNPEMRVRCAIIPSDMLHISTNCRTAEKMALTLLDYLFHREVQAVSNLSGQGKHGKKQLDPLTIYGIRCHLFYKFGITESDWYRIKQSIDSKCRTAWRRKQRGQSLAVKSFSRRTPNSSSYCPSEPMMSTPPPASELPQPQPQPQALHYALANAQQVQIHQIGEDGQVQVGHLHIAQVPQGEQVQITQDSEGNLQIHHVGQDGQLLEATRIPCLLAPSVFKASSGQVLQGAQLIAVASSDPAAAGVDGSPLQGSDIQVQYVQLAPVSDHTAGAQTAEALQPTLQPEMQLEHGAIQIQLEHHHHHH	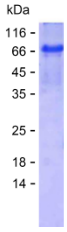	Group2 _batch1

P0C024	NUDT7	1	238	MSRLGLPEEPVRNSLLDDAKARLRKYDIGGKYSHLPYNKYSVLLPLVAKEGKLHLLFTVRSEKLRRAPGEVCFPGGKRDPTDMDDAATALREAQEEVGLRPHQVEVVCCLVPCLIDTDTLITPFVGLIDHNFQAQPNPAEVKDVFLVPLAYFLHPQVHDQHYVTRLGHRFINHIFEYTNPEDGVTYQIKGMTANLAVLVAFIILEKKPTFEVQFNLNDVLASSEELFLKVHKKATSRLEHHHHHH	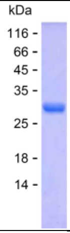	Group2 _batch1
O60356	NUPR1	1	82	MATFPPATSAPQQPPGPEDEDSSLDESDLYSLAHSYLGGGGRKGRTKREAAANTNRPSPGGHERKLVTKLQNSERKKRGARRLEHHHHHH	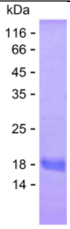	Group2 _batch1
Q9NRX4	PHPT1	1	125	MAVADLALIPDVDIDSDGVFKYVLIRVHSAPRSGAPAAESKEIVRGYKWAEYHADIYDKVSGDMQKQGCDCECLGGGRISHQSQDKKIHVYGYSMAYGPAQHAISTEKIKAKYPDYEVTWANDGYLEHHHHHH	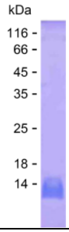	Group2 _batch1
Q92572	AP3S1	1	193	MIKAILIFNNHGKPRLSKFYQPYSEDTQQQIIRETFHLVSKRDENVCNFLEGGLLIGGSDNKLIYRHYATLYFVFCVDSSESELGILDLIQVFVETLDKCFENVCELDLIFHVDKVHNILAEMVMGGMVLETNMNEIVTQIDAQNKLEKSEAGLAGAPARAVSAVKNMNLPEIPRNINIGDISIKVPNLPSFKLEHHHHHH	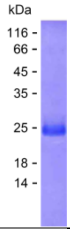	Group3 _batch1
Q96GE6	CALML4	1	196	MAAEHLLPGPPPSLADFRLEAGGKGTERGSGSSKPTGSSRGPRMAKFLSQDQINEYKECFSLYDKQQRGKIKATDLMVAMRCLGASPTPGEVQRHLQTHGIDGNGELDFSTFLTIMHMQIKQEDPKKEILLAMLMVDKEKKGYVMASDLRSKLTSLGEKLTHKEVDDLFREADIEPNGKVKYDEFIHKITLPGRDYLEHHHHHH	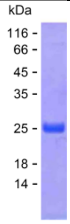	Group3 _batch1
O43504	LAMTOR5	1	91	MEATLEQHLEDTMKNPSIVGVLCTDSQGLNLGCRGTLSDEHAGVISVLAQQAAKLTSDPTDIPVVCLESDNGNIMIQKHDGITVAVHKMASLEHHHHHH	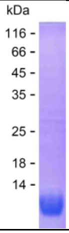	Group3 _batch1
P31415	CASQ1	35	396	MQEGLDFPEYDGVDRVINVNAKNYKNVFKKYEVLALLYHEPPEDDKASQRQFEMEELILELAAQVLEDKGVGFGLVDSEKDAAVAKKLGLTEVDSMYVFKGDEVIEYDGEFSADTIVEFLLDVLEDPVELIEGERELQAFENIEDEIKLIGYFKSKDSEHYKAFEDAAEEFHPYIPFFATFDSKVAKKLTLKLNEIDFYEAFMEEPVTIPDKPNSEEEIVNFVEEHRRSTLRKLKPESMYETWEDDMDGIHIVAFAEEADPDGFEFLETLKAVAQDNTENPDLSIIWIDPDDFPLLVPYWEKTFDIDLSAPQIGVVNVTDADSVWMEMDDEEDLPSAEELEDWLEDVLEGEINTEDDDDDDDDLEHHHHHH	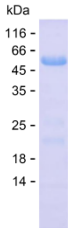	Group3 _batch1
Q15286	RAB35	1	201	MARDYDHLFKLLIIGDSGVGKSSLLLRFADNTFSGSYITTIGVDFKIRTVEINGEKVKLQIWDTAGQERFRTITSTYYRGTHGVIVVYDVTSAESFVNVKRWLHEINQNCDDVCRILVGNKNDDPERKVVETEDAYKFAGQMGIQLFETSAKENVNVEEMFNCITELVLRAKKDNLAKQQQQQQNDVVKLTKNSKRKKRCCLEHHHHHH	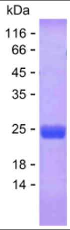	Group3 _batch1

P09972	ALDOC	1	364	MPHSYPALSAEQKKELSDIALRIVAPGKGILAADESVGSMAKRLSQIGVENTEENRRLYRQVLFSADDRVKKCIGGVIFFHETLYQKDDNGVPFVRTIQDKGIVVGIKVDKGVVPLAGTDGETTTQGLDGLSERCAQYKKDGADFAKWRCVLKISERTPSALAILENANVLARYASICQQNGIVPIVEPEILPDGDHDLKRCQYVTEKVLAAVYKALSDHHVYLEGTLLKPNMVTPGHACPIKYTPEEIAMATVTALRRTVPPAVPGVTFLSGGQSEEEASFNLNAINRCPLPRPWALTFSYGRALQASALNAWRGQRDNAGAATEEFIKRAEVNGLAAQGKYEGSGEDGGAAAQSLYIANHAYLEHHHHHH	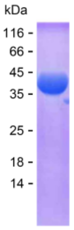	Group3_batch1
O75874	IDH1	1	414	MSKKISGGSVVEMQGDEMTRIIWELIKEKLIFPYVELDLHSYDLGIENRDATNDQVTKDAAEAIKKHNVGVKCATITPDEKRVEEFKLKQMWKSPNGTIRNILGGTVFREAIICKNIPRLVSGWVKPIIIGRHAYGDQYRATDFVVPGPGKVEITYTPSDGTQKVTYLVHNFEEGGGVAMGMYNQDKSIEDFAHSSFQMALSKGWPLYLSTKNTILKKYDGRFKDIFQEIYDKQYKSQFEAQKIWYEHRLIDDMVAQAMKSEGGFIWACKNYDGDVQSDSVAQGYGSLGMMTSVLVCPDGKTVEAEAAHGTVTRHYRMYQKGQETSTNPIASIFAWTRGLAHRAKLDNNKELAFFANALEEVSIETIEAGFMTKDLAACIKGLPNVQRSDYLNTFEFMDKLGENLKIKLAQAKLEHHHHHH	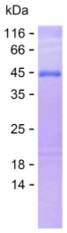	Group3_batch1
Q9H246	C1orf21	1	121	MGCASAKHVATVQNEEEAQKGKNYQNGDVFGDEYRIKPVEEVKYMKNGAEEEQKIAARNQENLEKSASSNVRLKTNKEVPGLVHQPRANMHISESQQEFFRMLDEKIEKGRDYCSEEEDITLEHHHHHH	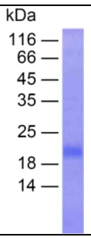	Group3_batch1
P49675	STAR	64	285	MEETLYSDQELAYLQQGEEAMQKALGILSNQEGWKKESQQDNGDKVMSKVVPDVGKVFRLEVVVDQPMERLYEELVERMEAMGEWNPNVKEIKVLQKIGKDTFITHELAAEAAGNLVGPRDFVSVRCAKRRGSTCVLAGMATDFGNMPEQKGVIRAEHGPTCMVLHPLAGSPSKTKLTWLLSIDLKGWLPKSIINQVLSQTQVDFANHLRKRLESHPASEARCLEHHHHHH	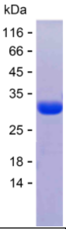	Group4_batch1
P00492	HPRT1	11	210	SDDEPGYDLDLFCIPNHYAEDLERVFIPHGLIMDRTERLARDVMKEMGGHHIVALCVLKGGYKFFADLLDYIKALNRNSDRSIPMTVDFIRLKSYCNDQSTGDIKVIGGDDLSTLTGKNVLIVEDIIDTGKTMQTLLSLVRQYNPKMVKVASLLVKRTPRSVGYKPDFVGFEIPDKFVVGYALDYNEYFRDLNHVCVISE	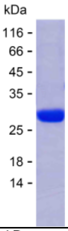	Group4_batch1
O15305	PMM2	1	218	MATRSPGVVISDDEPGYDLDLFCIPNHYAEDLERVFIPHGLIMDRTERLARDVMKEMGGHHIVALCVLKGGYKFFADLLDYIKALNRNSDRSIPMTVDFIRLKSYCNDQSTGDIKVIGGDDLSTLTGKNVLIVEDIIDTGKTMQTLLSLVRQYNPKMVKVASLLVKRTPRSVGYKPDFVGFEIPDKFVVGYALDYNEYFRDLNHVCVISETGKAKYKALEHHHHHH	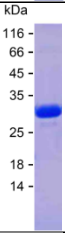	Group4_batch1
Q13765	NACA	1	215	MPGEATETVPATEQELPQPQAETGSGTESDSDESVPELEEQDSTQATTQQAQLAAAAEIDEEPVSKAKQSRSEKKARKAMSKLGLRQVTGVTRVTIRKSKNILFVITKPDVYKSPASDTYIVFGEAKIEDLSQQAQLAAAEKFKVQGEAVSNIQENTQTPTVQEESEEEEVDETGVEVKDIELVMSQANVSRAKAVRALKNNSNDIVNAIMELTMLEHHHHHH	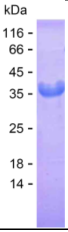	Group4_batch1

O95372	LYPLA2	1	231	MCGNTMSVPLLTDAATVSGAERETAAVIFLHGLGDTGHSWADALSTIRLPHVKYICPHAPRIPVTLNMKMVMPSWFDLMGLSPDAPEDEAGIKKAAENIKALIEHEMKNGIPANRIVLGGFSQGGALSLYTALTCPHPLAGIVALSCWLPLHRAFPQAANGSAKDLAILQCHGELDPMVPVRFGALTAEKLRSVVTPARVQFKTYPGVMHSSCPQEMAAVKEFLEKLLPPVLEHHHHH	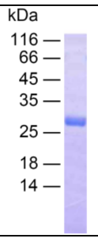	Group4_batch1
Q99986	VRK1	1	396	MPRVKAAQAGRQSSAKRHLAEQFAVGEIITDMAKKEWKVGLPIGQGGFGCIYLADMNSSESVGSDAPCVVKVEPSDNGPLFTELKFYQRAAKPEQIQKWIRTRKLKYLGVPKYWGSGLHDKNGKSYRFMIMDRFGSDLQKIYEANAKRFSRKTVLQLSLRILDILEYIHEHEYVHGDIKASNLLLNYKNPDQVYLVDYGLAYRYCPEGVHKEYKEDPKRCHDGTIEFTSIDAHNGVAPSRRGDLEILGYCMIQWLTGHLPWEDNLKDPKYVRDSKIRYRENIASLMDKCFPEKNKPGEIAKYMETVKLLDYTEKPLYENLRDILLQGLKAIGSKDDGKLDLSVVENGGLKAKTITKKRKKEIEESKEPGVEDTEWSNTQTEEAIQTRSRTRKRVQKLEHHHHHH	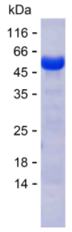	Group4_batch1
P30049	ATP5F1D	23	168	MAEAAAAPAAASGPNQMSFTFASPTQVFFNGANVRQVDVPTLTGAFGILAAHVPTLQVLRPGLVVVHAEDGTTSKYFVSSGSIAVNADSSVQLLAEEAVTLDMLDLGAAKANLEKAQAELVGTADEATRAEIQIRIEANEALVKALEHHHHHH	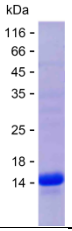	Group4_batch1
P20618	PSMB1	29	241	MRFSPYVFNGGTILAIAGEDFAIVASDTRLSEGFSIHTRDSPKCYKLTDKTVIGCSGFHGDCLTLTKIIEARLKMYKHSNNKAMTTGAIAAMLSTILYSRRFFPYYVYNIIGGLDEEGKGAVYSFDPVGSYQRDSFKAGGSASAMLQPLLDNQVGFKNMQNVEHVPLSLDRAMRLVKDVFISAAERDVYTGDALRICIVTKEGIREETVSLRKDLEHHHHHH	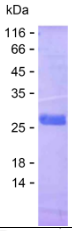	Group4_batch1
P08173	CHRM4	217	401	MHISLASRSRVHKHRPEGPKEKKAKTLAFLKSPLMKQSVKKPPPGEAAREELRNGKLEEAPPPALPPPPRPVADKDTSNESSSGSATQNTKERPATELSTTEATTPAMPAPPLQPRALNPASRWSKIQIVTKQTGNECVTAIEIVPATPAGMRPAANVARKFASIARNQVRKKRQMAARERKVTRTLEHHHHHH	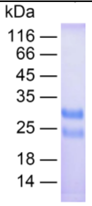	Group5_batch1
P11926	ODC1	1	461	MNNFGNEEFDCHFLDEGFTAKDILDQKINEVSSSDDKDAFYVADLGDILKKHLRWLKALPRVTPFYAVKCNDSKAIVKTLAATGTGFDCASKTEIQLVQSLGVPPERIIYANPCKQVSQIKYAANNGVQMMTFDSEVELMKVARAHPKAKLVLRIATDDSKAVCRLSVKFGATLRTSRLLLERAKELNIDVVGVSFHVGSGCTDPETFVQAISDARCVFDMGAEVGFSMYLLDIGGGFPGSEDVKLKFEEITGVINPALDKYFPSDSGVRIIAEPGRYYVASAFTLAVNIIAKKIVLKEQTGSDDEDESSEQTFMYYVNDGVYGSFNCILYDHAHVKPLLQKRPKPDEKYYSSSIWGPTCDGLDRIVERCDLPEMHVGDWMLFENMGAYTVAAASTFNGFQRPTIYYVMSGPAWQLMQQFQNPDFPPEVEEQDASTLPVSCAWESGMKRHRAACASASINVLEHHHHHH	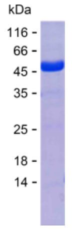	Group5_batch1
P45954	ACADSB	34	432	MKSSQSEALLNITNNGIHFAPLQTFTDEEMMIKSSVKKFAQEQIAPLVSTMDENSKMEKSVIQGLFQQGLMGIEVDPEYGGTGASFLSTVLVIEELAKVDASVAVFCEIQNTLINTLIRKHGTEEQKATYLPQLTTEKVGSFCLSEAGAGSDSFALKTRADKEGDYYVLNGSKMWISSAEHAGLFLVMANVDPTIGYKGITSFLVDRDTPGLHIGKPENKLGLRASSTCPLTFENVKVPEANILGQIGHGYKYAIGSLNEGRIGIAAQMLGLAQGCFDYTIPYIKERIQFGKRLFDFQGLQHQVAHVATQLEAARLLTYN	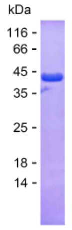	Group5_batch1

				AARLLEAGKPFIKEASMAKYYASEIAGQTTSKCIEWMGGV<br>GYTKDYPVEKYFRDAKIGTIYEGASNIQLNTIAKHIDAEYLE<br>HHHHHH		
Q9GZZ1	NAA50	1	169	MKGSRIELGDVTPHNIKQLKRLNQVIFPVSYNDKFYKDVL<br>EVGELAKLAYFNDIAVGAVCCRVDHSQNQKRLYIMTLGC<br>LAPYRRLGIGTKMLNHVLNICEKDGTFDNIYLHVQISNES<br>AIDFYRKFGFEIIETKKNYYKRIEPADAHVLQKNLKVPSGQ<br>NADVQKTDNLEHHHHHH	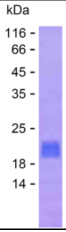	Group5<br>_batch1
P60891	PRPS1	1	318	MPNIKIFSGSSHQDLSQKIADRLGLELGKVVTKKFSNQET<br>CVEIGESVRGEDVYIVQSGCGEINDNLMELLIMINACKIAS<br>ASRVTAVIPCFPYARQDKKDKSRAPISAKLVANMLSVAG<br>ADHIITMDLHASQIQGFFDIPVDNLYAEPAVLKWIRENISE<br>WRNCTIVSPDAGGAKRVTSIADRLNVDFALIHKERKKAN<br>EVDRMVLVGDVKDRVAILVDDMADTCGTICHAADKLLS<br>AGATRVYAILTHGIFSGPAISRINNACFEAVVVTNTIPQED<br>KMKHCSKIQVIDISMILAEAIRRTHNGESVSYLFSHVPLEH<br>HHHHH	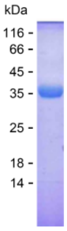	Group5<br>_batch1
Q8NCU1	CCDC197	1	143	MAAMDTGQRADPSNPGDKEGDLQGLWQELYQLQAK<br>QKKLKREVEKHKLFEDYLIKVLEKIPEGCTGWEEPEEVLVEA<br>TVKHYGKLFTASQDTQKRLEAFCQMIQAVHRSLESLEED<br>HRALIASRSGCVSCRRSATASRSSGGSLEHHHHHH	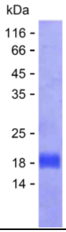	Group5<br>_batch1
O76054	SEC14L2	1	403	MSGRVGDLSPRQKEALAKFRENVQDVLPALPNPDDYFLL<br>RWLRARSFDLQKSEAMLRKHVEFRKQKDIDNIISWQPPE<br>VIQQYLSGGMCGYDLDGCPVWYDIIGPLDAKGLLFSASK<br>QDLLRTKMRECELLLQECAHQTTKLGRKVETITIIYDCEGL<br>GLKHLWKPAVEAYGEFLCMFEENYPETLKRLFVVKAPKLF<br>PVAYNLIKPFLSEDTRKKIMVLGANWKEVLLKHISPDQVP<br>VEYGGTMTDPDGNPKCKSKINYGGDIPRKYYVRDQVKQ<br>QYEHSVQISRGSSHQVEYEILFPGCVLRWQFMSDGADV<br>GFGIFLKTKMGERQRAGEMTEVLPNQRYNSHLVPEDGTL<br>TCSDPGIYVLRFDNTYSFIHAKKVNFTVEVLLPDKASEEKM<br>KQLGAGTPKLEHHHHHH	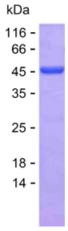	Group5<br>_batch1
P52565	ARHGDIA	1	204	MAEQEPTAEQLAQIAAENEEDEHSVNYKPPAQKSIQEIQ<br>ELDKDDESLRKYKEALLGRVAVSADPNVPNVVVTGLTLV<br>CSSAPGPLELDLTGDLESFKKQSFVLKEGVEYRIKISFRVNR<br>EIVSGMKYIQHTYRKGVKIDKTDYMVGSYGPRAEEYEFLT<br>PVEEAPKGMLARGSYSIKSRFTDDDKTDHLSWEWNLTIK<br>KDWKDLEHHHHHH	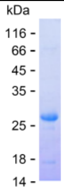	Group5<br>_batch1
Q9NPB3	CABP2	1	220	MGNCAKRPWRRGPKDPLQWLGSPPRGSCPSPSSSPKE<br>QGDPAPGVQGYSVLNSLVGPACIFLRPSIAATQLDRELRP<br>EEIEELQVAFQEFDRDRDGYIGCRELGACMRTLGYMPTE<br>MELIEISQQISGGKVDFEDFVELMGPKLLAETADMIGVREL<br>RDAFREFDTNGDGRISVGELRAALKALLGERLSQREVDEIL<br>QDVDLNGDGLVDFEEFVRMMSRLEHHHHHH	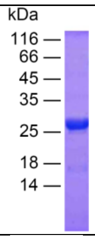	Group6<br>_batch1
P43359	MAGEA5P	1	124	MSLEQKSQHCKPEEGLDTQEEALGLVGVQAATTEEQEA<br>VSSSSPLVPGTLGEVPAAGSPGPLKSPQGASAIPTAIDFTL<br>WRQSIKGSSNQEEEGPSTSPDPESVFRAALSKKVADLIHF<br>LLLKYLEHHHHHH	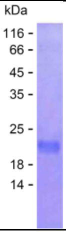	Group6<br>_batch1

P50502	ST13	1	369	MDPRKVNELRAFVKMCKQDPSVLHTEEMRFLREWVESMGGKVPPATQKAKSEENTKEEKPDSKKVEEDLKADEPSSEESDLEIDKEGVIEPDTDAPQEMGDENAEITEEMMDQANDKKVAAIEALNDGELQKAIDLFTDAIKLNPRLAILYAKRASVFVKLQKPNAAIRDCDRAIEINPDSAQPYKWRGKAHRLLGHWEEAAHDLALACKLDYDEDASAMLKEVQPRAQKIAEHRRKYERKREEREIKERIERVKKAREEHERAQREEEARRQSGAQYGSFPGGFPGGMPGNFPGGMPGMGGGMPGMAGMPGLNEILSDPEVLAAMQDPEVMVAFQDVAQNPANMSKYQSNPKVMNLISKLSAKFGGQALEHHHHHH	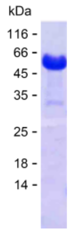	Group6<br>_batch1
P21695	GPD1	1	349	MASKKVCIVGSGNWGSAIAKIVGGNAAQLAQFDPRVTMWVFEEDIGGKKLTEIINTQHENVKYLPGHKLPPNVVAVPDVVQAAEDADILIFVVPHQFIGKICDQLKGHLKANATGISLIKGVDEGPNGLKLISEVIGERLGIPMSVLMGANIASEVADEKFCETTIGCKDPAQGQLLKELMQTPNFRITVVQEVDTVEICGALKNVVAVGAGFCDGLGFGDNTKAAVIRLGLMEMIAFAKLFCSGPVSSATFLESCGVADLITTCYGGRNRKVAEAFARTGKSIEQLEKELLNGQKLQGPETARELYSILQHKGLVDKFPLFMAVYKVCYEGQPVGEFIHCLQNHPEHMLEHHHHH	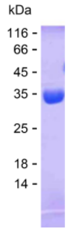	Group6<br>_batch1
Q8N668	COMMD1	1	190	MAAGELEGGKPLSGLLNALAQDTFHGYPGITEELLRSQLYPEVPPEEFRPFLAKMRGILKSIASADMDFNQLEAFLTAQTKKQGGITSDQAAVISKFWKSHKTKIRESLMNQSRWNSGLRGLSWRVDGKSQSRHSAQIHTPVAIIELELGKYGQESEFLCLEFDEVKVNQILKTLSEVEESISTLISQPNLEHHHHHH	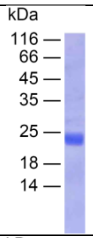	Group6<br>_batch1
P00167	CYB5A	1	108	MAEQSDEAVKYYTLEEIQKHNHSKSTWLILHHKVYDLTKFLEEHPGGEEVLREQAGGDATENFEDVGHSTDAREMSKTFIIGELHPDDRPKLNKPPETLITTIDSSSSLEHHHHHH	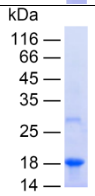	Group6<br>_batch1
Q12798	CETN1	1	172	MASGFKKPSAASTGQKRKVAPKPELTEDQKQEVREAFDLFDVDGSGTIDAKELKVAMRALGFEPRKEEMKKMISEVDREGTGKISFNDFLAVMTQKMSEKDTKEEILKAFRLFDDDETGKISFKNLKRVANELGENLTDEELQEMIDEADRDGDGEVNEEEFLRIMKKTSLYLEHHHHHH	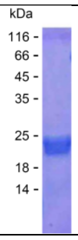	Group6<br>_batch1
P50053	KHK	1	298	MEEKQILCVGLVVLDVISLVDKYPKEDSEIRCLSQRWQRGGNASNSCTVLSLLGAPCAFMGSMAPGHVADFLVADFRRRGVDVSQVAWQSKGDTPSSCCIINNSNGNRTIVLHDTSLPDVSATDFEKVDLTQFKWIHIEGRNASEQVKMLQRIDAHNTRQPPEQKIRVSVEVEKPREELFQLFGYGDVVFVSKDVAKHLGFQSAEEALRGLYGRVRKGAVLVCAWAEEGADALGPDGKLLHSDAFPPPRVVDTLGAGDTFNASVIFSLSQGRSVQEALRFGCQVAGKKCGLQGFDGIVLEHHHHHH	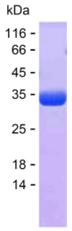	Group6<br>_batch1
Q16181	SEPTIN7	1	437	MSVSARSAAAAEERSVNSSTMVAQQKNLEGYVGFANLPNQVYRKSVKRGFEFTLMVVGESGLGKSTLINSLFLTDLYSPEYPGPSHRIKKTVQVEQSKVLIKEGGVQLLLTIVDTPGFGDAVDNSNCWQPVIDYIDSKFEDYLNAESRVNRRQMPDNRVQCCLYFIAPSGHGLKPLDIEFMKRLHEKVNIIPLIAKADTLTPEECQQFKKQIMKEIQEHKIKIYEFPETDDEEENKLVKKIKDRLPLAVVGSNTIIEVNGKRVRGRQYPWGVAEVENGEHCDFTILRNMLIRTHMQDLKDVTNNVHYENYRSRKLAAVTYNGVDNNKNKGQLTKSPLAQMEEERREHVAKMKKMEMEMEQVFEMKVKEKVQKLKDSEAELQRRHEQMKKNLE	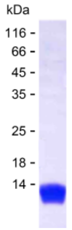<br>表达后降解，此带带降解带	Group7<br>_batch1

				AQHKELEEKRRQFEDEKANWEAQQRILEQQNSSRTLEK<br>NKKKGKIFLEHHHHHH		
P24844	MYL9	1	172	MSSKRAKAKTTKKRPQRATSNVFAMFDQSQIQEFKEAFN<br>MIDQNRDGFIDKEDLHDMLASLGKNPTDEYLEGMMSEA<br>PGPINFTMFLTMFGEKLNGTDPEDVIRNAFACFDEEASGF<br>IHEDHLRELLTTMGDRFTDEEVDEMYREAPIDKKGNFNY<br>VEFTRILKHGAKDKDDLEHHHHHH	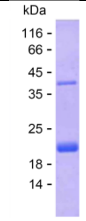	Group7<br>_batch1
Q9BV20	MRI1	1	369	MTLEAIRYSRGSLQILDQLLLPKQSRYEAVGSVHQAWEAI<br>RAMKVRGAPAIALVGCLSLAVELQAGAGGPGLAALVAFV<br>RDKLSFLVTARPTAVNMARAARDLADVAAREAEREGATE<br>EAVRERVICCTEDMLEKDLRDNRSIGDLGARHLLERVAPS<br>GGKVTVLTHCNTGALATAGYGTALGVIRSLHSLGRLEHAF<br>CTETRPYNQGARLTAFELVYEQIPATLITDSMVAAAMAH<br>RGVSAVVVGADRVVANGDTANKVGTYQLAIVAKHHGIP<br>FYVAAPSSSCDLRLETGKEIIIEERPGQELTDVNGVRIAAPG<br>IGVWNPAFDVTPHDLITGGIITELGVFAPEELRTALTTTISS<br>RDGTLDGPQMLEHHHHHH	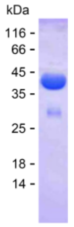	Group7<br>_batch1
P17302	GJA1	232	382	MFFKGVKDRVKGKSDPYHATSGALSPAKDCGSQKYAYF<br>NGCSSPTAPLSPMSPPGYKLVTGDRNNSSCRNYNKQAS<br>EQNWANYSAEQNRMGQAGSTISNSHAQPFDFPDDNQ<br>NSKKLAAGHELQPLAIVDQRPSSRASSRASSRPRPDDLEIL<br>EHHHHHH	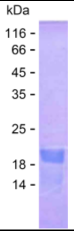	Group7<br>_batch1
Q8IV38	ANKMY2	1	441	MVHIKKGELTQEEKELLEVIGKGTVQEAGTLLSSKNVRVN<br>CLDENGMTPLMHAAYKGKLDMCKLLLRHGADVNCHQ<br>HEHGYTALMFAALSGNKDITWVMLEAGAETDVVNSVGR<br>TAAQMAAFVGQHDCVTIINNFFPRERLDYYTKPQGLDKE<br>PKLPPKLAGPLHKIITTTNLHPVKIVMLVNENPLLTEEAAL<br>NKCYRVMDLICEKCMKQRDMNEVLAMKMHYISCIFQKC<br>INFLKDGENKLDTLIKSLLKGRASDGFPVYQEKIIRESIRKFP<br>YCEATLLQQLVRSIAPVEIGSDPTAFSVLTQAITGQVGFVD<br>VEFCTTCGEKGASKRCSVCKMVIYCDQTCQKTHWFTHK<br>KICKNLKDIYEKQQLEAAKEKRQEENHGKLDVNSNCVNE<br>EQPEAEVGISQKDSNPEDSGEGKKESLESEAELEGLQDAP<br>AGPQVSEELEHHHHHH	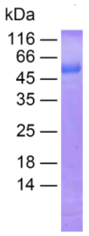	Group7<br>_batch1
Q03013	GSTM4	1	218	MSMTLGYWDIRGLAHAIRLLLEYTDSSYEEKKYTMGDAP<br>DYDRSQWLNEKFKLGLDFPNLPYLIDGAHKITQSNAILCYI<br>ARKHNLCGETEEEKIRVDILENQAMDVSNQLARVCYSPD<br>FEKLKPEYLEELPTMMQHFSQFLGKRPWFVGDKITFVDFL<br>AYDVLDLHRIFEPNCLDAFPNLKDFISRFEGLEKISAYMKS<br>SRFLPKPLYTRVAVWGNKLEHHHHHH	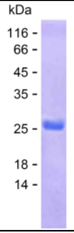	Group7<br>_batch1
Q96LJ8	UBXN10	1	280	MATEAPVNIAPPECSTVVSTAVDSLIWQPNSLNMHMIRP<br>KSAKGRTRPSLQKSQGVEVCAHHIPSPPPAIPYELPSSQK<br>PGACAPKSPNQGASDEIPELQQQVPTGASSSLNKYPVLP<br>SINRKNLEEEAVETVAKKASSLQLSSIRALYQDETGTMKTS<br>EEDSRARACAVERKFIVRTKKQGSSRAGNLEEPSDQEPRL<br>LLAVRSPTGQRFVRHFRPTDDLQTIVAVAEQKNKTSYRH<br>CSIETMEVPRRRFSDLTKSLQECRIPHKSVLGISLEDGEGW<br>PLEHHHHHH	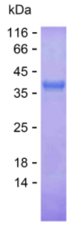	Group7<br>_batch1

Q86SG5	S100A7A	1	101	MSNTQAERSIIGMIDMFHKYTGRDGKIEKPSLLTMMKEN<br>FPNFLSACDKKGIHYLATVFEKKDKNEDKKIDFSEFLSLLG<br>DIAADYHKQSHGAAPCSGGSQLEHHHHHH	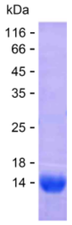	Group7<br>_batch1
P35557	GCK	1	465	MLDDRARMEAAKKEKVEQILAEFQLQEEDLKKVMRRMQ<br>KEMDRGLRLETHEEASVKMLPTYVRSTPEGSEVGDFLSLD<br>LGGTNFRVMLVKVGEGEEGQWSVKTKHQMYSIPEDAM<br>TGTAEMLFDYISECISDFLDKHQMKHKKLPLGFTFSFPVR<br>HEDIDKGILLNWTKGFKASGAEGNNVVGLLRDAIKRRGD<br>FEMDVVAMVNDTVATMISCYYEDHQCEVGMIVGTGCN<br>ACYMEEMQNVELVEGDEGRMCVNTEWGAFGDSGELD<br>EFLLEYDRLVDESSANPGQQLYEKLIGGKYMGELVRLVLL<br>RLVDENLLFHGEASEQLRTRGAFETRFVSQVESDTGDRK<br>QIYNILSTLGLRPSTTDCDIVRRACESVSTRAAHMCSAGL<br>AGVINRMRESRSEDVMRITVGVDGSVYKLHPSFKERFHA<br>SVRRLTPSCEITFIESEEGSGRGAALVSAVACKKACMLGQL<br>EHHHHHH	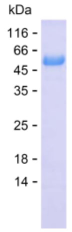	Group8<br>_batch1
Q5VW32	BROX	1	408	MTHWFHRNPLKATAPVSFNYYGVVTGPSASKICNDLRSS<br>RARLLELFTDLSCNPEMMKNAADSYFSLLQGFINSLDEST<br>QESKLRYIQNFKWTDTLQGQVPSAQQDAVFELISMGFN<br>VALWYTKYASRLAGKENITEDEAKEVHRSLKIAAGIFKHLK<br>ESHLPKLITPAEKGRDLESRLIEAYVIQCQAEAQEVTIARAI<br>ELKHAPGLIAALAYETANFYQKADHTLSSLEPAYSAKWRK<br>YLHLKMCFYTAYAYCYHGETLLASDKCGEAIRSLQEAEKL<br>YAKAEALCKEYGETKGPGPTVKPSGHLFFRKLGNLVKNTL<br>EKCQRENGFIYFQKIPTEAPQLELKANYGLVEPIPFEFPPTS<br>VQWTPETLAAFDLTKRPKDDSTKPKPEEEVKPVKEPDIKP<br>QKDTGCLEHHHHHH	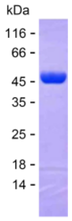	Group8<br>_batch1
Q9Y3B8	REXO2	26	237	MVREGGAAMAAGESMAQRMVWVDLEMTGLDIEKDQII<br>EMACLITDSDLNILAEGPNLIIKQPDELLDSMSDWCKEHH<br>GKSGLTKAVKESTITLQQAEYEFLSFVRQQTPPGLCPLAG<br>NSVHEDKKFLDKYMPQFMKHLHYRIIDVSTVKELCRRWY<br>PEEYEFAPKKAASHRALDDISESIKELQFYRNNIFKKKIDEK<br>KRKIIENGENEKTVSLEHHHHHH	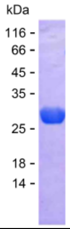	Group8<br>_batch1
P30405	PPIF	30	207	MCSKGSGDPSSSSSSGNPLVYLDVDANGKPLGRVVLELK<br>ADVVPKTAENFRALCTGEKGFGYKGSTFHRVIPSFMCQA<br>GDFTNHNGTGGKSIYGSRFPDENFTLKHVGPGVLSMAN<br>AGPNTNGSQFFICTIKTDWLDGKHVVFGHVKEGMDVVK<br>KIESFGSKSGRTSKKIVITDCGQLSLEHHHHHH	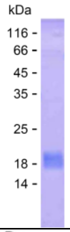	Group8<br>_batch1
Q96A00	PPP1R14A	1	147	MAAQRLGKRVLSKLQSPSRARGPGGSPGGLQKRHARVT<br>VKYDRRELQRRLDVEKWIDGRLEELYRGMEADMPDEINI<br>DELLELESEEERSRKIQGLLKSCGKPVEDFIQELLAKLQGLH<br>RQPGLRQPSPSHDGSLSPLQDRARTAHPLEHHHHHH	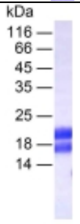	Group8<br>_batch1
A6NEY8	PRORSD1P	1	169	MAGAELGAALEQRLGALAIHTEVVEHPEVFTVEEMMPHI<br>QHLKGAHSKNLFLKDKKKKNYWLVTVLHDRQINLNELAK<br>QLGVGSGNLRFADETAMLEKLKVGQGCATPLALFCDGG<br>DVKFVLDSAFLEGGHEKVYFHPMTNAATMGLSPEDFLTF<br>VKMTGHDPIILNFDLEHHHHHH	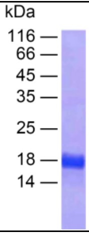	Group8<br>_batch1

P34896	SHMT1	1	483	MTMPVNGAHKDADLWSSHDKMLAQPLKDSDVEVYNIIKKESNRQRVGLELIASENFASRAVLEALGSCLNNKYSEGYPGQRYYGGTEFIDELETLCQKRALQAYKLDPQCWGVNVQPYSGSPANFAVYTALVEPHGRIMGLDLPDGGHLTHGFMTDKKKISATSIFFESMPYKVNPDTGYINYDQLEENARLFHPKLIIAGTSCYSRNLEYARLRKIADENGAYLMADMAHISGLVAAGVVPSPFEHCHVVTTTTHKTLRGCRAGMIFYRKGVKSVDPKTGKEILYNLESLINSAVFPGLQGGPHNHAIAGVAVALKQAMTLEFKVYQHQVVANCRALSEALTELGYKIVTGGSDNHLILVDLRSKGTDGGRAEKVLEACSIACNKNTCPGDRSALRPSGLRLGTPALTSRGLLEKDFQKVAHFIHRGIELTLQIQSDTGVRATLKEFKERLAGDKYQAAVQALREEVESFASLFPLPGLPDFLEHHHHHH	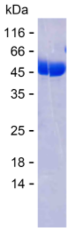	Group8 _batch1
O75293	GADD45B	1	160	MTLEELVACDNAAQKMQTVTAAVEELLVAAQRQDRLTVGVYESAKLMNVDPDSVVLCLLAIDEEEEDDIALQIHFTLIQSFCCDNDINIVRVSGMQRLAQLLGEPAETQGTTEARDLHCLLVTNPHTDAWKSHGLVEVASYCEESRGNNQWVPYISLQERLEHHHHHH	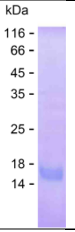	Group8 _batch1
Q12974	PTP4A2	1	164	MNRPAPVEISYENMRFLITHNPTNATLNKFTEELKKYGVTTLVRVCDATYDKAPVEKEGIHVLDWPFDDGAPPPNQIVDDWLNLLKTKFREEPGCCVAVHCVAGLGRAPVLVALALIECGMKYEDAVQFIRQKRRGAFNSKQLLYLEKYRPKMRLRFRDTNGHCLEHHHHHH	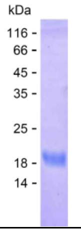	Group1 _batch3
P54284	CACNB3	1	484	MYDDSYVPGFEDSEAGSADSYTSRPSLDSDVSLEEDRESARREVESQAQQQLERAKHKPVAFAVRTNVSYCGVLDEECPVQGSGVNFEAKDFLHIKEKYSNDWWIGRLVKEGGDIAFIPSPQRLESIRLKQEQKARRSGNPSSLSDIGNRRSPPPSLAKQKQKQAEHVPPYDVVPSMRPVVLVGPSLKGYEVTDMMQKALFDFLKHRFDGRISITRVTADLSLAKRSVLNNPGKRTIIERSSARSSIAEVQSEIERIFELAKSLQLVVLDADTINHPAQLAKTSLAPIIVFVKVSSPKVLQRLIRSRGKSQMKHLTVQMMAYDKLVQCPPESFDVILDENQLEDACEHLAEYLEVYWRATHHPAPGPGLLGPPSAIPGLQNQQLLGERGEEHSPLERDSLMPSDEASESSRQAWTGSSQRSSRHLEEDYADAYQDLYQPHRQHTSGLPSANGHDPQDRLLAQDSEHNHSDRNWQRNRPWPKDSYLEHHHHHH	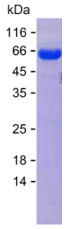	Group1 _batch3
P63000	RAC1	1	189	MQAIKCVVVGDGAVGKTCLLISYTTNAFPGEYIPTVFDNYSANVMVDGKPVNLGLWDTAGQEDYDRLRPLSYPQTDVFLICFSLVSPASFENVRAKWYPEVRHHCPNTPIILVGTKLDLRDDKDTIEKLKEKKLTPITYPQGLAMAKEIGAVKYLECSALTQRGLKTVFDEAIRAVLCPPPVKKRKRKCLEHHHHHH	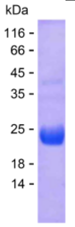	Group1 _batch3
O43768	ENSA	1	121	MSQKQEEENPAEETGEEKQDTQEKEGILPERAEEAKLKAKYPSLGQKPGGSDFLMKRLQKGQKYFDSGDYNMAKAKMKNKQLPSAGPDKNLVTGDHIPTPQDLPQRKSSLVTSKLAGGQVELEHHHHHH	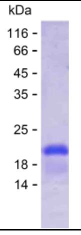	Group1 _batch3

O95881	TXNDC12	27	172	MHNGLGKGFGDHIHWRTLEDGKKEAAASGLPLMVIIHKSWCGACKALKPKFAESTEISELSHNFVMVNLEDEEEPKDEDFSPDGGYIPRILFLDPSGKVHPEIINENGNPSYKYFYVSAEQVVQGMKEAQERLTGDAFRKKHLEDELEHHHHHH	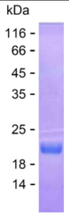	Group1 _batch3
Q9NR45	NANS	1	359	MPLELELCPGRWVGGQHPCFIIAEIGQNHQGDLDVAKRMIRMAKECGADCAKFQKSELEFKFNRKALERPYTSKHSWGKTYGEHKRHLEFSHDQYRELQRYAEEVGIFFTASGMDEMAVEFLHELNVPFFKVGSGDTNNFPYLEKTAKKGRPMVISSGMQSMDTMKQVYQIVKPLNPNFCFLQCTSAYPLQPEDVNLRVISEYQKLFPDIPIGYSGHETGIAISVAAVALGAKVLERHITLDKTWKGSDHSASLEPGELAELVRSVRLVERALGSPTKQLLPCEMACNEKLGKSVVAKVKIPEGTILTMDMLTVKVGEPKGYPPEDIFNLVGKKVLVTVEEDDTIMEELVDNHGKKIKSLEHHHHHH	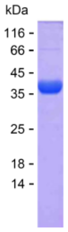	Group1 _batch3
Q9UK22	FBXO2	1	296	MDGDGDPESVGQPEEASPEEQPEEASAEEERPEDQQEEEAAAAAAAYLDELPEPLLLRVLAALPAAELVQACRLVCLRWKELVDGAPLWLLKCQQEGLVPEGGVEEERDHWQQFYFLSKRRRNLLRNPCGEEDLEGWCDVEHGGDGWRVEELPGDSGVEFTHDESVKKYFASSFEWCRKAQVIDLQAEGYWEELLDTTQPAIVVKDWYSGRSDAGCLYELTVKLLSEHENVLAEFSSGQVAVPQDSDGGGWMEISHTFTDYGPGVRFVRFEHGGQDSVYWKGWFGARVTNSSVWVEPLEHHHHHH	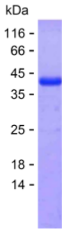	Group1 _batch3
B2RPK0	HMGB1P1	1	211	MGKGDPKKPRGKMSSYAFFVQTCREEHKKKHSDASVNFSEFSNKCSERWKTMSAKEKGKFEDMAKADKTHYERQMKTYIPPKGETKKKFKDPNAPKRPPSAFFLFCSEYHPKIKGEHPGLSIGDVAKKLGEMWNNTAADDKQPGEKKAAKLKEKYEKDIAAYQAKGKPEAAKKGVVKAEKSKKKKEEEEDEEDEDEEEEDEEDEEDDDDELEHHHHHH	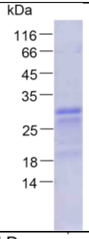	Group2 _batch3
O95777	LSM8	1	96	MTSALENYINRTVAVITSDGRMIVGTLKGFDQTINLILDESHERVFSSSQGVEQVVLGLYIVRGDNVAVIGEIDEETDSALDLGNIRAEPLNSVAHLEHHHHHH	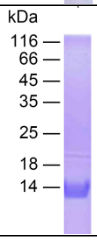	Group2 _batch3
P04424	ASL	1	464	MASESGKLWGGRFVGAVDPIMEKFNASIAYDRHLWEVDVQGSKAYSRGLEKAGLLTKAEMDQILHGLDKVAEEWAQGTFKLNSNDEDIHTANERRLKELIGATAGKLHTGRSRNDQVVTDLRLWMRQTCSTLSGLLWELIRTMVDRAEAERDVLFPGYTHLQRAQPIRWSHWILSHAVALTRDSERLLEVRKRINVLPLGSGAIAGNPLGVDRELLRAELNFGAITLNSMDATSERDFVAEFLFWASLCMTHLSRMAEDLILYCTKEFSFVQLSDAYSTGSSLMPQKKNPDSLELIRSKAGRVFGRCAGLLMTLKGLPSTYNKDLQEDKEAVFEVSDTMSAVLQVATGVISTLQIHQENMGQALSPDMLATDLAYYLVRKGMPFRQAHEASGKAVFMAETKGVALNQLSLQELQTISPLFSGDVICVWDYGHSVEQYGALGGTARSSVDWQIRQVRALLQAQQALEHHHHHH	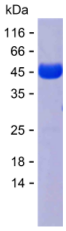	Group2 _batch3
Q13325	IFIT5	1	482	MSEIRKDTLKAILLELECHFTWNLLKEDIDLFEVEDTIGQQLEFLTTKSRLALYNLLAYVKHLKGQNKDALECLEQAEEIIQQEHSDKEEVRSLVTWGNYAWVYYHMDQLEEAQKYTGKIGNVCKKLSSPSNYKLECPETDCEKGWALLKFGGKYYQKAKAAFEKALEVEPDNPEFNIGYAITVYRLDDSDREGSVKSFSLGPLRKAVTLNPDNSYIKVFLALKLQDVHAEAEGEKYIEEILDQISSQPYVLRYAAKFYRRKNSWNKALELLKKALEVTPTS	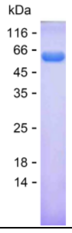	Group2 _batch3

				SFLHHQMGLCYRAQMIQIKKATHNRPKGKDKLKVDELIS<br>SAIFHFKAAMERDSMFAFAYTDLANMYAEGGQYSNAED<br>IFRKALRLENITDDHKHQIHYHYGRFQEFHRKSENTAIHH<br>YLEALKVKDRSPLRTKLTSALKKLSTKRLCHNALDVQSLSA<br>LGFVYKLEGEKRQAAEYYEKAQKIDPENAEFLTALCELRLSI<br>LEHHHHHH		
Q15365	PCBP1	1	356	MDAGVTESGLNVTLTIRLLMHGKEVGSIIGKKGESVKRIRE<br>ESGARINISEGNCPERIITLTGPTNAIFKAFAMIIDKLEEDIN<br>SSMTNSTAASRPPVTLRLVVPATQCGSLIGKGGCKIKEIRE<br>STGAQVQVAGDMLPNSTERAITIAGVPQSVTECVKQICL<br>VMLETLSQSPQGRVMTIPYQPMPASSPVICAGGQDRCS<br>DAAGYPHATHDLEGPPLDAYSIQGQHTISPLDLAKLNQV<br>ARQQSHFAMMHGGTGFAGIDSSSPEVKGYWASLDAST<br>QTTHELTIPNNLIGCIIGRQGANINEIRQMSGAQIKIANPV<br>EGSSGRQVTITGSAASISLAQYLINARLSSEKGMGCSLEH<br>HHHHH	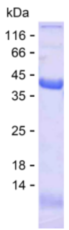	Group2<br>_batch3
Q96NU7	AMDHD1	1	426	MASGHSLLLENAQQVVLVCARGERFLARDALRSLAVLEG<br>ASLVVGKDGFIKAIGPADVIQRQFSGETFEEIIDCSGKCILP<br>GLVDAHTHPVWAGERVHEFAMKLAGATYMEIHQAGGG<br>IHFTVERTRQATEEELFRSLQQRLQCMMRAGTTLVECKS<br>GYGLDLETELKMLRVIERARRELDIGISATYCGAHSVPKGK<br>TATEAADDIINNHLPKLKELGRNGEIHVDNIDVFCEKGVF<br>DLDSTRRILQRGKDIGLQINFHGDELHPMKAAELGAELG<br>AQAISHLEEVSDEGIVAMATARCSAILLPTTAYMLRLKQP<br>RARKMLDEGVIVALGSDFNPNAYCFSMPMVMHLACVN<br>MRMSMPEALAAATINAAYALGKSHTHGSLEVGKQGDLII<br>INSSRWEHLIYQFGGHHELIEYVIAKGKLIYKTLEHHHHHH	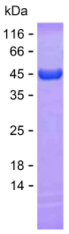	Group2<br>_batch3
Q96PI1	SPRR4	1	79	MSSQQQQRQQQQCPPQRAQQQQVKQPCQPPPVKC<br>QETCAPKTKDPCAPQVKKQCPPKGTIIPAQQKCPSAQQ<br>ASKSKQKLEHHHHHH	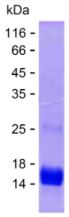	Group2<br>_batch3
Q6PF06	TRMT10B	1	316	MDWKLEGSTQKVESPVLQGQEGILEETGEDGLPEGFQLL<br>QIDAEGECQEGEILATGSTAWCSKNVQRKQRHWEKIVA<br>AKKSKRKQEKERRKANRAENPGICPQHSKRFLRALTKDKL<br>LEAKHSGPRLCIDLSMTHYMSKKELSRLAGQIRRLYGSNK<br>KADRPFWICLTGFTTDSPLYEECVRMNDGFSSYLLDITEE<br>DCFSLFPLETLVYLTPDSEHALEDVDLNKVYILGGLVDESI<br>QKKVTFQKAREYSVKTARLPIQEYMVRNQNGKNYHSEIL<br>AINQVFDILSTYLETHNWPEALKKGVSSGKGYILRNSVELE<br>HHHHHH	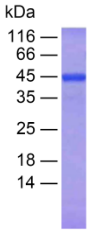	Group2<br>_batch3
O00757	FBP2	1	339	MTDRSPFETDMLTLTRYVMEKGRQAKGTGELTQLLNSM<br>LTAIKAISSAVRKAGLAHLYGIAGSVNVTGDEVKKLDVLS<br>NSLVINMVQSSYSTCVLVSEENKDAIITAKEKRGKYVVCF<br>DPLDGSSNIDCLASIGTIFAIYRKTSEDEPSEKDALQCGRNI<br>VAAGYALYGSATLVALSTGQGVDLFMLDPALGEFVLVEK<br>DVKIKKKGKIYSLNEGYAKYFDAATTEYVQKKKFPEDGSA<br>PYGARYVGSMVADVHRTLVYGGIFLYPANQKSPKGKLRL<br>LYECNPVAYIIEQAGGLATTGTQPVLDVKPEAIHQRVPLIL<br>GSPEDVQEYLTCVQKNQAGSLEHHHHHH	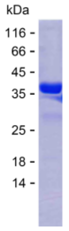	Group3<br>_batch3
P60520	GABARAPL2	1	116	MKWMFKEDHSLEHRCVESAKIRAKYPDRVPVIVEKVSGS<br>QIVDIDKRKYLVPSDITVAQFMWIIRKRIQLPSEKAIFLFVD<br>KTVPQSSLTMGQLYEKEKDEDGFLYVAYSGENTFGLEHH<br>HHHH	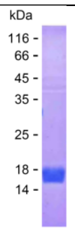	Group3<br>_batch3

P62253	UBE2G1	1	170	MTELQSALLLRRQLAELNKNPVEGFSAGLIDDNDLYRWEVLIIGPPDTLYEGGVFKAHLTFPKDYPLRPPKMKFITEIWHPNVDKNGDVCISILHEPGEDKYGYEKPEERWLPIHTVETIMISVISMLADPNGDSPANVDAAKEWREDRNGEFKRKVARCVRKSQETAFELEHHHHHH	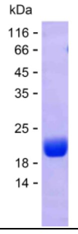	Group3 _batch3
Q6IBS0	TWF2	1	349	MAHQTGIHATEELKEFFAKARAGSVRLIKVVIEDEQLVLGASQEPVGRWDQDYDRAVLPLLDAQQPCYLLYRLDSQNAQGFEWLFLAWSPDNSPVRLKMLYAATRATVKKEFGGGHIKDELFGTVKDDLSFAGYQKHLSSCAAPAPLTSAERELQQIRINEVKTEISVESKHQTLQGLAFPLQPEAQRALQQLKQKMVNYIQMKLDLERETIELVHTEPTDVAQLPSRVPRDAARYHFFLYKHTHEGDPLESVVFIYSMPGYKCSIKERMLYSSCKSRLLDSVEQDFHLEIAKKIEIGDGAELTAEFLYDEVHPKQHAFKQAFAKPKGPGGKRGHKRLIRGPGENGDDSLEHHHHHH	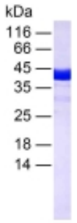	Group3 _batch3
Q9NQP4	PFDN4	1	134	MAATMKKAAAEDVNVTFEDQQKINKFARNTSRITELKEEIEVKKKQLQNLEDACDDIMLADDDCLMIPYQIGDVFISHSQEETQEMLEEAKKNLQEEIDALESRVESIQRVLADLKVQLYAKFGSNINLEADESLEHHHHHH	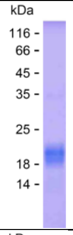	Group3 _batch3
Q15004	PCLAF	1	111	MVRTKADSVPGTYRKVVAARAPRKVLGSSTSATNSTSVSSRKAENKYAGGNPVCVRPTPKWQKGIGEFFRLSPKDSEKENQIPEEAGSSGLGKAKRKACPLQPDHTNDEKELEHHHHHH	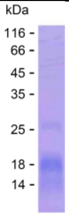	Group3 _batch3
Q9NP86	CABP5	1	173	MQFPMGPACIFLRKGIAEKQRERPLGQDEIEELREAFLEFDKDRDGFISCKDLGNLMRTMGYMPTEMELIELGQQIRMNLGGRVDFDDFVELMTPKLLAETAGMIGVQEMRDAFKEFDTNGDGEITLVELQQAMQRLLGERLTPREISEVVREADVNGDGTVDFEEFVKMMSRLEHHHHHH	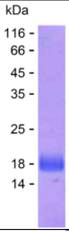	Group3 _batch3
Q5VXD3	SAMD13	1	122	MANSLLEGVFAEVKEPCSLPMLSVDMENKENGSVGVKNSMENGRPPDPADWAVMDVVNYFRTVGFEEQASAFQEQEIDGKSLLLMTRNDVLTGLQLKLGPALKIYEYHVKPLQTKHLKNNSSLEHHHHHH	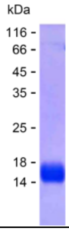	Group3 _batch3
Q9P2T1	GMPR2	1	348	MPHIDNDVKLDFKDVLLRPKRSTLKSRSEVDLTRSFSFRNSKQTYSGVPIIAANMDTVGTFEMAKVLCKFSLFTAVHKHYSLVQWQEFAGQNPDCLEHLAASSGTGSSDFEQLEQILEAIPQVKYICLDVANGYSEHFVEFVKDVRKRFPQHTIMAGNVVTGEMVEELILSGADIIKVGIGPGSVCTTRKKTGVGYPQLSAVMECADAAHGLKGHIISDGGCSCPGDVAKAFGAGADFVMLGGMLAGHSESGGELIERDGKKYKLFYGMSSEMAMKKYAGGVAEYRASEGKTVEVPFKGDVEHTIRDILGGIRSTCTYVGAAKLKELSRRTTFIRVTQQVNPIFSEACLEHHHHHH	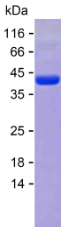	Group4 _batch3

P35236	PTPN7	1	360	MVQAHGGRSRAQPLTLSLGAAMTQPPPEKTPAKKHVRL<br>QERRGSNVALMLDVRSLGAVEPICSVNTPREVTLHFLRTA<br>GHPLTRWALQRQPPSPKQLEEEFLKIPSNFVSPEDLDIPG<br>HASKDRYKTILPNPQSRVCLGRAQSQEDGDYINANYIRG<br>YDGKEKVYIATQGPMPNTVSDFWEMVWQEEVSLIVMLT<br>QLREGKEKCVHYWPTEEETYGPFQIRIQDMKECPEYTVR<br>QLTIQYQEERRSVKHILFSAWPDHQTPESAGPLLRLVAEV<br>EESPETAAHPGPIVVHCSAGIGRTGCFIATRIGCQQLKAR<br>GEVDILGIVCQLRLDRGGMIQTAEQYQFLHHTLALYAGQ<br>LPEEPSPLEHHHHHH	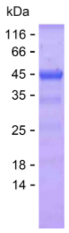	Group4<br>_batch3
Q06136	KDSR	26	270	MKPLALPGAHVVVTGGSSGIGKCIAIECYKQGAFITLVAR<br>NEDKLLQAKKEIEMHSINDKQVVLCISVDVSQDYNQVEN<br>VIKQAQEKLGPVDMLVNCAGMAVSGKFEDLEVSTFERL<br>MSINYLGSVYPSRAVITTMKERRVGRIVFVSSQAGQLGLF<br>GFTAYSASKFAIRGLAEALQMEVKPYNVYITVAYPPDTDT<br>PGFAEENRTKPLETRLISETTSVCKPEQVAKQIVKDAIQGN<br>FNSSLGSDLEHHHHHH	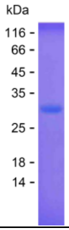	Group4<br>_batch3
Q9BUH6	PAXX	1	204	MDPLSPPLCTLPPGPEPPRFVCYCEGEESGEGDRGGFNLY<br>VTDAAELWSTCFTPDSLAALKARFGLSAAEDITPRFRAAC<br>EQQAVALTLQEDRASLTLSGGPSALAFDLSKVPGPEAAP<br>RLRALTLGLAKRVWSLERRLAAAEETAVSPRKSPRPAGPQ<br>LFLPDPDPQRGGPGPGVRRRCPGESLINPGFKSKKPAGG<br>VDFDETLEHHHHHH	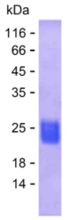	Group4<br>_batch3
Q9NV35	NUDT15	1	164	MTASAQPRGRRPGVGVGVVVTSCKHPRCVLLGKRKGSV<br>GAGSFQLPGGHLEFGETWEECAQRETWEEAALHLKNVH<br>FASVVNSFIEKENYHYVTILMKGEVDVTHDSEPKNVEPEK<br>NESWEWVPWEELPPLDQLFWGLRCLKEQGYDPFKEDLN<br>HLVGYKGNHLEHHHHHH	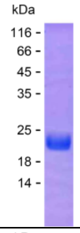	Group4<br>_batch3
Q5T215	TRAPPC3L	1	181	MSRPAHRRPEYHKINKDLFVLTYGALVAQLCKDYEKDED<br>VNQYLDKMGYGIGTRLVEDFLARSCVGRCHSYSEIIDIIAQ<br>VAFKMYLGITPSVTCNNSSKNEFSLILEKNPLVEFVEELPA<br>GRSSLCYCNLLCGIIRGALEMVHLAADVTFLQDRLKGDSV<br>TEIGITFLKKRDEKKYRGKKLEHHHHHH	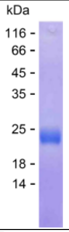	Group4<br>_batch3
Q7RTT5	SSX7	1	188	MNGDDAFARRPRAGAQIPEKIQKSFDDIAKYFSKKEWEK<br>MKSLEKISYVYMKRKYEAMTKLGFKATLPPFMHNTGATD<br>LQGNDFDNDRNQGNQVERPQMTFCRLQRIFPKIMPKK<br>PAEEGNDSKGVPEASGSQNDGKHLCPPGKPSTSEKINKT<br>SGPKRGKHAWTHRLRERKQLVIYEEISDPEEDDELEHHHH<br>HH	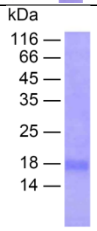	Group4<br>_batch3
A6NMZ2	SNTN	1	147	MGGCMHSTQDKSLHLEGDPNPSAAPTSTCAPRKMPKRI<br>SISKQLASVKALRKCSDLEKAIATTALIFRNSSDSDGKLEKA<br>IAKDLLQTQFRNFAEGQETKPKYREILSELDEHTENKLDFE<br>DFMILLLSITVMSDLLQNIRNVKIMKLEHHHHHH	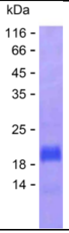	Group4<br>_batch3
P13866	SLC5A1	564	643	MRNSKEERIDLDAEEENIQEGPKETIEIETQVPEKKKGIFRR<br>AYDLFCGLEQHGAPKMTEEEEKAMKMKMTDTSEKPLWR<br>TLEHHHHHH	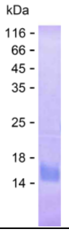	Group5<br>_batch3

Q96DE0	NUDT16	1	195	MAGARRLELGEALALGSGWRHACHALLYAPDPGMLFGR IPLRYAILMQMRFDGRLGFPGGFVDTQDRSLEDGLNREL REELGEAAAAFRVERTDYRSSHVGSGPRVVAHFYAKRLTL EELLAVEAGATRAKDHGLEVLGLVRVPLYTLRDGVGGLPT FLENSFIGSAREQLLEALQDLGLLQSGSISGLKIPAHHLEH HHHHH	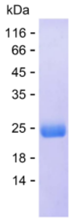	Group5 _batch3
P55809	OXCT1	40	520	MTKFYTDPVEAVKDIPDGATVLVGGFGLCGIPENLIDALL KTGVKGLTAVSNNAGVDNFGLGLLLRSKQIKRMVSSYVG ENAEFERQYLSGELEVELTPQGTLAERIRAGGAGVPAFYT PTGYGTLVQEGGSPIKYNKDGSVAIASKPREVREFNGQHF ILEEAITGDFALVKAWKADRAGNVIFRKSARNFNLPMCKA AETTVVEVEEIVDIGAFAPEDIHIPQIYVHRLIKGEKYEKRIE RLSIRKEGDGEAKSAKPGDDVRERIIKRAALEFEDGMYAN LGIGIPLLASNFISPNITVHLQSENGVLGLGPYPRQHEADA DLINAGKETVTILPGASFFSSDESFAMIRGGHVDLTMLGA MQVSKYGDLANWMIPGKMVKGMGGAMDLVSSAKTKV VVTMEHSAKGNAHKIMEKCTLPLTGKQCVNRIITEKAVF DVDKKKGLTLIELWEGLTVDDVQKSTGCDFAVSPKLMPM QQIANLEHHHHHH	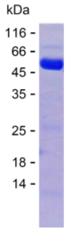	Group5 _batch3
P61204	ARF3	1	181	MGNIFGNLLKSLIGKKEMRILMVGLDAAGKTTILYKLKLGE IVTTIPTIGFNVETVEYKNISFTVWDVGGQDKIRPLWRHYF QNTQGLIFVVDSNDRERVNEAREELMRMLAEDELRDAV LLVFANKQDLPNAMNAAEITDKLGLHSLRHRNWYIQAT CATSGDGLYEGLDWLANQLKNKKLEHHHHHH	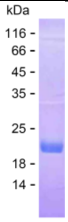	Group5 _batch3
A6NMN3	FAM170B	1	283	MKCYFTDHRGEQSPTDGTTLSLTSPESTEESVEVFWPGTI QREGSSPRPGPAIPREEGLYFAARDRGMRDWSSSPSSES SEYQSYSQYQSCCSCMCDEDNAAPQSVCAFYTHVQTV RGVAVAWETEAGFEPVTRKPRIHEAQFIKRQRWNGSSFE MASNTDMRWDLEACKSNCSPEPEDIDLLECCLQELREPP DWLVTTNYGVRCVACCRVLPSLDALLEHAQHGIREGFSC QIFFEEMLERRRAQGQAHDQQLEEEQSPSDNSECSRPQ GEVLSAQQQEKQLEHHHHHH	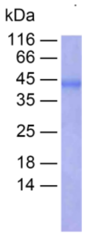	Group5 _batch3
Q9NRP4	SDHAF3	31	125	MGDQYVKDEFRRHKTVGSDEAQRFLQEWEVYATALLQ QANENRQNSTGKACFGTFLPEEKLNDFRDEQIGQLQEL MQEATKPNRQFSISESMKPKFLEHHHHHH	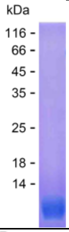	Group5 _batch3
A8MWE9	EFCAB8	1	144	MSSEDLAEIPQLQKLSIPHGFQNKEAASSPTPSITLSQVPD LQPGSQLFTEIHLAKIEKMFEEDINSTGALGMDAFIKAMK KVLSSVSDEMLKELFLKVDSDCEGFVTWQKYVDYMMRE FQGKEDMRKSQYRLHFYLPMTVVPLEHHHHHH	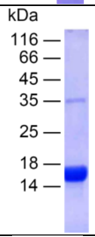	Group5 _batch3
Q5I0X7	TTC32	1	151	MEGQRQESHATLTLAQAHFNNGEYAEAEALYSAYIRRC ACAASSDESPGSKCSPEDLATAYNNRGQIKYFRVDFYEA MDDYTSAIEVQPNFEVPYYNRGLILYRLGYFDDALEDFKK VLDLNPGFQDATLSLKQTILDKEEKQRRNVAKNYLEHHH HHH	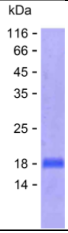	Group5 _batch3

P60900	PSMA6	1	246	MSRGSSAGFDRHITIFSPEGRLYQVEYAFKAINQGGLTSVAVRGKDCAVIVTQKKVPDKLLDSSTVTHLFKITENIGCVMTGMTADSRSQVQRARYEAANWKYKYGYEIPVDMLCKRIADISQVYTQNAEMRPLGCCMILIGIDEEQGPQVYKCDPAGYYCGFKATAAGVKQTESTSFLEKKVKKKFDWTFEQTVETAITCLSTVLSIDFKPSEIEVGVVTVENPKFRILTEAEIDAHLVALAERDLEHHHHHH	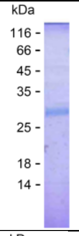	Group6 _batch3
P18825	ADRA2C	232	379	MRIYRVAKLRTRTLSEKRAPVGPDGASPTTENGLGAAAGAGENGHCAPPPADVEPDESSAAAERRRRRGALRRGGRRRAGAEGGAGGADGQGAGPGAAESGALTASRSPGPGGRLSRASSRSVEFFLSRRRRARSSVCRRKVAQAREKRLEHHHHHH	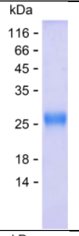	Group6 _batch3
Q96LZ3	PPP3R2	1	170	MGNEASYPAEMCSHFDNDEIKRLGRRFKKLDLDKSGSLSVEEFMSLPELRHNPLVRRVIDVFDTDGDGEVDFKEFILGTSQFSVKGDEEQKLRFAFSIYDMDKDGYISNGELFQVLKMMVGNNLTDWQLQQLVDKTIIILDKDGDGKISFEEFSAVVRDLEIHKKLVLIVLEHHHHHH	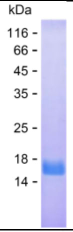	Group6 _batch3
P24534	EEF1B2	1	225	MGFGDLKSPAGLQVLNDYLADKSYIEGYVPSQADVAVFEAVSSPPPADLCHALRWYNHIKSYEKEKASLPGVKKALGKYGPADVEDTTGSGATDSKDDDDIDLFGSDDEEESEEAKRLREERLAQYESKKAKKPALVAKSSILLDVKPWDDETDMAKLEECVRSIQADGLVWGSSKLVPVGYGIKKLQIQCVVEDDKVGTDMLEEQITAFEDYVQSMDVAAFNKILEHHHHHH	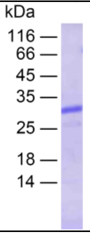	Group6 _batch3
P51608	MECP2	1	486	MVAGMLGLREEKSEDQDLQGLKDKPLKFKKVKKDKKEEKEGKHEPVQPSAHHSAEPAEAGKAETSEGSGSAPAVPEASASPKQRRSIIRDRGPMYDDPTLPEGWTRKLKQRKSGRSAGKYDVYLINPQGKAFRSKVELIAYFEKVGDTSLDPNDFDFTVTGRGSPSRREQKPPKKPKSPKAPGTGRGRGRPKGSGTTRPKAATSEGVQVKRVLEKSPGKLLVKMPFQTSPGGKAEGGGATTSTQVMVIKRPGRKRKAEADPQAIPKKRGRKPGSVVAAAAAEAKKKAVKESSIRSVQETVLPIKKRKTRETVSIEVKEVVKPLLVSTLGEKSGKGLKTCKSPGRKSKESSPKGRSSSASSPPKKEHHHHHHHSESPKAPVPLLPPLPPPPPEPESSEDPTSPPEPQDLSSSVCKEEKMPRGGSLESDGCPKEPAKTQPAVATAATAAEKYKHRGEGERKDIVSSSMPRPNREEPVDSRTPVTERVSLEHHHHHH	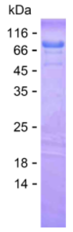	Group6 _batch3
Q16836	HADH	13	314	MSSSSTASASAKKIIVKHVTVIGGGLMGAGIAQVAAATGHTVVLVDQTEDILAKSKKGIEESLRKVAKKKFAENLKAGDEFVEKTLSTIATSTDAASVVHSTDLVVEAIVENLKVKNELFKRLDKFAAEHTIFASNTSSLQITSIANATTRQDRFAGLHFFNPVPVMKLVEVIKTPMTSQKTFESLVDFSKALGKHPVSCKDTPGFIVNRLLVPYLMEAIRLYERGDASKEDIDTAMKLGAGYPMGPFELLDYVGLDTTKFIVDGWHEMDAENPLHQPSPSLNKLVAENKFGKKTGEGFYKYKLEHHHHHH	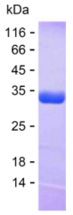	Group6 _batch3
Q9UNT1	RABL2B	1	228	MAEDKTKPSELDQGKYDADDNVKIICLGDSAVGKSKLMERFLMDGFQPQQLSTYALTLYKHTATVDGRTILVDFWDTAGQERFQSMHASYYHKAHACIMVFDVQRKVTYRNLSTWYTELREFRPEIPCIVVANKIDDINVTQKSFNFAKKFSLPLYFVSAADGTNVVKLFNDAIRLAVSYKQNSQDFMDEIFQELENFSLEQEEEDVPDQEQSSSIETPSEEAASPHSLEHHHHHH	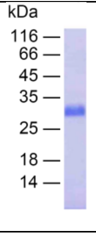	Group6 _batch3

Q7L8J4	SH3BP5L	1	393	MAELRQVPGGRETPQGELRPEVVEDEVPRSPVAEEPGGG<br>GSSSSEAKLSPREEEELDPRIQEELEHLNQASEEINQVELQ<br>LDEARTTYRRILQESARKLNTQGSHLGSCIEKARPYYEARR<br>LAKEAQQETQKAALRYERAVSMHNAAREMVFVAEQGV<br>MADKNRLDPTWQEMLNHATCKVNEAEEERLRGEREHQ<br>RVTRLCQQAEARVQALQKTLRRAIGKSRPYFELKAQFSQI<br>LEEHKAKVTELEQQVAQAKTRYSVALRNLEQISEQIHARR<br>RGGLPPHPLGPRRSSPVGAEAGPEDMEDGDSGIEGAEG<br>AGLEEGSSLGPGPAPDTDTLSLLSLRTVASDLQKCDSVEH<br>LRGLSDHVSLDGQELGTRSGGRRGSDGGARGGHQRSV<br>SLEHHHHHH	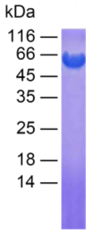	Group6<br>_batch3
P11310	ACADM	26	421	MKANRQREPGLGFSFEFTEQQKEFQATARKFAREEIIPVA<br>AEYDKTGEYPVPLIRRAWELGLMNTHIPENCGGLGLGTF<br>DACLISEELAYGCTGVQTAIEGNSLGQMPIIIAGNDQQKK<br>KYLGRMTEEPLMCAYCVTEPGAGSDVAGIKTKAEKKGDE<br>YIINGQKMWITNGGKANWYFLLARSDPDPKAPANKAFT<br>GFIVEADTPGIQIGRKELNMGQRCSDTRGIVFEDVKVPKE<br>NVLIGDGAGFKVAMGAFDKTRPVVAAGAVGLAQRALDE<br>ATKYALERKTFGKLLVEHQAISFMLAEMAMKVELARMSY<br>QRAAWEVDSGRRNTYYASIAKAFAGDIANQLATDAVQI<br>LGGNGFNTEYPVEKLMRDAKIYQIYEGTSQIQRLIVAREHI<br>DKYKNLEHHHHHH	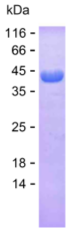	Group7<br>_batch3
Q9NPI5	NMRK2	1	230	MKLIVGIGGMTNGGKTTLTNSLLRALPNCCVIHQDDFFK<br>PQDQIAVGEDGFKQWDVLESLDMEAMLDTVQAWLSSP<br>QKFARAHGVSVQPEASDTHILLLEGFLLYSYKPLVDLYSRR<br>YFLTVPYEECKWRRSTRNYTVPDPPGLFDGHVWPMYQK<br>YRQEMEANGVEVVYLDGMKSREELFREVLEDIQNSLLNR<br>SQESAPSPARPARTQGPGRGCGHRTARPAASQQDSML<br>EHHHHHH	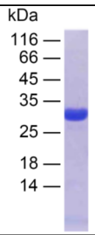	Group7<br>_batch3
P49189	ALDH9A1	1	494	MSTGTFVVSQPLNYRGGARVEPADASGTEKAFEPATGRV<br>IATFTCSGEKEVNLAVQNAKAAFKIWSQKSGMERCRILLE<br>AARIIREREDEIATMECINNGKSIFEARLDIDISWQCLEYYA<br>GLAASMAGEHIQLPGGSFGYTRREPLGVCVGIGAWNYPF<br>QIASWKSAPALACGNAMVFKPSPFTPVSALLLAEIYSEAG<br>VPPGLFNVVQGGAATGQFLCQHPDVAKVSFTGSVPTG<br>MKIMEMSAKGIKPVTLELGGKSPLIIFSDCDMNNAVKGAL<br>MANFLTQGQVCCNGTRVFVQKEILDKFTEEVVKQTQRIK<br>IGDPLLEDTRMGPLINRPHLERVLGFVKVAKEQGAKVLCG<br>GDIYVPEDPKLKDGYYMRPCVLTNCRDDMTCVKEEIFGP<br>VMSILSFDTEAEVLERANDTTFGLAAGVFTRDIQRAHRVV<br>AELQAGTCFINNYNVSPVELPFGGYKKSGFGRENGRVTIE<br>YYSQLKTVCVEMGDVESAFLEHHHHHH	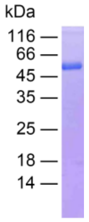	Group7<br>_batch3
Q7L9L4	MOB1B	1	216	MSFLFGSRSSKTFKPKKNIPEGSHQYELLKHAEATLGSGNL<br>RMAVMLPEGEDLNEWVAVNTVDFFNQINMLYGTITDFC<br>TEESCPVMSAGPKYEYHWADGTNIKKPIKCSAPKYIDYLM<br>TWVQDQLDDETLFPSKIGVPFPKNFMSVAKTILKRLFRVY<br>AHIYHQHFDPVIQLQEEAHLNTSFKHFIFFVQEFNLIDRRE<br>LAPLQELIEKLTSKDRLEHHHHHH	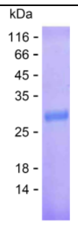	Group7<br>_batch3
Q9NX40	OCIAD1	1	245	MNGRADFREPNAEVPRPIPHIGPDYIPTEEERRVFAECND<br>ESFWFRSVPLAATSMLITQGLISKGILSSHPKYGSIPKLILAC<br>IMGYFAGKLSYVKTCQEKFKKLENSPLGEALRSGQARRSS<br>PPGHYYQKSKYDSSVSGQSSFVTSPAADNIEMLPHYEPIP<br>FSSSMNESAPTGITDHIVQGPDPNLEESPKRKNITYEELRN<br>KNRESYEVSLTQKTDPSVRPMHERVPKKEVKVNKYGDT<br>WDELEHHHHHH	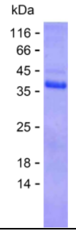	Group7<br>_batch3

Q8WVI7	PPP1R1C	1	109	MEPNSPKKIQFAVPVFQSQIAPEAAEQIRKRRPTPASLVILNEHNPPEIDDKRGPNTQGELQNASPKQRKQSVYTPPTIKGVKHLKGQNESAFPEEEEGTNEREEQRDHLEHHHHHH	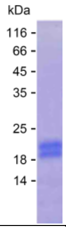	Group7_batch3
Q99417	MYCBP	1	103	MAHYKAADSKREQFRRYLEKSGVLDTLTKVLVALYEEPEKPNSALDFLKHHLGAATPENPEIELLRLELAEMKEKYEAIVEENKKLKAKLAQYEPPQEEKRAELEHHHHHH	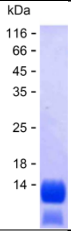	Group7_batch3
Q9UBK7	RABL2A	1	228	MAEDKTKPSELDQGKYDADDNVKIICLGDSAVGKSKLMERFLMDGFQPQQLSTYALTLYKHTATVDGKTILVDFWDTAGQERFQSMHASYYHKAHACIMVFDIQRKVTYRNLSTWYTELREFRPEIPCIVVANKIDDINVTQKSFNFAKKFSLPLYFVSAADGTNVVKLFNDAIRLAVSYKQNSQDFMDEIFQELENFSLEQEEEDVPDQEQSSSIETPSEEVASPHSLEHHHHHH	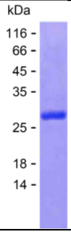	Group7_batch3
O43247	TEX33	1	280	MELGHGAGTTTFTRAHLNDKEGQQDLDPWKAAYSSLDTSKFKNQGLSSPQPLPLGASAQGSSLGQCHLKEIPPPPPTAASRDSLGMDPQSRSLKNAGSRSSSRENRATSGEGAQPCQGTDDGPSLGAQDQRSTPTNQKGSIIPNNIRHKFGSNVVDQLVSEEQAQKAIDEVFEGQKRASSWPSRTQNPVEISSVFSDYYDLGYNMRSNLFRGAAEETKSLMKASYTPEVIEKSVRDLEHWHGRKTDDLGRWHQKNAMNLNLQKALEEKYGENSKSKSSKYLEHHHHHH	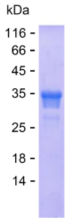	Group8_batch3
Q969P6	TOP1MT	51	601	MVKWRQLEHKGPYFAPPYEPLPDGVRFFYEGRPVRLSVAAEEVATFYGRMLDHEYTTKEVFRKNFFNDWRKEMAVEEREVIKSLDKCDFTEIHRYFVDKAAARKVLSREEKQKLKEEAEKLQQEFGYCILDGHQEKIGNFKIEPPGLFRGRGDHPKMGMLKRRITPEDVVINCSRDSKIPEPPAGHQWKEVRSDNTVTWLAAWTESVQNSIKYIMLNPCSKLKGETAWQKFETARRLRGFVDEIRSQYRADWKSREMKTRQRAVALYFIDKLALRAGNEKEDGEAADTVGCCSLRVEHVQLHPEADGCQHVVEFDFLGKDCIRYYNRVPVEKPVYKNLQLFMENKDPRDDLFDRLTTTSLNKHLQELMDGLTAKVFRTYNASITLQEQLRALTRAEDSIAAKILSYNRANRVVAILCNHQRATPSTFEKSMQNLQTKIQAKKEQVAEARAELRRARAEHKAQGDGKSRSVLEKKRRLLEKLQEQLAQLSVQATDKEENKQVALGTSKLNYLDPRISIAWCKRFRVPVEKIYSKTQRERFAWALAMAGEDFEFLEHHHHHH	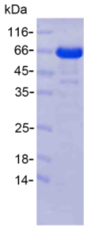	Group8_batch3
P30711	GSTT1	1	240	MGLELYLDLLSQPCRAVYIFAKKNDIPFELRIVDLIKGQHLSDAFAQVNPLKKVPALKDGDFTLTESVAILLYLTRKYKVPDYWYPQDLQARARVDEYLAWQHTTLRRSCLRALWHKVMFPVFLGEPVSPQTLAATLAELDVTLQLLEDKFLQNKAFLTGPHISLADLVAITELMHPVGAGCQVFEGRPKLATWRQRVEAAVGEDLFQEAHEVILKAKDFPPADPTIKQKLMPWVLAMIRLEHHHHHH	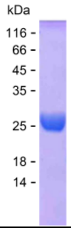	Group8_batch3
P61970	NUTF2	1	127	MGDKPIWEQIGSSFIQHYYQLFDNDRTQLGAIYIDASCLTWEGQQFQGKAAIVEKLSSLPFQKIQHSITAQDHQPTPDSCIISMVVGQLKADEDPIMGFHQMFLLKNINDAWVCTNDMFRLALHNFGLEHHHHHH	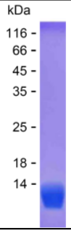	Group8_batch3

Q9NVA2	SEPTIN11	1	429	MAVAVGRPSNEELRNLSLSGHVGFDSLPDQLVNKSTSQGFCFNILCVGETGIGKSTLMDTLFNTKFESDPATHNEPGVRLKARSYELQESNVRLKLTIVDTVGFGDQINKDDSYKPIVEYIDAQFEAYLQEELKIKRSLFNYHDTRIHACLYFIAPTGHSLKSLDLVTMKKLDSKVNIIPIIAKADTIAKNELHKFKSKIMSELVSNGVQIYQFPTDEETVAEINATMSVHLPFAVVGSTEEVKIGNKMAKARQYPWGVVQVENENHCDFVKLREMLIRVNMEDLREQTHTRHYELYRRCKLEEMGFKDTDPDSKPFSLQETYEAKRNEFLGELQKKEEEMRQMFVMRVKEKEAELKEAEKELHEKFDLLKRTHQEEKKKVEDKKKELEEEVNNFQKKKAAAQLLQSQAQQSGAQQTKKDKDKKNASFTLEHHHHHH	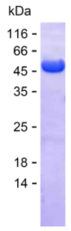	Group8 _batch3
Q9UBQ7	GRHPR	1	328	MRPVRLMKVFVTRRIPAEGRVALARAADCEVEQWDSDEPIPAKELERGVAGAHGLLCLLSDHVDKRILDAAGANLKVISTMSVGIDHLALDEIKKRGIRVGYTPDVLTDTTAELAVSLLLTTCRRLPEAIEEVKNGGWTSWKPLWLCGYGLTQSTVGIIGLGRIGQAIARRLKPFGVQRFLYTGRQPRPEEAAEFQAEFVSTPELAAQSDFIVVACSLTPATEGLCNKDFFQKMKETAVFINISRGDVVNQDDLYQALASGKIAAAGLDVTSPEPLPTNHPLLTLKNCVILPHIGSATHRTRNTMSLLAANNLLAGLRGEPMPSELKLEHHHHHH	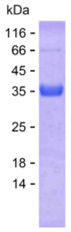	Group8 _batch3
Q9UM19	HPCAL4	1	191	MGKTNSKLAPEVLEDLVQNTEFSEQELKQWYKGFLKDCPSGILNLEEFQQLYIKFFPYGDASKFAQHAFRTFDKNGDGTIDFREFICALSVTSRGSFEQKLNWAFEMYDLDGDGRITRLEMLEIIEAIYKMVGTVIMMRMNQDGLTPQQRVDKIFKKMDQDKDDQITLEEFKEAAKSDPSIVLLLQCDMQKLEHHHHHH	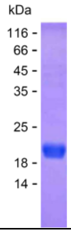	Group8 _batch3
O96001	PPP1R17	1	155	MMSTEQMQPLELSEDRLDKLDPRCSHLDDLSDQFIKDCDLKKKPRKGKNVQATLNVESDQKKPRRKDTPALHIPPFIPGVFSEHLIKRYDVQERHPKGKMIPVLHNTDLEQKKPRRKDTPALHMSPFAAGVTLLRDERPKAIVEDDEKDGDKIAILEHHHHHH	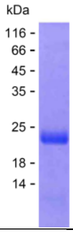	Group8 _batch3
O75348	ATP6V1G1	1	118	MASQSQGIQQLLQAEKRAAEKVSEARKRKNRRLKQAKEEAQAEIEQYRLQREKEFKAKEAAALGSRGSCSTEVEKETQEKMTILQTYFRQNRDEVLDNLLAFVCDIRPEIHENYRINGLEHHHHHH	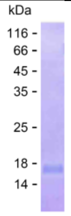	Group1 _batch4
Q9UKY7	CDV3	1	258	MAETEERSLDNFFAKRDKKKKKERSNRAASAAGAAGSAGGSSGAAGAAGGGAGAGTRPGDGGTASAGAAGPGAATKAVTKDEDEWKELEQKEVDYSGLRVQAMQISSEKEEDDNEKRQDPGDNWEEGGGGGGGMEKSSGPWNKTAPVQAPPAPVIVTETPEPAMTSGVYRPPGARLTTTRKTPQGPPEIYSDTQFPSLQSTAKHVESRKDKEMEKSFEVVRHKNRGRDEVSKNQALKLQLDNQYAVLENQKSSHSQYNLEHHHHHH	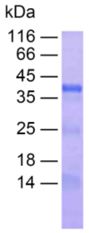	Group1 _batch4
Q9UBQ5	EIF3K	1	218	MAMFEQMRANVGKLLKGIDRYNPENLATLERYVETQAKENAYDLEANLAVLKLYQFNPAFFQTTVTAQILLKALTNLPHTDFTLCKCMIDQAHQEERPIRQILYLGDLLETCHFQAFWQALDENMDLLEGITGFEDSVRKFICHVVGITYQHIDRWLLAEMLGDLSDSQLKVWMSKYGWSADESGQIFICSQEESIKPKNIVEKIDFDSVSSIMASSQLEHHHHHH	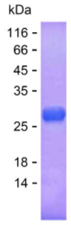	Group1 _batch4

O43687	AKAP7	1	104	MGQLCCFPFSRDEGKISELESSSSAVLQRYSKDIPSWSSG<br>EKNGGEPDDAELVRLSKRLVENAVLKAVQQYLEETQNK<br>NKPGEGSSVKTEAADQNGNDNENNRKLEHHHHHH	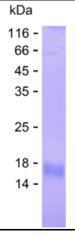	Group1<br>_batch4
P61201	COPS2	1	443	MSDMEDDFMCDDEEDYDLEYSEDSNSEPNVDLENQYY<br>NSKALKEDDPKAALSSFQKVLELEGEKGEWGFKALKQMI<br>KINFKLTNFPEMMNRYKQLLTYIRSAVTRNYSEKSINSILD<br>YISTSKQMDLLQEFYETTLEALKDAKNDRLWFKTNTKLGK<br>LYLEREEYGKLQKILRQLHQSCQTDDGEDDLKKGTQLLEI<br>YALEIQMYTAQKNNKKLKALYEQSLHIKSAIPHPLIMGVIR<br>ECGGKMHLREGEFEKAHTDFFEAFKNYDESGSPRRTTCLK<br>YLVLANMLMKSGINPFDSQEAKPYKNDPEILAMTNLVSA<br>YQNNDITEFEKILKTNHSNIMDDPFIREHIEELLRNIRTQVL<br>IKLIKPYTRIHIPFISKELNIDVADVESLLVQCILDNTIHGRID<br>QVNQLLELDHQKRGGARYTALDKWTNQLNSLNQAVVS<br>KLALEHHHHHH	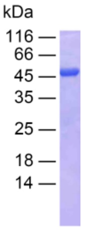	Group1<br>_batch4
P05204	HMGN2	1	90	MPKRKAEGDAKGDKAKVKDEPQRRSARLSAKPAPPKPEP<br>KPKKAPAKKGEKVPKGKKGKADAGKEGNNPAENGDAKT<br>DQAQKAEGAGDAKLEHHHHHH	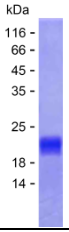	Group1<br>_batch4
Q16774	GUK1	1	197	MSGPRPVVLSGPSGAGKSTLLKRLLQEHSGIFGFSVSHTT<br>RNPRPGEENGKDYYFVTREVMQRDIAAGDFIEHAEFSGN<br>LYGTSKVAVQAVQAMNRICVLDVDLQGVRNIKATDLRPI<br>YISVQPPSLHVLEQRLRQRNTETEESLVKRLAAAQADMES<br>SKEPGLFDVVIINDSLDQAYAELKEALSEEIKKAQRTGALE<br>HHHHHH	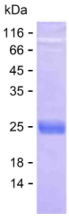	Group1<br>_batch4
Q13442	PDAP1	1	181	MPKGGRKGGHKGRARQYTSPEEIDAQLQAEKQKAREEE<br>EQKEGGDGAAGDPKKEKKSLDSDESEDEEDDYQQKRKG<br>VEGLIDIENPNRVAQTTKKVTQLDLDGPKELSRREREEIEK<br>QKAKERYMKMHLAGKTEQAKADLARLAIIRKQREEAARK<br>KEEERKAKDDATLSGKRMQSLSLNKLEHHHHHH	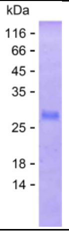	Group2<br>_batch4
O14908	GIPC1	1	333	MPLGLGRRKKAPPLVENEEAEPGRGGLGVGEPGPLGGG<br>GSGGPQMGLPPPPPALRPRLVFHTQLAHGSPTGRIEGFT<br>NVKELYGKIAEAFRLPTAEVMFCTLNTHKVDMDKLLGGQ<br>IGLEDFIFAHVKGQRKEVEVFKSEDALGLTITDNGAGYAFI<br>KRIKEGSVIDHIHLISVGDMIEAINGQSLLGCRHYEVARLLK<br>ELPRGRTFTLKLTEPRKAFDMISQRSAGGRPGSGPQLGTG<br>RGTLRLRSRGPATVEDLPSAFEEKAIEKVDDLLESYMGIRD<br>TELAATMVELGKDKRNPDELAEALDERLGDFAFPDEFVF<br>DVWGAIGDAKVGRYLEHHHHHH	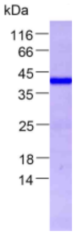	Group2<br>_batch4
P07320	CRYGD	1	174	MGKITLYEDRGFQGRHYECSSDHPNLQPYLSRCNSARV<br>DSGCWMLYEQPNYSGLQYFLRRGDYADHQQWMGLSD<br>SVRSCRLIPHSGSHRIRLYEREDYRGQMIEFTEDCSCLQDR<br>FRFNEIHSLNVLEGSWVLYELSNYRGRQYLLMPGDYRRY<br>QDWGATNARVGSLRRVIDFSLEHHHHHH	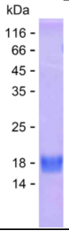	Group2<br>_batch4

Q02045	MYL5	1	173	MASRKTKKKEGGALRAQRASSNVFSNFEQTQIQEFKEAFTLMDQNRDGFIDKEDLKDTYASLGKTNVKDDELDAMLKEASGPINFTMFLNLFGEKLSGTDAEETILNAFKMLDPDGKGKINKEYIKRLLMSQADKMTAEEVDQMFQFASIDVAGNLDYKALSYVITHGEEKEELEHHHHHH	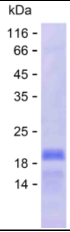	Group2 _batch4
P00326	ADH1C	1	375	MSTAGKVIKCKAAVLWELKKPFSIEEVEVAPPKAHEVRIKMVAAGICRSDEHVVSGNLVTPLPVILGHEAAGIVESVGEGVTTVKPGDKVIPLFTPQCGKCRICKNPESNYCLKNDLGNPRGTLQDGTRRFTCSGKPIHHFVGVSTFSQYTVVDENAVAKIDAASPLEKVCLIGCGFSTGYGSAVKVAKVTPGSTCAVFGLGGVGLSVVMGCKAAGAARIIAVDINKDKFAKAKELGATECINPQDYKKPIQEVLKEMTDGGVDFSFEVIGRLDTMMASLLCCHEACGTSVIVGVPPDSQNLSINPMLLLTGRTWKGAIFGGFKSKESVPKLVADFMAKKFSLDALITNILPFEKINEGFDLLRSGKSIRTVLTFLEHHHHHH	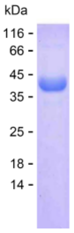	Group2 _batch4
P28222	HTR1B	229	315	MGRIYVEARSRILKQTPNRTGKRLTRAQLITDSPGSTSSVTSINSRVPDVPSESGSPVYVNQVKVRVSDALLEKKKLMAARERKATKTLEHHHHHH	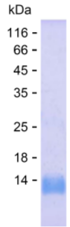	Group2 _batch4
P49721	PSMB2	1	201	MEYLIGIQGPDYVLVASDRVAASNIVQMKDDHDKMFKMSEKILLLCVGEAGDTVQFAEYIQKNVQLYKMRNGYELSPTAAANFTRRNLADCLRSRTPYHVNLLLAGYDEHEGPALYYMDYLAALAKAPFAAHGYGAFLTLSILDRYYTPTISRERAVELLRKCLEELQKRFILNLPTFSVRIIDKNGIHDLDNISFPKQGSLEHHHHHH	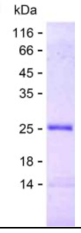	Group2 _batch4
Q9HAV7	GRPEL1	28	217	MCTATKQKNSGQNLEEDMGQSEQKADPPATEKTLLEEKVKLEEQLKETVEKYKRALADTENLRQRSQKLVEEAKLYGIQAFCKDLLEVADVLEKATQCVPKEEIKDDNPHLKNLYEGLVMTEVQIQKVFTKHGLLKLNPVGAKFDPYEHEALFHTPVEGKEPGTVALVSKVGYKLHGRTLRPALVGVVKEALEHHHHHH	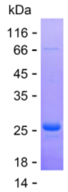	Group2 _batch4
O60749	SNX2	1	519	MAAEREPPPLGDGKPTDFEDLEDGEDLFTSTVSTLESSPSSPEPASLPAEDISANSNGPKPTEVVLDDDREDLFAEATEEVSLDSPEREPILSSEPSPAVTPVTPTTLIAPRIESKSMSAPVIFDRSREEIEEEANGDIFDIEIGVSDPEKVGDGMNAYMAYRVTTKTSLSMFSKSEFSVKRRFSDFLGLHSKLASKYLHVGYIVPPAPEKSIVGMTKVKVGKEDSSSTEFVEKRRAALERYLQRTVKHPTLLQDPDLRQFLESSELPRAVNTQALSGAGILRMVNKAADAVNKMTIKMNESDAWFEEKQQQFENLDQQLRKLHVSVEALVCHRKELSANTAAFAKSAAMLGNSEDHTALSRALSQLAEVEEKIDQLHQEQAFADFYMFSELLSDYIRLIAAVKGVFDHRMKCWQKWEDAQITLLKKREAEAKMMVANKPDKIQQAKNEIREWEAKVQQGERDFEQISKTIRKEVGRFEKERVKDFKTVIIKYLESLVQTQQQLIKYWEAFLPEAKAIALEHHHHHH	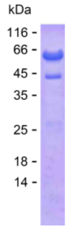	Group3 _batch4
O60925	PFDN1	1	122	MAAPVDLELKKAFTELQAKVIDTQQKVKLADIQIEQLNRTKKHAHLTDTEIMTLVDETNMYEGVGRMFILQSKEAIHSQLLEKQKIAEEKIKELEQKKSYLERSVKEAEDNIREMLMARRAQLEHHHHHH	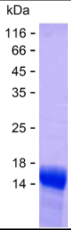	Group3 _batch4

Q9NV23	OLAH	1	265	MERGDQPKRTRNENIFNCLYKNPEATFKLICFPWMGGG<br>STHFAKWGQDTHDLLEVHSLRLPGRESRVEEPLENDISQL<br>VDEVVCALQPVIQDKPFAFFGHSMGSYIAFRTALGLKEN<br>NQPEPLHLFLSSATPVHSKAWHRIPKDDELSEEQISHYLM<br>EFGGTPKHFAEAKEFVKQCSPIIRADLNIVRSCTSNVPSKA<br>VLSCDLTCFVGSEDIAKDMEAWKDVTSGNAKIYQLPGG<br>HFYLLDPANEKLIKNYIIKCLEVSSISNFLEHHHHHH	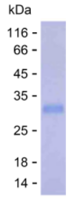	Group3<br>_batch4
Q7LBR1	CHMP1B	1	199	MSNMEKHLFNLKFAAKELSRSAKKCDKEEKAEKAKIKKAI<br>QKGNMEVARIHAENAIRQKNQAVNFLRMSARVDAVAA<br>RVQTAVTMGKVTKSMAGVVKSMDATLKTMNLEKISALM<br>DKFEHQFETLDVQTQQMEDTMSSTTTLTTPQNQVDML<br>LQEMADEAGLDLNMELPQGQTGSVGTSVASAEQDELS<br>QRLARLRDQVLEHHHHHH	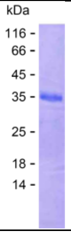	Group3<br>_batch4
Q9ULP0	NDRG4	1	352	MPECWDGEHDIETPYGLLHVVIRGSPKGNRPAILTYHDV<br>GLNHKLCFNTFFNFEDMQEITKHFVVCHVDAPGQQVGA<br>SQFPQGYQFPSMEQLAAMLPSVVQHFGFKYVIGIGVGA<br>GAYVLAKFALIFPDLVEGLVLVNIDPNGKGWIDWAATKLS<br>GLTSTLPDTVLSHLFSQEELVNNTELVQSYRQQIGNVVN<br>QANLQLFWNMYNSRRDLDINRPGTVPNAKTLRCPVML<br>VVGDNAPAEDGVVECNSKLDPTTTTFLKMADSGGLPQV<br>TQPGKLTEAFKYFLQGMGYIAYLKDRRLSGGAVPSASMT<br>RLARSRTASLTSASSVDGSRPQACTHSESSEGLGQVNHT<br>MEVSCLEHHHHHH	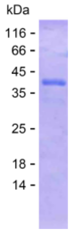	Group3<br>_batch4
O95989	NUDT3	1	172	MMKLKSNQTRTYDGDGYKKRAACLCFRSESEEEVLLVSS<br>SRHPDRWIVPGGGMEPEEEPSVAAVREVCEEAGVKGTLG<br>RLVGIFENQERKHRTYVYVLIVTEVLEDWEDSVNIGRKRE<br>WFKIEDAIKVLQYHKPVQASYFETLRQGYSANNGTPVVA<br>TTYSVSAQSSMSGIRLEHHHHHH	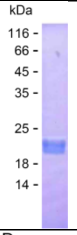	Group3<br>_batch4
Q9GZP8	C19orf33	1	106	MEFDLGAALEPTSQKPGVGAGHGGDPKLSPHKVQGRSE<br>AGAGPGPKQGHHSSSDSSSSSSDSDTDVKSHAAGSKQH<br>ESIPGKAKKPKVKKKEKGKKEKGKKKEAPHLEHHHHHH	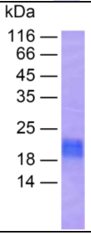	Group3<br>_batch4
P43357	MAGEA3	1	314	MPLEQRSQHCKPEEGLEARGEALGLVGAQAPATEEQEA<br>ASSSSTLVEVTLGEVPAAESPDPPQSPQGASSLPTTMNYP<br>LWSQSYEDSSNQEEEGPSTFPDLESEFQAALSRKVAELVH<br>FLLLKYRAREPVTKAEMLGSVVGNWQYFFPVIFSKASSSL<br>QLVFGIELMEVDPIGHLYIFATCLGLSYDGLLGDNQIMPK<br>AGLLIIVLAIIAREGDCAPEEKIWEELSVLEVFEGREDSILGD<br>PKKLLTQHFVQENYLEYRQVPGSDPACYEFLWGPRALVE<br>TSYVKVLHHMVKISGGPHISYPPLHEWVLREGEELEHHH<br>HHH	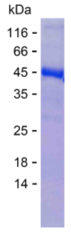	Group3<br>_batch4
P78318	IGBP1	1	339	MAAEDELQLPRLPELFETGRQLLDEVEVATEPAGSRIVQE<br>KVFKGLDLLEKAAEMLSQLDLFSRNEDLEEIASTDLKYLLV<br>PAFQGALTMKQVNPSKRLDHLQRAREHFINYLTQCHCY<br>HVAEFELPKTMNNSAENHTANSSMAYPSLVAMASQRQ<br>AKIQRYKQKKELEHRLSAMKSAVESGQADDERVREYYLL<br>HLQRWIDISLEEIESIDQEIKILRERDSSREASTSNSSRQERP<br>PVKPFILTRNMAQAKVFGAGYPSLPTMTVSDWYEQHRK<br>YGALPDQGIAKAAPEEFRKAAQQQEEQEEKEEEDDEQTL<br>HRAREWDDWKDTHPRGYGNRQNMGLEHHHHHH	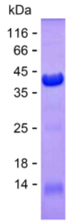	Group4<br>_batch4

Q99832	CCT7	1	543	MMPTPVILLKEGTDSSQGIPQLVSNISACQVIAEAVRTTLGPRGMDKLIVDGRGKATISNDGATILKLLDVVHPAAKTLVDIAKSQDAEVGDGTTSVTLLAAEFLKQVKPYVEEGLHPQIIIRAFRTATQLAVNKIKEIAVTVKKADKVEQRKLLEKCAMTALSSKLISQQKAFFAKMVVDAVMMLDDLLQLKMIGIKKVQGGALEDSQLVAGVAFKKTFSYAGFEMQPKKYHNPKIALLNVELELKAEKDNAEIRVHTVEDYQAIVDAEWNILYDKLEKIHHSGAKVVLSKLPIGDVATQYFADRDMFCAGRVPEEDLKRTMMACGGSIQTSVNALSADVLGRCQVFEETQIGGERYNFFTGCPKAKTCTFILRGGAEQFMEETERSLHDAIMIVRRAIKNDSVVAGGGAIEMELSKYLRDYSRTIPGKQQLLIGAYAKALEIIPRQLCDNAGFDATNILNKLRARHAQGGTWYGVDINNEDIADNFEAFVWEPAMVRINALTAASEAACLIVSVDETIKNPRSTVDAPTAAGRGRGRGRPHLEHHHHHH	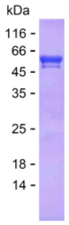	Group4 _batch4
Q5TDH0	DDI2	1	399	MLLTVYCVRRDLSEVTFSLQVDADFELHNFRALCELESGIPAAESQIVYAERPLTDNHRSLASYGLKDGDVVILRQKENADPRPPVQFPNLPRIDFSSIAVPGTSSPRQRQPPGTQQSHSSPGEITSSPQGLDNPALLRDMLLANPHELSLLKERNPPLAEALLSGDLEKFSRVLVEQQQDRARREQERIRLFSADPFDLEAQAKIEEDIRQQNIEENMTIAMEEAPESFGQVVMLYINCKVNGHPVKAFVDSGAQMTIMSQACAERCNIMRLVDRRWAGIAKGVGTQKIIGRVHLAQVQIEGDFLPCSFSILEEQPMDMLLGLDMLKRHQCSIDLKKNVLVIGTTGSQTTFLPEGELPECARLAYGAGREDVRPEEIADQELAEALQKSAEDAERQKPLEHHHHHH	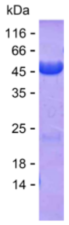	Group4 _batch4
P26641	EEF1G	1	437	MAAGTLYTYPENWRAFKALIAAQYSGAQVRVLSAPPHFHFGQTNRTPEFLRKFPAGKVPAFEGDDGFCVFESNAIAYYVSNEELRGSTPEAAAQVVQWVSFADSDIVPPASTWVFPTLGIMHHNKQATENAKEEVRRILGLLDAYLKTRTFLVGERVTLADITVVCTLLWLYKQVLEPSFRQAFPNTNRWFLTCINQPQFRAVLGEVKLCEKMAQFDAKKFAETQPKKDTPRKEKGSREEKQKPQAERKEEKKAAAPAPEEEMDECEQALAAEPKAKDPFAHLPKSTFVLDEFKRKYSNEDTLSVALPYFWEHFDKDGWSLWYSEYRFPEELTQTFMSCNLITGMFQRLDKLRKNAFASVILFGTNNSSSISGVWVFRGQELAFPLSPDWQVDYESYTWRKLDPGSEETQTLVREYFSWEGAFQHVGKAFNQGKIFKLEHHHHHH	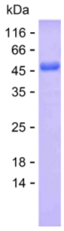	Group4 _batch4
Q99733	NAP1L4	1	375	MADHSFSDGVPSDSVEAAKNASNTEKLTDQVMQNPRVLAALQERLDNVPHTPSSYIETLPKAVKRRINALKQLQVRCAHIEAKFYEEVHDLERKYAALYQPLFDKRREFITGDVEPTDAESEWHSENEEEEKLAGDMKSKVVVTEKAAATAEEPDPKGIPEFWFTIFRNVDMLSELVQEYDEPILKHLQDIKVKFSDPGQPMSFVLEFHFEPNDYFTNSVLTKTYKMKSEPDKADPFSFEGPEIVDCDGCTIDWKKGKNVTVKTIKKKQKHKGRGTVRTITKQVPNESFFNFFNPLKASGDGESLDEDSEFTLASDFEIGHFFRERIVPRAVLYFTGEAIEDDDNFEEGEEGEEEELEGDEEGEDEDDAEINPKVLEHHHHHH	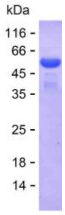	Group4 _batch4
Q15084	PDIA6	20	440	MLYSSSDDVIELTPSNFNREVIQSDSLWLVEFYAPWCGHCQRLTPEWKKAATALKDVVKVGAVDADKHHSLGGQYGVQGFPTIKIFGSNKNRPEDYQGGRTGEAIVDAALSALRQLVKDRLGGRSGGYSSGKQGRSDSSSKKDVIELTDDSFDKNVLDSEDVWMVEFYAPWCGHCKNLEPEWAAAASEVKEQTKGKVKLAAVDATVNQVLASRYGIRGFPTIKIFQKGESPVDYDGGRTRSDIVSRALDLFSDNAPPPELLEIINEDIAKRTCEEHQLCVVAVLPHILDTGAAGRNSYLEVLLKLADKYKKKMWGWLWTEAGAQSELETALGIGGFGYPAMAAINARKMKFALLKGSFSEQGINEFLRELSFGRGSTAPVGGGAFPTIVEREPWDGRDGELPVEDDIDLSDVELDDLGKDELEHHHHHH	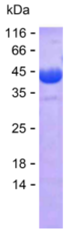	Group4 _batch4

Q9UJ70	NAGK	1	344	MAAIYGGVEGGGTRSEVLLVSEDGKILAEADGLSTNHWLIGTDKCVERINEMVNRAKRKAGVDPLVPLRSLGLSLSGGDQEDAGRILIEELRDRFPYLSESYLITTDAAGSIATATPDGGVVLISGTGSNCRLINPDGSESGCGGWGHMMGDEGSAYWIAHQAVKIVFDSIDNLEAAPHDIGYVKQAMFHYFQVPDRLGILTHLYRDFDKCRFAGFCRKIAEGAQQGDPLSRYIFRKAGEMLGRHIVAVLPEIDPVLFQGKIGLPILCVGSVWKSWELLKEGFLLALTQGREIQAQNFFSSFTLMKLRHSSALGGASLGARHIGHLLPMDYSANAIAFYSYTFSLEHHHHHH	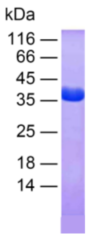	Group4_batch4
Q96D15	RCN3	21	328	MKPSPDAGPHGQGRVHQAAPLSDAPHDDAHGNFQYDHEAFLGREVAKEFDQLTPEESQARLGRIVDRMDRAGDGDGWVSLAELRAWIAHTQQRHIRDSVSAAWDTYDTDRDGRVGWEELRNATYGHYAPGEEFHDVEDAETYKKMLARDERRFRVADQDGDSMATREELTAFLHPEEFPHMRDIVIAETLEDLDRNKDGYVQVEEYIADLYSAEPGEEEPAWVQTERQQFRDFRDLNKDGHLDGSEVGHWVLPPAQDQPLVEANHLLHESDTDKDGRLSKAEILGNWNMFVGSQATNYGEDLTRHHDELEHHHHHH	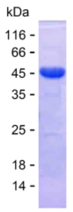	Group4_batch4
P45973	CBX5	1	191	MGKKTKRTADSSSSEDEEEYVVEKVLDRRVVKGQVEYLLKWKGFSEEHNTWEPEKNLDCPELISEFMKKYKKMKEGENNKPREKSESNKRKSNFSNSADDIKSKKKREQSNDIARGFERGLEPEKIIGATDSCGDLMFLMKWKDTDEADLVLAKEANVKCPQIVIAFYEERLTWHAYPEDAENKEKETAKSLEHHHHHH	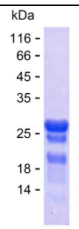	Group5_batch4
P20309	CHRM3	253	491	MRIYKETEKRTKELAGLQASGTEAETENFVHPTGSSRSCSSYELQQQSMKRSNRRKYGRCHFWFTTKSWKPSSEQMDQDHSSSDSWNNNDAAASLENSASSDEEDIGSETRAIYSIVLKLPGHSTILNSTKLPSSDNLQVPEEELGMVDLERKADKLQAQKSVDDGGSFPKSFSKLPIQLESAVDTAKTSDVNSSVGKSTATLPLSFKEATLAKRFALKTRSQITKRKRMSLVKEKKAAQLEHHHHHH	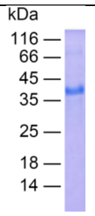	Group5_batch4
P62760	VSNL1	1	191	MGKQNSKLAPEVMEDLVKSTEFNEHELKQWYKGFLKDCPSGRLNLEEFQQLYVKFFPYGDASKFAQHAFRTFDKNGDGTIDFREFICALSITSRGSFEQKLNWAFNMYDLDGDGKITRVEMLEIIEAIYKMVGTVIMMKMNEDGLTPEQRVDKIFSKMDKNKDDQITLDEFKEAAKSDPSIVLLLQCDIQKLEHHHHHH	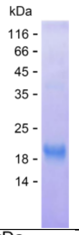	Group5_batch4
P11229	CHRM1	210	366	MRIYRETENRARELAALQGSETPGKGGGSSSSSERSQPGAEGSPETPPGRCCRCCRAPRLLQAYSWKEEEEEDEGSMESLTSSEGEEPGSEVVIKMPMVDPEAQAPTKQPPRSSPNTVKRPTKKGRDRAGKGQKPRGKEQLAKRKTFSLVKEKKAARTLEHHHHHH	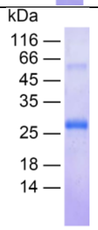	Group5_batch4
A9UHW6	MIF4GD	1	222	MGEPSREEYKIQSFDAETQQLLKTALKDPGAVDLEKVANVIVDHSLQDCVFSKEAGRMCYAIIQAESKQAGQSVFRRGLLNRLQQEYQAREQLRARSLQGWVCYVTFICNIFDYLRVNNMPMMALVNPVYDCLFRLAQPDSLSKEEEVDCLVLQLHRVGEQLEKMNGQRMDELFVLIRDGFLLPTGLSSLAQLLLLEIIEFRAAGWKTTPAAHKYYYSEVSDLEHHHHHH	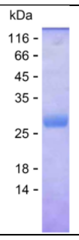	Group5_batch4

P48507	GCLM	1	274	MGTDSRAAKALLARARTLHLQTGNLLNWGRLRKKCPSTHSEELHDCIQKTLNEWSSQINPDLVREFPDVLECTVSHAVEKINPDEREEMKVSAKLFIVESNSSSSTRSAVDMACSVLGVAQLDSVIIASPPIEDGVNLSLEHLQPYWEELENLVQSKKIVAIGTSDLDKTQLEQLYQWAQVKPNSNQVNLASCCVMPPDLTAFAKQFDIQLLTHNDPKELLSEASFQEALQESIPDIQAHEWVPLWLLRYSVIVKSRGIIKSKGYILQAKRRGSLEHHHHHH	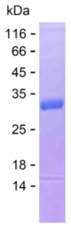	Group5_batch4
Q8WWF6	DNAJB3	1	145	MVDYYEVLDVPRQASSEAIKKAYRKLALKWHPDKNPENKEEAERRFKQVAEAYEVLSDAKKRDIYDRYGEAGAEGGCTGGRPFEDPFEYVFSFRDPADVFREFFGGQDPFSFDLLGNPLENILGGSEELLGKQKQSVCTPFLCLQLEHHHHHH	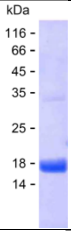	Group5_batch4
Q07817	BCL2L1	1	209	MSQSNRELVVDFLSYKLSQKGYSWSQFSDVEENRTEAPEGTESEMETPSAINGNPSWHLADSPAVNGATGHSSSLDAREVIPMAAVKQALREAGDEFELRYRRAFSDLTSQLHITPGTAYQSFEQVVNELFRDGVNWGRIVAFFSFGGALCVESVDKEMQVLVSRIAAWMATYLNDHLEPWIQENGGWDTFVELYGNNAAAESRKGQERLEHHHHHH	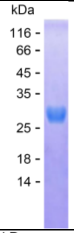	Group5_batch4
Q9BVG4	PBDC1	1	233	MAATSGTDEPVSGELVSVAHALSLPAESYGNDPDIEMAWAMRAMQHAEVYYKLISSVDPQFLKLTKVDDQIYSEFRKNFETLRIDVLDPEELKSESAKEKWRPFCLKFNGIVEDFNYGTLLRLDCSQGYTEENTIFAPRIQFFAIEIARNREGYNKAVYISVQDKEGEKGVNNGGEKRADSGEEENTKNGGEKGADSGEEKEEGINREDKTDKGGEKGKEADKEINKSGEKAMLEHHHHHH	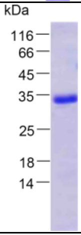	Group6_batch4
O95749	GGPS1	1	300	MEKTQETVQRILLEPYKYLLQLPGKQVRTKLSQAFNHWLKVPEDKLQIIIEVTEMLHNASLLIDDIEDNSKLRRGFPVAHSIYGIPSVINSANYVYFLGLEKVLTLDHPDAVKLFTRQLLELHQGQGLDIYWRDNYTCPTEEEYKAMVLQKTGGLFGLAVGLMQLFSDYKEDLKPLLNTLGLFFQIRDDYANLHSKEYSENKSFCEDLTEGKFSFPTIHAIWSRPESTQVQNILRQRTENIDIKKYCVHYLEDVGSFEYTRNTLKELEAKAYKQIDARGGNPELVALVKHLSKMFKEENELEHHHHHH	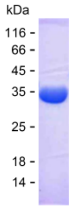	Group6_batch4
P36551	CPOX	111	454	MTSLGRPPEEEEDELAHRCSSFMAPPVTDLGELRRRPGDMKTKMELLILETQAQVCQALAQVDGGANFSVDRWERKEGGGGISCVLQDGCVFEKAGVSISVVHGNLSEEAAKQMRSRGKVLKTKDGKLPFCAMGVSSVIHPKNPHAPTIHFNYRYFEVEEADGNKQWWFGGGCDLTPTYLNQEDAVHFHRTLKEACDQHGPDLYPKFKKWCDDYFFIAHRGERRGIGGIFFDDLDSPSKEEVFRFVQSCARAVVPSYIPLVKKHCDDSFTPQEKLWQQLRRGRYVEFNLLYDRGTKFGLFTPGSRIESILMSLPLTARWEYMHSPSENSKEAEILEVLRHPRDWVRLEHHHHHH	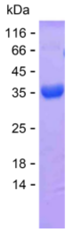	Group6_batch4
Q92890	UFD1	1	307	MFSFNMFDHPIPRVFQNRFSTQYRCFSVSMLAGPNDRSDVEKGGKIIMPPSALDQLSRLNITYPMLFKLTNKNSDRMTHCGVLEFVADEGICYLPHWMMQNLLLEEGGLVQVESVNLQVATYSKFQPQSPDFLDITNPKAVLENALRNFACLTTGDVIAINYNEKIYELRVMETKPDKAVSIIECDMNVDFDAPLGYKEPERQVQHEESTEGEADHSGYAGELGFRAFSGSGNRLDGKKKGVEPSPSPIKPGDIKRGIPNYEFKLGKITFIRNSRPLVKKVEEDEAGGRFVAFSGEGQSLRKKGRKPLEHHHHHH	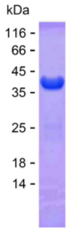	Group6_batch4

Q9H074	PAIP1	1	479	MSDGFDRAPGAGRGRSRGLGRGGGGPEGGGFPNGAGP<br>AERARHQPPQPKAPGFLQPPPLRQPRTTPPPGAQCEVP<br>ASPQRPSRPGALPEQTRPLRAPPSSQDKIPQQNSESAMA<br>KPQVVVAPVLMSKLSVNAPEFYPSGYSSSYTESYEDGCED<br>YPTLSEYVQDFLNHLTEQPGSFETEIEQFAETLNGCVTTD<br>DALQELVELIYQQATSIPNFSYMGARLCNYLSHHLTISPQ<br>SGNFRQLLLQRCRTEYEVKDQAAKGDEVTRKRFHAFVLF<br>LGELYLNLEIKGTNGQVTRADILQVGLRELLNALFSNPMD<br>DNLICAVKLLKLTGSVLEDAWKEKGKMDMEEIIQRIENVV<br>LDANCSRDVKQMLLKLVELRSSNWGRVHATSTYREATPE<br>NDPNYFMNEPTFYTSDGVPFTAADPDYQEKYQELLERED<br>FFPDYEENGTDLSGAGDPYLDDIDDEMDPEIEEAYEKFCL<br>ESERKRKQLEHHHHHH	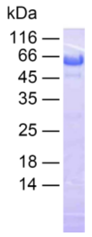	Group6<br>_batch4
P45985	MAP2K4	1	399	MAAPSPSGGGGSGGGSGSGTPGPVGSPAPGHPAVSSM<br>QGKRKALKLNFANPPFKSTARFTLNPNPTGVQNPHIERL<br>RTHSIESSGKLKISPEQHWDFTAEDLKDLGEIGRGAYGSV<br>NKMVHKPSGQIMAVKRIRSTVDEKEQKQLLMDLDVVMR<br>SSDCPYIVQFYGALFREGDCWICMELMSTSFDKFYKYVYS<br>VLDDVIPEEILGKITLATVKALNHLKENLKIIHRDIKPSNILLD<br>RSGNIKLCDFGISGQLVDSIAKTRDAGCRPYMAPERIDPS<br>ASRQGYDVRSDVWSLGITLYELATGRFPYPKWNSVFDQL<br>TQVVKGDPPQLSNSEEREFSPSFINFVNLCLTKDESKRPKY<br>KELLKHPFILMYEERAVEVACYVCKILDQMPATPSSPMYV<br>DLEHHHHHH	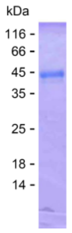	Group6<br>_batch4
Q9HC36	MRM3	41	420	MHHHHHHVFPSGEVVEQKRAPGKQPRKAPSEASAQEQ<br>REKQPLEESASRAPSTWEESGLRYDKAYPGDRRLSSVMTI<br>VKSRPFREKQGKILLEGRRLISDALKAGAVPKMFFFSRLEYL<br>KELPVDKLKGVSLIKVKFEDIKDWSDLVTPQGIMGIFAKPD<br>HVKMTYPKTQLQHSLPLLLICDNLRDPGNLGTILRSAAGA<br>GCSKVLLTKGCVDAWEPKVLRAGMGAHFRMPIINNLEW<br>ETVPNYLPPDTRVYVADNCGLYAQAEMSNKASDHGWV<br>CDQRVMKFHKYEEEEDVETGASQDWLPHVEVQSYDSD<br>WTEAPAAVVIGGETYGVSLESLQLAESTGGKRLLIPVVPG<br>VDSLNSAMAASILLFEGKRQLRGRAEDLSRDRSYH	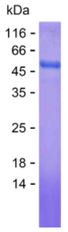	Group6<br>_batch4
Q8N7F7	UBL4B	1	174	MFLTVKLLLGQRCSLKVSGGQESVATLKRLVSRRLKVPEEQ<br>QHLLFRGQLLEDDKHLSDYCIGPNASINVIMQPLEKMAL<br>KEAHQPQTQPLWHQLGLVLAKHFEPQDAKAVLQLLRQE<br>HEERLQKISLEHLEQLAQYLLAEEPHVEPAGERELEAKARP<br>QSSCDMEEKEEAAADQLEHHHHHH	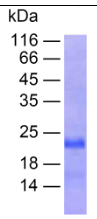	Group6<br>_batch4
P30040	ERP29	33	261	MLHTKGALPLDTVTFYKVIPKSKFVLVKFDTQYPYGEKQD<br>EFKRLAENSASSDDLLVAEVGISDYGDKLNMELSEKYKLD<br>KESYPVFYLFRDGDFENPVPYTGAVKVGAIQRWLKGQGV<br>YLGMPGCLPVYDALAGEFIRASGVEARQALLKQGQDNL<br>SSVKETQKKWAEQYLKIMGKILDQGEDFPASEMTRIARLI<br>EKNKMSDGKKEELQKSLNILTAFQKKGAEKEELEHHHHH<br>H	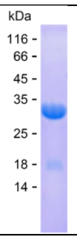	Group7<br>_batch4
P21728	DRD1	338	446	MRKAFSTLLGCYRLCPATNNAIETVSINNNGAAMFSSHH<br>EPRGSISKECNLVYLIPHAVGSSEDLKKEEAAGIARPLEKLS<br>PALSVILDYDTDVSLEKIQPITQNGQHPTLEHHHHHH	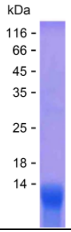	Group7<br>_batch4

O43423	ANP32C	1	234	MEMGRRIHSELRNRAPSDVKELALDNSRSNEGKLEALTD<br>EFEELEFLSKINGGLTSISDLPKLKLRKLELRVSGGLEVLAEK<br>CPNLTHLYLSGNKIKDLSTIEPLKQLENLKSLDLFNCEVTNL<br>NDYGENVFKLLLQLTYLDSCYWDHKEAPYSDIEDHVEGL<br>DDEEEGEHEEEYDEDAQVVEDEEGEEEEEEGEEEDVSGG<br>DEEDEEGYNDGEVDGEEDEEELGEEERGQKRKLEHHHH<br>HH	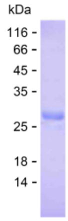	Group7<br>_batch4
O60784	TOM1	1	492	MDFLLGNPFSSPVGQRIEKATDGSLQSEDWALNMEICDII<br>NETEEGPKDALRAVKKRIVGNKNFHEVMLALTVLETCVK<br>NCGHRFHVLVASQDFVESVLVRTILPKNNPPTIVHDKVL<br>NLIQSWADAFRSSPDLTGVVTIYEDLRRKGLEFPMTDLD<br>MLSPIHTPQRTVFNSETQSGQDSVGTDSSQQEDSGQHA<br>APLPAPPILSGDTPIAPTPEQIGKLRSELEMVSGNVRVMSE<br>MLTELVPTQAEPADLELLQELNRTCRAMQQRVLELIPQIA<br>NEQLTEELLIVNDNLNNVFLRHERFERFRTGQTTKAPSEA<br>EPAADLIDMGPDPAATGNLSSQLAGMNLGSSSVRAGLQ<br>SLEASGRLEDEFDMFALTRGSSLADQRKEVKYEAPQATD<br>GLAGALDARQQSTGAIPVTQACLMEDIEQWLSTDVGND<br>AEEPKGVTSEEFDKFLEERAKAADRLPNLSSPSAEGPPGPP<br>SGPAPRKKTQEKDDDMLFALEHHHHHH	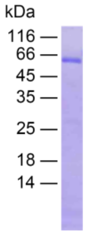	Group7<br>_batch4
Q00266	MAT1A	1	395	MNGPVDGLCDHSLSEGVFMFTSESVGEGHPDKICDQIS<br>DAVLDAHLKQDPNAKVACETVCKTGMVLLCGEITSMAM<br>VDYQRVVRDTIKHIGYDDSAKGFDFKTCNVLVALEQQSP<br>DIAQCVHLDRNEEDVGAGDQGLMFGYATDETEECMPLT<br>IILAHKLNARMADLRRSGLLPWLRPDSKTQVTVQYMQD<br>NGAVIPVRIHTIVISVQHNEDITLEEMRRALKEQVIRAVVP<br>AKYLDEDTVYHLQPSGRFVIGGPQGDAGVTGRKIIVDTY<br>GGWGAHGGGAFSGKDYTKVDRSAAYAARWVAKSLVKA<br>GLCRRVLVQVSYAIGVAEPLSISIFTYGTSQKTERELLDVVH<br>KNFDLRPGVIVRDLDLKKPIYQKTACYGHFGRSEFPWEVP<br>RKLVFLEHHHHHH	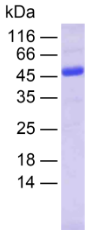	Group7<br>_batch4
Q9BRQ3	NUDT22	1	303	MDPEVTLLLQCPGGGLPQEQIQAELSPAHDRRPLPGGDE<br>AITAIWETRLKAQPWLFDAPKFRLHSATLAPIGSRGPQLLL<br>RLGLTSYRDFLGTNWSSSAAWLRQQGATDWGDTQAYL<br>ADPLGVGAALATADDFLVFLRRSRQVAEAPGLVDVPGG<br>HPEPQALCPGGSPQHQDLAGQLVVHELFSSVLQEICDEV<br>NLPLLTLSQPLLLGIARNETSAGRASAEFYVQCSLTSEQVR<br>KHYLSGGPEAHESTGIFFVETQNVQRLLETEMWAELCPS<br>AKGAIILYNRVQGSPTGAALGSPALLPPLEHHHHHH	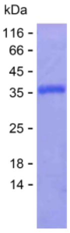	Group7<br>_batch4
Q15056	EIF4H	1	248	MADFDTYDDRAYSSFGGGRGSRGSAGGHGSRSQKELPT<br>EPPYTAYVGNLPFNTVQGDIDAIFKDLSIRSVRLVRDKDT<br>DKFKGFCYVEFDEVDSLKEALTYDGALLGDRSLRVDIAEG<br>RKQDKGGFGFRKGGPDDRGMGSSRESRGGWDSRDDF<br>NSGFRDDFLGGRGGSRPGDRRTGPPMGSRFRDGPPLRG<br>SNMDFREPTEEERAQRPRLQLKPRTVATPLNQVANPNS<br>AIFGGARPREEVVQKEQELEHHHHHH	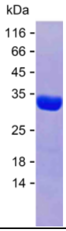	Group7<br>_batch4
Q15274	QPRT	1	297	MDAEGLALLLPPVTLAALVDSWLREDCPGLNYAALVSGA<br>GPSQAALWAKSPGVLAGQPFFDAIFTQLNCQVSWFLPE<br>GSKLVPVARVAEVRGPAHCLLLGERVALNTLARCSGIASA<br>AAAAVEAARGAGWTGHVAGTRKTTPGFRLVEKYGLLVG<br>GAASHRYDLGGLVMVKDNHVVAAGGVEKAVRAARQA<br>ADFTLKVEVECSSLQEAVQAAEAGADLVLLDNFKPEELHP<br>TATVLKAQFPSVAVEASGGITLDNLPQFCGPHIDVISMG<br>MLTQAAPALDFSLKLFAKEVAPVPKIHLEHHHHHH	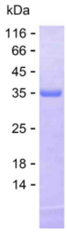	Group7<br>_batch4

Q99961	SH3GL1	1	368	MSVAGLKKQFYKASQLVSEKVGGAEGTKLDDDFKEMEK<br>KVDVTSKAVTEVLARTIEYLQPNPASRAKLTMLNTVSKIR<br>GQVKNPGYPQSEGLLGECMIRHGKELGGESNFGDALLD<br>AGESMKRLAEVKDSLDIEVKQNFIDPLQNLCEKDLKEIQH<br>HLKKLEGRRLDFDYKKKRQGKIPDEELRQALEKFEESKEVA<br>ETSMHNLLETDIEQVSQLSALVDAQLDYHRQAVQILDEL<br>AEKLKRRMREASSRPKREYKPKPREPFDLGEPEQSNGGFP<br>CTTAPKIAASSSFRSSDKPIRTPSRSMPPLDQPSCKALYDF<br>EPENDGELGFHEGDVITLTNQIDENWYEGMLDGQSGFF<br>PLSYVEVLVPLPQLEHHHHHH	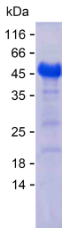	Group8<br>_batch4
O14879	IFIT3	1	490	MSEVTKNSLEKILPQLKCHFTWNLFKEDSVSRDLEDRVC<br>NQIEFLNTEFKATMYNLLAYIKHLDGNNEAALECLRQAEE<br>LIQQEHADQAEIRSLVTWGNYAWVYYHLGRLSDAQIYV<br>DKVKQTCKKFSNPYSIEYSELDCEEGWTQLKCGRNERAK<br>VCFEKALEEKPNNPEFSSGLAIAMYHLDNHPEKQFSTDVL<br>KQAIELSPDNQYVKVLLGLKLQKMNKEAEGEQFVEEALE<br>KSPCQTDVLRSAAKFYRRKGDLDKAIELFQRVLESTPNNG<br>YLYHQIGCCYKAKVRQMQNTGESEASGNKEMIEALKQY<br>AMDYSNKALEKGLNPLNAYSDLAEFLETECYQTPFNKEV<br>PDAEKQQSHQRYCNLQKYNGKSEDTAVQHGLEGLSISK<br>KSTDKEEIKDQPQNVSENLLPQNAPNYWYLQGLIHKQN<br>GDLLQAAKCYEKELGRLLRDAPSGIGSIFLSASELEDGSEE<br>MGQGAVSSSPRELLSNSEQLNLEHHHHHH	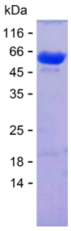	Group8<br>_batch4
P00325	ADH1B	1	375	MSTAGKVIKCKAAVLWEVKKPFSIEDVEVAPPKAYEVRIK<br>MVAVGICRTDDHVVSGNLVTPLPVILGHEAAGIVESVGE<br>GVTTVKPGDKVIPLFTPQCGKCRVCKNPESNYCLKNDLG<br>NPRGTLQDGTRRFTCRGKPIHHFLGTSTFSQYTVVDENA<br>VAKIDAASPLEKVCLIGCGFSTGYGSAVNVAKVTPGSTCA<br>VFGLGGVGLSAVMGCKAAGAARIIAVDINKDKFAKAKEL<br>GATECINPQDYKKPIQEVLKEMTDGGVDFSFEVIGRLDT<br>MMASLLCCHEACGTSVIVGVPPASQNLSINPMLLLTGRT<br>WKGAVYGGFKSKEGIPKLVADFMAKKFSLDALITHVLPFE<br>KINEGFDLLHSGKSIRTVLTFLEHHHHHH	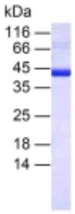	Group8<br>_batch4
P54920	NAPA	1	295	MDNSGKEAEAMALLAEAERKVKNSQSFFSGLFGGSSKIE<br>EACEIYARAANMFKMAKNWSAAGNAFCQAAQLHLQL<br>QSKHDAATCFVDAGNAFKKADPQEAINCLMRAIEIYTD<br>MGRFTIAAKHHISIAEIYETELVDIEKAIAHYEQSADYYKGE<br>ESNSSANKCLLKVAGYAALLEQYQKAIDIYEQVGTNAMD<br>SPLLKYSAKDYFFKAALCHFCIDMLNAKLAVQKYEELFPAF<br>SDSRECKLMKKLLEAHEEQNVDSYTESVKEYDSISRLDQ<br>WLTTMLLRIKKTIQGDEEDLRLEHHHHHH	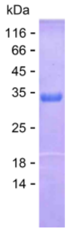	Group8<br>_batch4
Q75LS8	FKBP9P1	1	142	MDMGLREMCVGEKRTVIIPPHLGYGEAGVDGEVPGSAV<br>LVFDIELLELVAGLPEGYMFIWNGEVSPNLFEEIDKDGNGE<br>VLLEEFSEYIHAQVASGKGKLAPGFDAELIVKNMFTNQDR<br>NGDGKVTAEEFKLKDQEAKQDELEHHHHHH	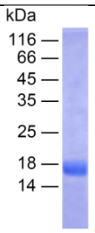	Group8<br>_batch4
P40261	NNMT	1	264	MESGFTSKDTYLSHFNPRDYLEKYYKFGSRHSAESQILKHL<br>LKNLFKIFCLDGVKGDLLIDIGSGPTIYQLLSACESFKEIVVT<br>DYSDQNLQELEKWLKKEPEAFDWSPVVTYVCDLEGNRV<br>KGPEKEEKLRQAVKQVLKCDVTQSQPLGAVPLPPADCVL<br>STLCLDAACPDLPTYCRALRNLGSLLKPGGFLVIMDALKS<br>SYYMIGEQKFSSLPLGREAVEAAVKEAGYTIEWFEVISQSY<br>SSTMANNEGLFSLVARKLSRPLEHHHHHH	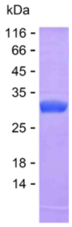	Group8<br>_batch4

Q6IS14	EIF5AL1	1	154	MADDLDFETGDAGASATFPMQCSALRKNGFVVLKGWPCKIVEMSASKTGKHGHAKVHLVGIDIFTGKKYEDICPSTHNMDVPNIKRNDFQLIGIQDGYLSLLQDSGEVPEDLRLPEGDLGKEIEQKYDCGEEILITVLSAMTEEAAVAIKAMAKLEHHHHHH	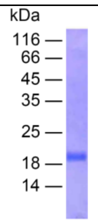	Group8 _batch4
Q6ZQV5	ZNF788P	1	82	MRNMIPQDNENPPQQGEANQNDSVAFEDVAVNFTPDEWALLDPSQKNLYREVMQETLRNLASIEVLWKRDSLKVKVISMEKFLEHHHHHH	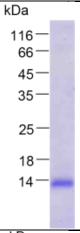	Group8 _batch4
P07311	ACYP1	1	99	MAEGNTLISVDYEIFGKVQGVFFRKHTQAEGKKLGLVGWVQNTDRGTVQGQLQGPISKVRHMQEWLETRGSPKSHIDKANFNNEKVILKLDYSDFQIVKLEHHHHHH	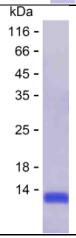	Group8 _batch2
P08913	ADRA2A	218	374	MRIYQIAKRRTRVPPSRRGPDAVAAPPGGTERRPNGLGPERSAGPGGAEAEPLPTQLNGAPGEPAPAGPRDTDALDLEESSSSDHAERPPGPRRPERGPRGKGKARASQVKPGDSLPRRGPGATGIGTPAAGPGEERVGAAKASRWRGRQNREKRFTFLEHHHHHH	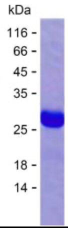	Group2 _batch2
P21549	AGXT	1	392	MASHKLLVTPPKALLKPLSIPNQLLLGPGPSNLPPRIMAAGGLQMIGSMSKDMYQIMDEIKEGIQYVFQTRNPLTLVISGSGHCALEAALVNVLEPGDSFLVGANGIWGQRAVDIGERIGARVHPMTKDPGGHYTLQEVEEGLAQHKPVLLFLTHGESSTGVLQPLDGFGELCHRYKCLLLVDSVASLGGTPLYMDRQGIDILYSGSQKALNAPPGTSLISFSDKAKKKMYSRKTKPFSFYLDIKWLANFWGCDDQPRMYHHTIPVISLYSLRESLALIAEQGLENSWRQHREAAAYLHGRLQALGLQLFVKDPALRLPTVTTVAVPAGYDWRDIVSYVIDHFDIEIMGGLGPSTGKVLRIGLLGCNATRENVDRVTEALRAALQHCPKKKLEHHHHHH	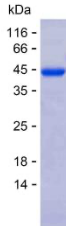	Group6 _batch2
P84085	ARF5	1	180	MGLTVSALFSRIFGKKQMRILMVGLDAAGKTTILYKLKLGEIVTTIPTIGFNVETVEYKNICFTVWDVGGQDKIRPLWRHYFQNTQGLIFVVDSNDRERVQESADELQKMLQEDELRDAVLLVFANKQDMPNAMPVSELTDKLGLQHLRSRTWYVQATCATQGTGLYDGLDWLSHELSKRLEHHHHHH	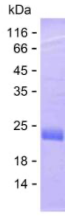	Group4 _batch2
P52566	ARHGDIB	1	201	MTEKAPEPHVEEDDDDELDSKLNYKPPPQKSLKELQEMDKDDESLIKYKKTLLGDGPVVTDPKAPNVVVTRLTLVCESAPGPITMDLTGDLEALKKETIVLKEGSEYRVKIHFKVNRDIVSGLKYVQHTYRTGVKVDKATFMVGSYGPRPEEYEFLTPVEEAPKGMLARGTYHNKSFFTDDDKQDHLSWEWNLSIKKEWTELEHHHHHH	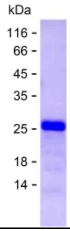	Group5 _batch2

Q7L266	ASRGL1	1	308	MNPIVVVHGGGAGPISKDRKERVHQGMVRAATVGYGIL<br>REGGSAVDAVEGAVVALEDDPEFNAGCGSVLNTNGEVE<br>MDASIMDGKDLSAGAVSAVQCIANPIKLARLVMEKTPH<br>CFLTDQGAAQFAAAMGVPEIPGEKLVTERNKKRLEKEKH<br>EKGAQKTDCQKNLGTVGAVALDCKGNVAYATSTGGIVN<br>KMVGRVGDSPCLGAGGYADNDIGAVSTTGHGESILKVN<br>LARLTLFHIEQGKTVEEAADLSLGYMKSRVKGLGGLIVVSK<br>TGDWVAKWTSTSMPWAAAKDGKLHFGIDPDDTTITDLP<br>LEHHHHHH	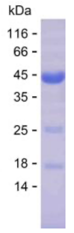	Group8<br>_batch2
Q8N8Y2	ATP6V0D2	1	350	MLEGAELYFNVDHGYLEGLVRGCKASLLTQQDYINLVQC<br>ETLEDLKIHLQTTDYGNFLANHTNPLTVSKIDTEMRKRLC<br>GEFEYFRNHSLEPLSTFLTYMTCSYMIDNVILLMNGALQK<br>KSVKEILGKCHPLGRFTEMEAVNIAETPSDLFNAILIETPLA<br>PFFQDCMSENALDELNIELLRNKLYKSYLEAFYKFCKNHG<br>DVTAEVMCPILEFEADRRAFIITLNSFGTELSKEDRETLYPT<br>FGKLYPEGLRLLAQAEDFDQMKNVADHYGVYKPLFEAV<br>GGSGGKTLEDVFYEREVQMNVLAFNRQFHYGVFYAYVK<br>LKEQEIRNIVWIAECISQRHRTKINSYIPILEHHHHHH	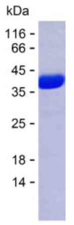	Group3<br>_batch2
Q16520	BATF	1	125	MPHSSDSSDSSFSRSPPPGKQDSSDDVRRVQRREKNRIA<br>AQKSRQRQTQKADTLHLESEDLEKQNAALRKEIKQLTEEL<br>KYFTSVLNSHEPLCSVLAASTPSPPEVVYSAHAFHQPHVS<br>SPRFQPLEHHHHHH	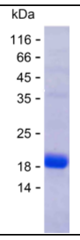	Group7<br>_batch2
P54687	BCAT1	1	386	MKDCSNGCSAECTGEGGSKEVVGTFKAKDLIVTPATILKE<br>KPDPNNLVFGTVFTDHMLTVEWSSEFGWEKPHIKPLQNL<br>SLHPGSSALHYAVELFEGLKAFRGVDNKIRLFQPNLNMD<br>RMYRSAVRATLPVFDKEELLECIQQLVKLDQEWVPYSTSA<br>SLYIRPTFIGTEPSLGVKKPTKALLFVLLSPVGPYFSSGTFNP<br>VSLWANPKYVRAWKGGTGDCKMGGNYGSSLFAQCEA<br>VDNGCQQVLWLYGEDHQITEVGTMNLFLYWINEDGEEE<br>LATPPLDGIILPGVTRRCILDLAHQWGEFKVSERYLTMDD<br>LTTALEGNRVREMFGSGTACVVCPVSDILYKGETIHIPTM<br>ENGPKLASRILSKLTDIQYGREESDWTIVLSLEHHHHHH	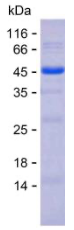	Group6<br>_batch2
O95861	BPNT1	1	308	MASSNTVLMRLVASAYSIAQKAGMIVRRVIAEGDLGIVEK<br>TCATDLQTKADRLAQMSICSSLARKFPKLTIIGEEDLPSEE<br>VDQELIEDSQWEEILKQPCPSQYSAIKEEDLVVWVDPLD<br>GTKEYTEGLLDNVTVLIGIAYEGKAIAGVINQPYYNYEAGP<br>DAVLGRTIWGVLGLGAFGFQLKEVPAGKHIITTTRSHSNK<br>LVTDCVAAMNPDAVLRVGGAGNKIIQLIEGKASAYVFAS<br>PGCKKWDTCAPEVILHAVGGKLTDIHGNVLQYHKDVKH<br>MNSAGVLATLRNYDYYASRVPESIKNALVPLEHHHHHH	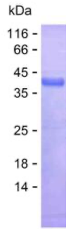	Group3<br>_batch2
Q14012	CAMK1	1	370	MLGAVEGPRWKQAEDIRDIYDFRDVLGTGAFSEVILAED<br>KRTQKLVAIKCIAKEALEGKEGSMENEIAVLHKIKHPNIVA<br>LDDIYESGGHLYLIMQLVSGGELFDRIVEKGFYTERDASRLI<br>FQVLDAVKYLHDLGIVHRDLKPENLLYYSLDEDSKIMISDF<br>GLSKMEDPGSVLSTACGTPGYVAPEVLAQKPYSKAVDC<br>WSIGVIAYILLCGYPPFYDENDAKLFEQILKAEYEFDSPYW<br>DDISDSAKDFIRHLMEKDPEKRFTCEQALQHPWIAGDTA<br>LDKNIHQSVSEQIKKNFAKSKWKQAFNATAVVRHMRKL<br>QLGTSQEGQGQTASHGELLTPVAGGPAAGCCCRDCCV<br>EPGTELSPTLPHQLEHHHHHH	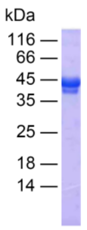	Group1<br>_batch2

Q13185	CBX3	1	183	MASNKTTLQKMGKKQNGKSKKVEEAEPEEFVVEKVLDRRVVNGKVEYFLKWKGFTDADNTWEPEENLDCPELIEAFLNSQKAGKEKDGTKRKSLSDSESDDSKSKKKRDAADKPRGFARGLDPERIIGATDSSGELMFLMKWKDSDEADLVLAKEANMKCPQIVIAFYEERLTWHSCPEDEAQLEHHHHHH	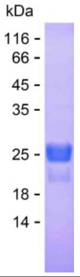	Group1_batch2
Q8WUD4	CCDC12	1	166	MEATTAGVGRLEEEALRRKERLKALREKTGRKDKEDGEPKTKHLREEEEEGEKHRELRLRNYVPEDEDLKKRRVPQAKPVAVEEKVKEQLEAAKPEPVIEEVDLANLAPRKPDWDLKRDVAKKLEKLKKRTQRAIAELIRERLKGQEDSLASAVDAATEQKTCDSDLEHHHHHH	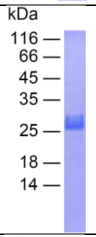	Group1_batch2
P32320	CDA	1	146	MAQKRPACTLKPECVQQLLVCSQEAKKSAYCPYSHFPVGAALLTQEGRIFKGCNIENACYPLGICAERTAIQKAVSEGYKDFRAIAIASDMQDDFISPCGACRQVMREFGTNWPVYMTKPDGTYIVMTVQELLPSSFGPEDLQKTQLEHHHHHH	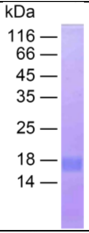	Group7_batch2
P60953	CDC42	1	188	MQTIKCVVVGDGAVGKTCLLISYTTNKFPSEYVPTVFDNYAVTVMIGGEPYTLGLFDTAGQEDYDRLRPLSYPQTDVFLVCFSVVSPSSFENVKEKWVPEITHHCPKTPFLLVGTQIDLRDDPSTIEKLAKNKQKPITPETAEKLARDLKAVKYVECSALTQKGLKNVFDEAILAALEPPEPKKSRRCLEHHHHHH	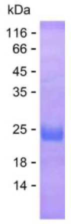	Group2_batch2
P54105	CLNS1A	1	237	MSFLKSFPPPGPAEGLLRQQPDTEAVLNGKGLGTGTLYIAESRLSWLDGSGLGFSLEYPTISLHALSRDRSDCLGEHLYVMVNAKFEEESKEPVADEEEEDSDDDVEPITEFRFVPSDKSALEAMFTAMCECQALHPDPEDEDSDDYDGEEYDVEAHEQGQGDIPTFYTYEEGLSHLTAEGQATLERLEGMLSQSVSSQYNMAGVRTEDSIRDYEDGMEVDTTPTVAGQFEDADVDHLEHHHHHH	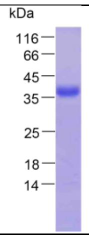	Group5_batch2
P41240	CSK	1	450	MSAIQAAWPSGTECIAKYNFHGTAEQDLPFCKGDVLTIVAVTKDPNWYKAKNKVGREGIIPANYVQKREGVKAGTKLSLMPWFHGKITREQAERLLYPPETGLFLVRESTNYPGDYTLCVSCDGKVEHYRIMYHASKLSIDEEVYFENLMQLVEHYTSDADGLCTRLIKPKVMEGTVAAQDEFYRSGWALNMKELKLLQTIGKGEFGDVMLGDYRGNKVAVKCIKNDATAQAFLAEASVMTQLRHSNLVQLLGVIVEEKGGLYIVTEYMAKGSLVDYLRSRGRSVLGGDCLLKFSLDVCEAMEYLEGNNFVHRDLAARNVLVSEDNVAKVSDFGLTKEASSTQDTGKLPVKWTAPEALREKKFSTKSDVWSFGILLWEIYSFGRVPYPRIPLKDVVPRVEKGYKMDAPDGCPPAVYEVMKNCWHLDAAMRPSFLQLREQLEHIKTHELHLEHHHHHH	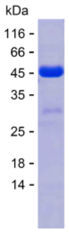	Group6_batch2
Q9ULA0	DNPEP	1	475	MQVAMNGKARKEAVQTAAKELLKFVNRSPSPFHAVAECRNRLLQAGFSELKETEKWNIKPESKYFMTRNSSTIIAFAVGGQYVPGNGFSLIGAHTDSPCLRVKRRSRRSQVGFQQVGVETYGGGIWSTWFDRDLTLAGRVIVKCPTSGRLEQQLVHVERPILRIPHLAIHLQRNINENFGPNTEMHLVPILATAIQEELEKGTPEPGPLNAVDERHHSVLMSLLCAHLGLSPKDIVEMELCLADTQPAVLGGAYDEFIFAPRLDNLHSCFCALQALIDSCAGPGSLATEPHVRMVTLYDNEEVGSESAQGAQSLLTELVLRRISASCQHPTAFEEAIPKSFMISADMAHAVHPNYLDKHEENHRPLFHKGPVIKVNSKQRYASNAVSEALIREVA	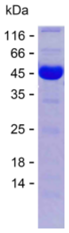	Group5_batch2

				NKVKVPLQDLMVRNDTPCGTTIGPILASRLGLRVLDLGSP<br>QLAMHSIREMACTTGVLQTLTLFKGFFELFPSLSHNLLVD<br>LEHHHHHH		
Q16555	DPYSL2	1	572	MSYQGKKNIPRITSDRLLIKGGKIVNDDQSFYADIYMEDG<br>LIKQIGENLIVPGGVKTIEAHSRMVIPGGIDVHTRFQMPD<br>QGMTSADDFFQGTKAALAGGTTMIIDHVVPEPGTSLLAA<br>FDQWREWADSKSCCDYSLHVDISEWHKGIQEEMEALVK<br>DHGVNSFLVYMAFKDRFQLTDCQIYEVLSVIRDIGAIAQV<br>HAENGDIIAEEQQRILDLGITGPEGHVLSRPEEVEAEAVN<br>RAITIANQTNCPLYITKVMSKSSAEVIAQARKKGTVVYGEP<br>ITASLGTDGSHYWSKNWAKAAAFVTSPPLSPDPTTPDFL<br>NSLLSCGDLQVTGSAHCTFNTAQKAVGKDNFTLIPEGTN<br>GTEERMSVIWDKAVVTGKMDENQFVAVTSTNAAKVFNL<br>YPRKGRIAVGSDADLVIWDPDSVKTISAKTHNSSLEYNIFE<br>GMECRGSPLVVISQGKIVLEDGTLHVTEGSGRYIPRKPFP<br>DFVYKRIKARSRLAELRGVPRGLYDGPVCEVSVTPKTVTP<br>ASSAKTSPAKQQAPPVRNLHQSGFSLSGAQIDDNIPRRT<br>TQRIVAPPGGRANITSLGLEHHHHHH	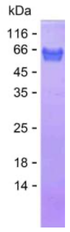	Group8<br>_batch2
P55010	EIF5	1	431	MSVNVNRSVSDQFYRYKMPRLIAKVEGKGNGIKTVIVN<br>MVDVAKALNRPPTYPTKYFGCELGAQTQFDVKNDRYIV<br>NGSHEANKLQDMLDGFIKKFVLCPECENPETDLHVNPKK<br>QTIGNSCKACGYRGMLDTHHKLCTFILKNPPENSDSGTG<br>KKEKEKKNRKGKDKENGSVSSSETPPPPPPPNEINPPPHT<br>MEEEEDDDWGEDTTEEAQRRRMDEISDHAKVLTLSDDL<br>ERTIEERVNILFDFVKKKKEEGVIDSSDKEIVAEAERLDVKA<br>MGPLVLTEVLFNEKIREQIKKYRRHFLRFCHNNKKAQRYL<br>LHGLECVVAMHQAQLISKIPHILKEMYDADLLEEEVIISWS<br>EKASKKYVSKELAKEIRVKAEPFIKWLKEAEEESSGGEEEDE<br>DENIEVVYSKAASVPKVETVKSDNKDDDIDIDAILEHHHH<br>HH	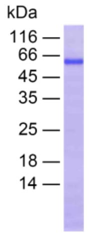	Group1<br>_batch2
Q9GZV4	EIF5A2	1	153	MADEIDFTTGDAGASSTYPMQCSALRKNGFVVLKGRPC<br>KIVEMSTSKTGKHGHAKVHLVGIDIFTGKKYEDICPSTHN<br>MDVPNIKRNDYQLICIQDGYLSLLTETGEVREDLKLPEGEL<br>GKEIEGKYNAGEDVQVSVMCAMSEEYAVAIKPCKLEHH<br>HHHH	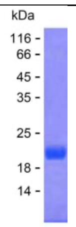	Group3<br>_batch2
A6PVY3	FAM177B	1	158	MEIDGFQQLDLEKSVPSKKTTPKRIIHFVDGDIMEEYSTEE<br>EEEEEKEEQSTNSTLDPSKLSWGPYLRFWAGRIASTSFSTC<br>EFLGGRFAVFFGLTQPKYQYVLNEFYRIQNKKSDNKSERR<br>GSKAQAAEVPNEKCHLEAGVQEYGTIQQDVTEAIPQLEH<br>HHHHH	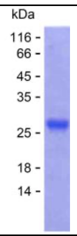	Group8<br>_batch2
P07954	FH	45	510	MASQNSFRIEYDTFGELKVPNDKYYGAQTVRSTMNFKIG<br>GVTERMPTPVIKAFGILKRAAAEVNQDYGLDPKIANAIMK<br>AADEVAEGKLNDHFPLVVWQTGSGTQTNMNVNEVISN<br>RAIEMLGGELGSKIPVHPNDHVNKSQSSNDTFPTAMHIA<br>AAIEVHEVLLPGLQKLHDALDAKSKEFAQIIKIGRTHTQDA<br>VPLTLGQEFSGYVQQVKYAMTRIKAAMPRIYELAAGGTA<br>VGTGLNTRIGFAEKVAAKVAALTGLPFVTAPNKFEALAAH<br>DALVELSGAMNTTACSLMKIANDIRFLGSGPRSGLGELIL<br>PENEPGSSIMPGKVNPTQCEAMTMVAAQVMGNHVAV<br>TVGGSNGHFELNVFKPMMIKNVLHSARLLGDASVSFTEN<br>CVVGIQANTERINKLMNESLMLVTALNPHIGYDKAAKIAK<br>TAHKNGSTLKETAIELGYLTAEQFDEWVKPKDMLGPKLE<br>HHHHHH	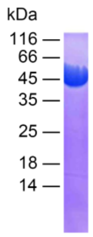	Group8<br>_batch2

A8MYZ6	FOXO6	1	492	MAAKLRAHQVDVDPDFAPQSRPRSCTWPLPQPDLAGD<br>EDGALGAGVAEGAEDCGPERRATAPAMAPAPPLGAEVG<br>PLRKAKSSRRNAWGNLSYADLITKAIESAPDKRLTLSQIYD<br>WMVRYVPYFKDKGDSNSSAGWKNSIRHNLSLHTRFIRV<br>QNEGTGKSSWWMLNPEGGKTGKTPRRRAVSMDNGAK<br>FLRIKGKASKKKQLQAPERSPDDSSPSAPAPGPVPAAAK<br>WAASPASHASDDYEAWADFRGGGRPLLGEAAELEDDE<br>ALEALAPSSPLMYPSPASALSPALGSRCPGELPRLAELGGP<br>LGLHGGGGAGLPEGLLDGAQDAYGPRPAPRPGPVLGAP<br>GELALAGAAAAYPGKGAAPYAPPAPSRSALAHPISLMTL<br>PGEAGAAGLAPPGHAAAFGGPPGGLLLDALPGPYAAAA<br>AGPLGAAPDRFPADLDLDMFSGSLECDVESIILNDFMDS<br>DEMDFNFDSALPPPPPGLAGAPPPNQSWVPGLEHHHH<br>HH	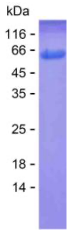	Group5<br>_batch2
O95257	GADD45G	1	159	MTLEEVRGQDTVPESTARMQGAGKALHELLLSAQRQGC<br>LTAGVYESAKVLNVDPDNVTFCVLAAGEEDEGDIALQIHF<br>TLIQAFCCENDIDIVRVGDVQRLAAIVGAGEEAGAPGDL<br>HCILISNPNEDAWKDPALEKLSLFCEESRSVNDWVPSITLP<br>ELEHHHHHH	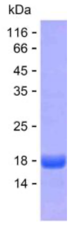	Group2<br>_batch2
Q13630	GFUS	1	321	MGEPQGSMRILVTGGSGLVGKAIQKVVADGAGLPGED<br>WVFVSSKDADLTDTAQTRALFEKVQPTHVIHLAAMVGG<br>LFRNIKYNLDFWRKNVHMNDNVLHSAFEVGARKVVSCL<br>STCIFPDKTTYPIDETMIHNGPPHNSNFGYSYAKRMIDVQ<br>NRAYFQQYGCTFTAVIPTNVFGPHDNFNIEDGHVLPGLI<br>HKVHLAKSSGSALTVWGTGNPRRQFIYSLDLAQLFIWVL<br>REYNEVEPIILSVGEEDEVSIKEAAEAVVEAMDFHGEVTFD<br>TTKSDGQFKKTASNSKLRTYLPDFRFTPFKQAVKETCAWF<br>TDNYEQARKLEHHHHHH	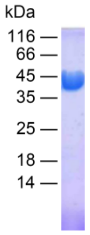	Group1<br>_batch2
P00505	GOT2	30	430	MSSWWTHVEMGPPDPILGVTEAFKRDTNSKKMNLGVG<br>AYRDDNGKPYVLPSVRKAEAQIAAKNLDKEYLPIGGLAEF<br>CKASAELALGENSEVLKSGRFVTVQTISGTGALRIGASFLQ<br>RFFKFSRDVFLPKPTWGNHTPIFRDAGMQLQGYRYYDPK<br>TCGFDFTGAVEDISKIPEQSVLLLHACAHNPTGVDPRPEQ<br>WKEIATVVKKRNLFAFFDMAYQGFASGDGDKDAWAVR<br>HFIEQGINVCLCQSYAKNMGLYGERVGAFTMVCKDADE<br>AKRVESQLKILIRPMYSNPPLNGARIAAAILNTPDLRKQW<br>LQEVKVMADRIIGMRTQLVSNLKKEGSTHNWQHITDQI<br>GMFCFTGLKPEQVERLIKEFSIYMTKDGRISVAGVTSSNV<br>GYLAHAIHQVTKLEHHHHHH	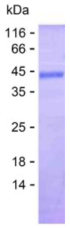	Group7<br>_batch2
P00390	GSR	44	522	MACRQEPQPQGPPPAAGAVASYDYLVIGGGSGGLASAR<br>RAAELGARAAVVESHKLGGTCVNVGCVPKKVMWNTAV<br>HSEFMHDHADYGFPSCEGKFNWRVIKEKRDAYVSRLNAI<br>YQNNLTKSHIEIIRGHAAFTSDPKPTIEVSGKKYTAPHILIAT<br>GGMPSTPHESQIPGASLGITSDGFFQLEELPGRSVIVGAG<br>YIAVEMAGILSALGSKTSLMIRHDKVLRSFDSMISTNCTEE<br>LENAGVEVLKFSQVKEVKKTLSGLEVSMVTAVPGRLPVM<br>TMIPDVDCLLWAIGRVPNTKDLSLNKLGIQTDDKGHIIVD<br>EFQNTNVKGIYAVGDVCGKALLTPVAIAAGRKLAHRLFEY<br>KEDSKLDYNNIPTVVFSHPPIGTVGLTEDEAIHKYGIENVK<br>TYSTSFTPMYHAVTKRKTKCVMKMVCANKEEKVVGIHM<br>QGLGCDEMLQGFAVAVKMGATKADFDNTVAIHPTSSEE<br>LVTLRLEHHHHHH	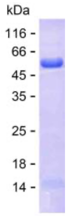	Group5<br>_batch2

P0CG29	GSTT2	1	244	MGLELFLDLVSQPSRAVYIFAKKNGIPLELRTVDLVKGQHKSKEFLQINSLGKLPTLKDGDFILTESSAILIYLSCKYQTPDHWYPSDLQARARVHEYLGWHADCIRGTFGIPLWVQVLGPLIGVQVPKEKVERNRTAMDQALQWLEDKFLGDRPFLAGQQVTLADLMALEELMQPVALGYELFEGRPRLAAWRGRVEAFLGAELCQEAHSIILSILEQAAKKTLPTPSPEAYQAMLLRIARIPLEHHHHHH	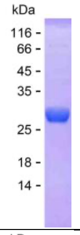	Group3_batch2
O75409	H2AP	1	117	MSEKKNCKNSSTNNNQTQDPSRNELQVPRSFVDRVVQDERDVQSQSSSTINTLLTLLDCLADYIMERVGLEASNNGSMRNTSQDREREVDNNREPHSAESDVTRFLFDEMPKSRKNDLEHHHHHH	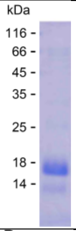	Group4_batch2
P01112	HRAS	1	186	MTEYKLVVVGAGGVGKSALTIQLIQNHFVDEYDPTIEDSYRKQVVIDGETCLLDILDTAGQEEYSAMRDQYMRTGEGFLCVFAINNTKSFEDIHQYREQIKRVKDSDDVPMVLVGNKCDLAARTVESRQAQDLARSYGIPYIETSAKTRQGVEDAFYTLVREIRQHKLRKLNPPDESGPGCMSCKCLEHHHHHH	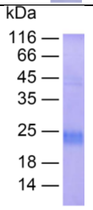	Group8_batch2
Q53T59	HS1BP3	1	392	MQSPAVLVTSRRLQNAHTGLDLTVPQHQEVRGKMMSGHVEYQILVVTRLAAFKSAKHRPEDVVQFLVSKKYSEIEEFYQKLSSRYAAASLPPLPRKVLFVGESDIRERRAVFNEILRCVSKDAELLAGSPELLEFLGTRSPGAAGLTSRDSSVLDGTDSQTGNDEEAFDFFEEQDQVAEEGPPVQSLKGEDAEESLEEEEALDPLGIMRSKKPKKHPKVAVKAKPSPRLTIFDEEVDPDEGLFGPGRKLSPQDPSEDVSSVDPLKLFDDPDLGGAIPLGDSLLLPAACESGGPTPSLSHRDASKELFRVEEDLDQILNLGAEPKPKPQLKPKPPVAAKPVIPRKPAVPPKAGPAEAVAGQQKPQEQIQAMDEMDILQYIQDHDTPAQAAPSLFLEHHHHHH	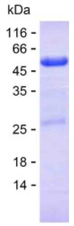	Group7_batch2
P09914	IFIT1	1	478	MSTNGDDHQVKDSLEQLRCHFTWELSIDDDEMPDLENRVLDQIEFLDTKYSVGIHNLLAYVKHLKGQNEEALKSLKEAENLMQEEHDNQANVRSLVTWGNFAWMYYHMGRLAEAQTYLDKVENICKKLSNPFRYRMECPEIDCEEGWALLKCGGKNYERAKACFEKVLEVDPENPESSAGYAISAYRLDGFKLATKNHKPFSLLPLRQAVRLNPDNGYIKVLLALKLQDEGQEAEGEKYIEEALANMSSQTYVFRYAAKFYRRKGSVDKALELLKKALQETPTSVLLHHQIGLCYKAQMIQIKEATKGQPRGQNREKLDKMIRSAIFHFESAVEKKPTFEVAHLDLARMYIEAGNHRKAEENFQKLLCMKPVVEETMQDIHFHYGRFQEFQKKSDVNAIIHYLKAIKIEQASLTRDKSINSLKKLVLRKLRRKALDLESLSLLGFVYKLEGNMNEALEYYERALRLAADFENSVRQGPLEHHHHHH	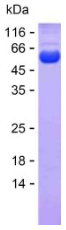	Group8_batch2
Q3ZCW2	LGALSL	1	172	MAGSVADSDAVVKLDDGHLNNSLSSPVQADVYFPRLIVPFCGHIKGGMRPGKKVLVMGIVDLNPESFAISLTCGDSEDPPADVAIELKAVFTDRQLLRNSCISGERGEEQSAIPYFPFIPDQPFRVEILCEHPRFRVFVDGHQLFDFYHRIQTLSAIDTIKINGDLQITKLGLEHHHHHH	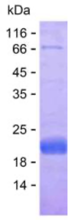	Group6_batch2
Q3MHD2	LSM12	1	195	MAAPPGEYFSVGSQVSCRTCQEQRLQGEVVAFDYQSKMLALKCPSSSGKPNHADILLINLQYVSEVEIINDRTETPPPLASLNVSKLASKARTEKEEKLSQAYAISAGVSLEGQQLFQTIHKTIKDCKWQEKNIVVMEEVVITPPYQVENCKGKEGSALSHVRKIVEKHFRDVESQKILQRSQAQQPQKEAALSSLEHHHHHH	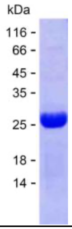	Group4_batch2

Q9UGB7	MIOX	1	285	MKVTVGPDPSLVYRPDVDPEVAKDKASFRNYTSGPLLDRVFTTYKLMHTHQTVDFVRSKHAQFGGFSYKKMTVMEAVDLLDGLVDESDPDVDFPNSFHAFQTAEGIRKAHPDKDWFHLVGLLHDLGKVLALFGEPQWAVVGDTFPVGCRPQASVVFCDSTFQDNPDLQDPRYSTELGMYQPHCGLDRVLMSWGHDEYMYQVMKFNKFSLPPEAFYMIRFHSFYPWHTGRDYQQLCSQQDLAMLPWVREFNKFDLYTKCPDLPDVDKLRPYYQGLIDKYCPGILSWLEHHHHHH	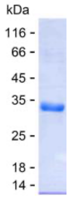	Group2 _batch2
P29372	MPG	18	298	MGQKKQRPARAGQPHSSSDAAQAPAEQPHSSSDAAQAPCPRERCLGPPTTPGPYRSIYFSSPKGHLTRLGLEFFDQPAVPLARAFLGQVLVRRLPNGTELRGRIVETEAYLGPEDEAAHSRGGRQTPRNRGMFMKPGTLYVYIIYGMYFCMNISSQGDGACVLLRALEPLEGLETMRQLRSTLRKGTASRVLKDRELCSGPSKLCQALAINKSFDQRDLAQDEAVWLERGPLEPSEPAVVAAARVGVGHAGEWARKPLRFYVRGSPWVSVVDRVAEQDTQALEHHHHHH	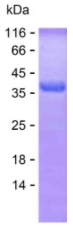	Group2 _batch2
P14598	NCF1	1	390	MGDTFIRHIALLGFEKRFVPSQHYVYMFLVKWQDLSEKVVYRRFTEIYEFHKTLKEMFPIEAGAINPENRIIPHLPAPKWFDGQRAAENRQGTLTEYCSTLMSLPTKISRCPHLLDFFKVRPDDLKLPTDNQTKKPETYLMPKDGKSTATDITGPIILQTYRAIANYEKTSGSEMALSTGDVVEVVEKSESGWWFCQMKAKRGWIPASFLEPLDSPDETEDPEPNYAGEPYVAIKAYTAVEGDEVSLLEGEAVEVIHKLLDGWWVIRKDDVTGYFPSMYLQKSGQDVSQAQRQIKRGAPPRRSSIRNAHSIHQRSRKRLSQDAYRRNSVRFLQQRRRQARPGPQSPGSPLEEERQTQRSKPQPAVPPRPSADLILNRCSESTKRKLASAVLEHHHHHH	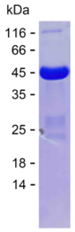	Group2 _batch2
A6NI72	NCF1B	1	391	MGDTFIRHIALLGFEKRFVPSQHYVRYMFLVKWQDLSEKVVYRRFTEIYEFHKTLKEMFPIEAGAINPENRIIPHLPAPKWFDGQRAAENHQGTLTEYCGTLMSLPTKISRCPHLLDFFKVRPDDLKLPTDNQTKKPETYLMPKDGKSTATDITGPIILQTYRAIANYEKTSGSEMALSTGDVVEVVEKSESGWWFCQMKAKRGWIPASFLEPLDSPDETEDPEPNYAGEPYVAIKAYTAVEGDEVSLLEGEAVEVIHKLLDGWWVIRKDDVTGYFPSMYLQKSGQDVSQAQRQIKRGAPPRRSSIRNVHSIHQRSRKRLSQDAYRRNSVRFLQQRRRQARPGPQSPGSPLEEERQTQRSKPQPAVPPRPSADLILNRCSESTKRKLASAVLEHHHHHH	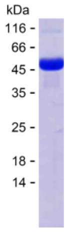	Group6 _batch2
Q9NX24	NHP2	1	153	MTKIKADPDGPEAQAEACSGERTYQELLVNQNPIAQPLASRRLTRKLYKCIKKAVKQKQIRRGVKEVQKFVNKGEKGIMVLAGDTLPIEVYCHLPVMCEDRNLPYVYIPSKTDLGAAAGSKRPTCVIMVKPHEEYQEAYDECLEEVQSLPLPLEHHHHHH	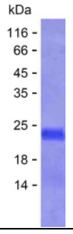	Group3 _batch2
Q96AB6	NTAN1	1	310	MPLLVEGRRVRLPQSAGDLVRAHPPLEERARLLRGQSVQQVGPQGLLYVQQRELAVTSPKDGSISILGSDDATTCHIVVLRHTGNGATCLTHCDGTDTKAEVPLIMNSIKSFSDHAQCGRLEVHLVGGFSDDRQLSQKLTHQLLSEFDRQEDDIHLVTLCVTELNDREENENHFPVIYGIAVNIKTAEIYRASFQDRGPEEQLRAARTLAGGPMISIYDAETEQLRIGPYSWTPFPHVDFWLHQDDKQILENLSTSPLAEPPHFVEHIRSTLMFLKKHPSPAHTLFSGNKALLYKKNEDGLWEKISSPGSLEHHHHHH	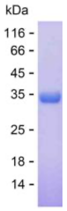	Group1 _batch2

P61970	NUTF2	1	127	MGDKPIWEQIGSSFIQHYYQLFDNDRTQLGAIYIDASCLT<br>WEGQQFQGKAAIVEKLSSLPFQKIQHSITAQDHQPTPDS<br>CIISMVVGQLKADEDPIMGFHQMFLLKNINDAWVCTND<br>MFRLALHNFGLEHHHHHH	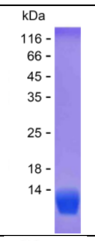	Group3<br>_batch2
Q16625	OCLN	270	522	MKMDRYDKSNILWDKEHIYDEQPPNVEEWVKNVSAGT<br>QDVPSPPSDYVERVDSPMAYSSNGKVNDKRFYPESSYKS<br>TPVPEVVQELPLTSPVDDFRQPRYSSGGNFETPSKRAPAK<br>GRAGRSKRTEQDHYETDYTTGGESCDELEEDWIREYPPIT<br>SDQQRQLYKRNFDTGLQEYKSLQSELDEINKELSRLDKEL<br>DDYREESEEYMAAADEYNRLKQVKGSADYKSKKNHCKQ<br>LKSKLSHIKKMVGDYDRQKTLEHHHHHH	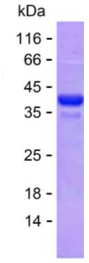	Group1<br>_batch2
P68402	PAFAH1B2	1	229	MSQGDSNPAAIPHAAEDIQGDDRWMSQHNRFVLDCK<br>DKEPDVLFVGDSMVQLMQQYEIWRELFSPLHALNFGIG<br>GDTTRHVLWRLKNGELENIKPKVIVVWVGTNNHENTAE<br>EVAGGIEAIVQLINTRQPQAKIIVLGLLPRGEKPNPLRQKN<br>AKVNQLLKVSLPKLANVQLLDTDGGFVHSDGAISCHDM<br>FDFLHLTGGGYAKICKPLHELIMQLLEETPEEKQTTIALEH<br>HHHHH	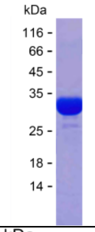	Group1<br>_batch2
Q9BUL8	PDCD10	1	212	MRMTMEEMKNEAETTSMVSMPLYAVMYPVFNELERVN<br>LSAAQTLRAAFIKAEKENPGLTQDIIMKILEKKSVEVNFTES<br>LLRMAADDVEEYMIERPEPEFQDLNEKARALKQILSKIPDE<br>INDRVRFLQTIKDIASAIKELLDTVNNVFKKYQYQNRRALE<br>HQKKEFVKYSKSFSDTLKTYFKDGKAINVFVSANRLIHQT<br>NLILQTFKTVALEHHHHHH	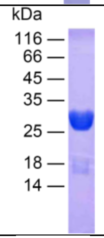	Group6<br>_batch2
P07737	PFN1	1	140	MAGWNAYIDNLMADGTCQDAAIVGYKDSPSVWAAVP<br>GKTFVNITPAEVGVLVGKDRSSFYVNGLTLGGQKCSVIRD<br>SLLQDGEFSMDLRTKSTGGAPTFNVTVTKTDKTLVLLMG<br>KEGVHGGLINKKCYEMASHLRRSQYLEHHHHHH	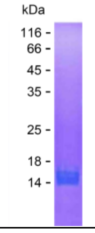	Group2<br>_batch2
Q8IUZ5	PHYKPL	1	450	MAADQRPKADTLALRQRLISSSCRLFFPEDPVKIVRAQG<br>QYMYDEQGAEYIDCISNVAHVGHCHPLVVQAAHEQNQ<br>VLNTNSRYLHDNIVDYAQRLSETLPEQLCVFYFLNSGSEA<br>NDLALRLARHYTGHQDVVVLDHAYHGHLSSLIDISPYKF<br>RNLDGQKEWVHVAPLPDTYRGPYREDHPNPAMAYANE<br>VKRVVSSAQEKGRKIAAFFAESLPSVGGQIIPPAGYFSQVA<br>EHIRKAGGVFVADEIQVGFGRVGKHFWAFQLQGKDFVP<br>DIVTMGKSIGNGHPVACVAATQPVARAFEATGVEYFNTF<br>GGSPVSCAVGLAVLNVLEKEQLQDHATSVGSFLMQLLG<br>QQKIKHPIVGDVRGVGLFIGVDLIKDEATRTPATEEAAYLV<br>SRLKENYVLLSTDGPGRNILKFKPPMCFSLDNARQVVAKL<br>DAILTDMEEKVRSCETLRLQPLEHHHHHH	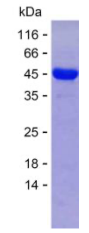	Group5<br>_batch2
P25788	PSMA3	1	255	MSSIGTGYDLSASTFSPDGRVFQVEYAMKAVENSSTAIGI<br>RCKDGVVFGVEKLVLSKLYEEGSNKRLFNVDRHVGMAV<br>AGLLADARSLADIAREEASNFRSNFGYNIPLKHLADRVA<br>MYVHAYTLYSAVRPFGCSFMLGSYSVNDGAQLYMIDPS<br>GVSYGYWGCAIGKARQAAKTEIEKLQMKEMTCRDIVKEV<br>AKIIYIVHDEVKDKAFELELSWVGELTNGRHEIVPKDIREEA<br>EKYAKESLKEEDESDDDNMLEHHHHHH	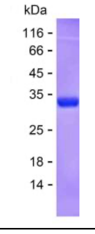	Group2<br>_batch2

P15153	RAC2	1	189	MQAIKCVVVGDGAVGKTCLLISYTTNAFPGEYIPTVFDNYSANVMVDSKPVNLGLWDTAGQEDYDRLRPLSYPQTDVFLICFSLVSPASYENVRAKWFPEVRHHCPSTPIILVGTKLDLRDDKDTIEKLKEKKLAPITYPQGLALAKEIDSVKYLECSALTQRGLKTVFDEAIRAVLCPQPTRQQKRACLEHHHHHH	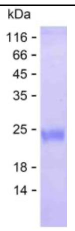	Group5_batch2
P52758	RIDA	1	137	MSSLIRRVISTAKAPGAIGPYSQAVLVDRTIYISGQIGMDPSSGQLVSGGVAEEAKQALKNMGEILKAAGCDFTNVVKTTVLLADINDFNTVNEIYKQYFKSNFPARAAYQVAALPKGSRIEIEAVAIQGPLTTASLEHHHHHH	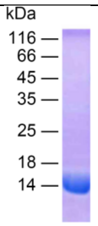	Group3_batch2
Q9UJC5	SH3BGRL2	1	107	MVIRVFIASSSGFVAIKKKQQDVVRFLEANKIEFEEVDITMSEEQRQWMYKNVPPEKKPTQGNPLPPQIFNGDRYCGDYDSFFESKESNTVFSFLGLKPRLASKAEPLEHHHHHH	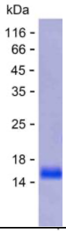	Group7_batch2
O00337	SLC28A1	1	80	MENDPSRRRESISLTPVAKGLENMGADFLESLEEGQLPRSDLSPAEIRSSWSEAAPKPFSRWRNLQPALRARSFCREHMQLEHHHHHH	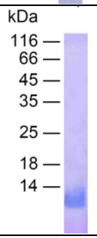	Group4_batch2
P52788	SMS	1	366	MAAARHSTLDFMLGAKADGETILKGLQSIFQEQGMAESVHTWQDHGYLATYTNKNGSFANLRIYPHGLVLLDLQSYDGDAQGKEEIDSILNKVEERMKELSQDSTGRVKRLPPIVRGGAIDRYWPTADGRLVEYDIDEVVYDEDSPYQNIKILHSKQFGNILILSGDVNLAESDLAYTRAIMGSGKEDYTGKDVLILGGGDGGILCEIVKLKPKMVTMVEIDQMVIDGCKKYMRKTCGDVLDNLKGDCYQVLIEDCIPVLKRYAKEGREFDYVINDLTAVPISTSPEEDSTWEFLRLILDLSMKVLKQDGKYFTQGNCVNLTEALSLYEEQLGRLYCPVEFSKEIVCVPSYLELWVFYTVWKKAKPLEHHHHHH	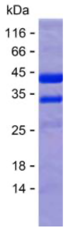	Group4_batch2
Q5T280	SPOUT1	1	376	MAERGRKRPCGPGEHGQRIEWRKWKQQKKEEKKKWKDLKLMKKLERQRAQEEQAKRLEEEEAAAEKEDRGRPYTLSVALPGSILDNAQSPELRTYLAGQIARACAIFCVDEIVVFDEEGQDAKTVEGEFTGVGKKGQACVQLARILQYLECPQYLRKAFFPKHQDLQFAGLLNPLDSPHHMRQDEESEFREGIVVDRPTRPGHGSFVNCGMKKEVKIDKNLEPGLRVTVRLNQQQHPDCKTYHGKVVSSQDPRTKAGLYWGYTVRLASCLSAVFAEAPFQDGYDLTIGTSERGSDVASAQLPNFRHALVVFGGLQGLEAGADADPNLEVAEPSVLFDLYVNTCPGQGSRTIRTEEAILISLAALQPGLIQAGARHTLEHHHHHH	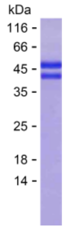	Group7_batch2
P22528	SPRR1B	1	89	MSSQQQKQPCTPPPQLQQQQVKQPCQPPPQEPCIPKTKEPCHPKVPEPCHPKVPEPCQPKVPEPCHPKVPEPCPSIVTPAPAQQKTKQKLEHHHHHH	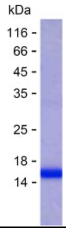	Group6_batch2

O43805	SSNA1	1	119	MTQQGAALQNYNNELVKCIEELCQKREELCRQIQEEEDEKQRLQNEVRQLTEKLARVNENLARKIASRNEFDRTIAETEAAYLKILESSQTLLSVLKREAGNLTKATAPDQKSSGGRDSLEHHHHHH	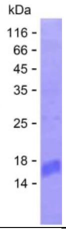	Group4 _batch2
Q7Z422	SZRD1	1	152	MEDEEVAESWEEAADSGEIDRRLEKKLKITQKESRKSKSPPKVPIVIQDDSLPAGPPPQIRILKRPTSNGVVSSPNSTSRPTLPVKSLAQREAEYAEARKRILGSASPEEEQEKPILDRPTRISQPEDSRQPNNVIRQPLGPDGSQGFKQRRLEHHHHHH	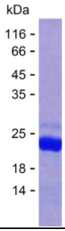	Group8 _batch2
Q53FA7	TP53I3	1	332	MLAVHFDKPGGPENLYVKEVAKPSPGEGEVLLKVAASALNRADLMQRQGQYDPPPGASNILGLEASGHVAELGPGCQGHWKIGDTAMALLPGGGQAQYVTVPEGLLMPIPEGLTLTQAAAIPEAWLTAFQLLHLVGNVQAGDYVLIHAGLSGVGTAAIQLTRMAGAIPLVTAGSQKKLQMAEKLGAAAGFNYKKEDFSEATLKFTKGAGVNLILDCIGGSYWEKNVNCLALDGRWVLYGLMGGGDINGPLFSKLLFKRGSLITSLLRSRDNKYKQMLVNAFTEQILPHFSTEGPQRLLPVLDRIYPVTEIQEAHKYMEANKNIGKIVLELPQLEHHHHHH	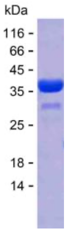	Group4 _batch2
O43617	TRAPPC3	1	180	MSRQANRGTESKKMSSELFTLTYGALVTQLCKDYENDEDVNKQLDKMGFNIGVRLIEDFLARSNVGRCHDFRETADVIAKVAFKMYLGITPSITNWSPAGDEFSLILENNPLVDFVELPDNHSSLIYSNLLCGVLRGALEMVQMAVEAKFVQDTLKGDGVTEIRMRFIRRIEDNLPAGEELEHHHHHH	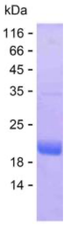	Group6 _batch2
Q9BZA5	TXLNGY	1	131	MEEAGLCGLREKADMLCNSESHDILQHQDSNCSATSNKHLLEDEEGRDFITKNRSWVSPVHCTQESRRELPEQEVAPPSGQQALQCNRNKEKVLGKEVLLLMQALNTLSTPEEKLAALCKKYADLGNSPLLEHHHHHH	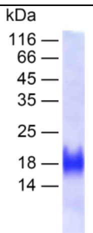	Group5 _batch2
P61077	UBE2D3	1	147	MALKRINKELSDLARDPPAQCSAGPVGDDMFHWQATIMGPNDSPYQGGVFFLTIHFPTDYPFKPPKVAFTTRIYHPNINSNGSICLDILRSQWSPALTISKVLLSICSLLCDPNPDDPLVPEIARIYKTDRDKYNRISREWTQKYAMLEHHHHHH	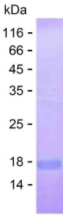	Group4 _batch2
P11441	UBL4A	1	157	MQLTVKALQGRECSLQVPEDELVSTLKQLVSEKLNVPVRQQRLLFKGKALADGKRLSDYSIGPNSKLNLVVKPLEKVLLEEGEAQRLADSPPPQVWQLISKVLARHFSAADASRVLEQLQRDYERSLSRLTLDDIERLASRFLHPEVTETMEKGFSKLEHHHHHH	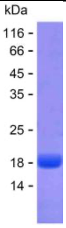	Group7 _batch2
Q9BRT2	UQCC2	14	126	MEEWPVDETKRGRDLGAYLRQRVAQAFREGENTQVAEPEACDQMYESLARLHSNYYKHKYPRPRDTSFSGLSLEEYKLILSTDTLEELKEIDKGMWKKLQEKFAPKGPEEDHKALEHHHHHH	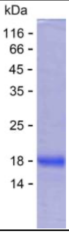	Group7 _batch2

Q4G0F5	VPS26B	1	336	MSFFGFGQSVEVEILLNDAESRKRAEHKTEDGKKEKYFLFYDGETVSGKVSLALKNPNKRLEHQGIKIEFIGQIELYYDRGNHHEFVSLVKDLARPGEITQSQAFDFEFTHVEKPYESYTGQNVKLRYFLRATISRRLNDVVKEMDIVVHTLSTYPELNSSIKMEVGIEDCLHIEFEYNKSKYHLKDVIVGKIYFLLVRIKIKHMEIDIIKRETTGTGPNVYHENDTIAKYEIMDGAPVRGESIPIRLFLAGYELTPTMRDINKKFSVRYYLNLVLIDEEERRYFKQQEVVLWRKGDIVRKSMSHQAAIASQRFEGTTSLGEVRTPSQLSDNNCRQLEHHHHHH	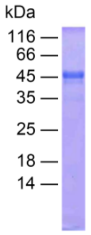	Group3 _batch2
Q5VTH2	CFAP126	1	177	MATNYSANQYEKAFSSKYLQNWSPTKPTKESISSHEGYTQIIANDRGHLLPSVPRSKANPWGSFMGTWQMPLKIPPARVTLTSRTTAGAASLTKWIQKNPDLLKASNGLCPEILGKPHDPDSQKKLRKKSITKTVQQARSPTIIPSSPAANLNSPDELQSSHPSAGHTPGPQRPAKSLEHHHHHH	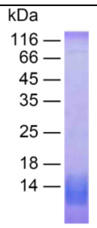	Group1 _batch3
A6NFE3	EFCAB10	1	127	METSSRELQAAEYLEKHQIKEVVSYLTSALLFFRPEKPKEYLISLLERLRIAKVTGVAFPFFMDNSNIVAMFEMMDSSGRGTISFVQYKEALKTLGLCTEDEDLQDDGHKITLDKFKEEVNKRMKEILEHHHHHH	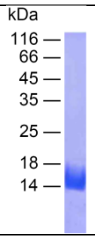	Group1 _batch1
